# Twenty-one new species of the *Simulium* (*Gomphostilbia*) *asakoae* species group (Diptera, Simuliidae) in Thailand, with their genetic relationships

**DOI:** 10.3897/zookeys.950.51298

**Published:** 2020-07-20

**Authors:** Hiroyuki Takaoka, Wichai Srisuka, Masako Fukuda, Atiporn Saeung

**Affiliations:** 1 Tropical Infectious Diseases Research and Education Centre (TIDREC), University of Malaya, 50603, Kuala Lumpur, Malaysia University of Malaya Kuala Lumpur Malaysia; 2 Entomology Section, Queen Sirikit Botanic Garden, P.O. Box 7, Maerim, Chiang Mai 50180, Thailand Queen Sirikit Botanic Garden Chiang Mai Thailand; 3 Institute for Research Promotion, Oita University, Idaigaoka 1-1, Hasama, Yufu City, Oita, 879-5593, Japan Oita University Oita Japan; 4 Center of Insect Vector Study, Department of Parasitology, Faculty of Medicine, Chiang Mai University, Chiang Mai 50200, Thailand Chiang Mai University Chiang Mai Thailand

**Keywords:** Aquatic insects, biodiversity, blackflies, Oriental Region, taxonomy

## Abstract

Females and males reared from pupae, their pupal exuviae and cocoons, and mature larvae of the Simulium (Gomphostilbia) asakoae species group from various localities in Thailand were morphologically examined. A total of 25 species was identified, including two of four known species (*Simulium asakoae* Takaoka & Davies and *S. chiangdaoense* Takaoka & Srisuka), one newly confirmed species (*S. myanmarense* Takaoka, Srisuka & Saeung, originally described from Myanmar), one newly transferred species (*S. inthanonense* Takaoka & Suzuki formerly of the *S. ceylonicum* species group), and 21 new species. Descriptions of all 21 new species are given, and the first full description of the male of *S. inthanonense*, together with the revised descriptions of its female, pupa, and larva, is also provided. Keys to identify all 27 members of this species group from Thailand are given for females, males, pupae, and larvae. The genetic relationships of all but one species were resolved using COI gene sequence-based analysis. All 26 species were divided into nine subgroups, I–IX, each consisting of two, one, four, nine, one, three, two, one and three species, respectively.

## Introduction

The *Simulium asakoae* species group, as defined by Takaoka (2012), is the second largest among the 14 species groups in Simulium (Gomphostilbia) (Adler 2019). It includes 36 species from various countries in the Oriental Region, of which 13 species have been recorded from Vietnam, eight species from Peninsular Malaysia, and four species from Thailand (Takaoka et al. 2017a, 2018, 2019; Srisuka et al. 2019). *Simulium asakoae* Takaoka & Davies, the first-described member of this species group, is a natural vector of a filarial species in Thailand (Fukuda et al. 2003).

The number of species in this species group in Thailand is expected to be much greater because a high rate of radiation in this species group was suggested by Jitklang et al. (2008), who described one new species (*S. doisaketense* Jitklang et al.) and recorded four unnamed species (S. sp. nr. asakoae-2, S. sp. nr. asakoae-3, S. sp. nr. asakoae-4, and S. sp. nr. sheilae-3) based on larval salivary gland chromosome studies. All but S. sp. nr. asakoae-4 are likely to be members of the *S. asakoae* species group, although their adult female and male are unknown. Similarly, Jomkumsing et al. (2019), based on an analysis of COI gene sequences, divided human-biting females of the *S. asakoae* species group collected in Thailand into seven groups, of which five groups included known and unknown haplotypes and two other groups included only unknown haplotypes, suggesting that more described species known in neighboring countries (e.g., Myanmar and Peninsular Malaysia) and more undescribed cryptic species are distributed in Thailand (pers. obs.).

The *S. asakoae* species group is separated from the other species groups in the subgenus Gomphostilbia by a combination of the yellowish hair tuft on the base of the radial vein and yellowish forecoxae in the female and male, hind basitarsus enlarged, and ventral plate emarginated on both sides when viewed ventrally in the male (Takaoka 2012).

In general, females of most members of the *S. asakoae* species group are morphologically similar to one another and are often difficult to identify to species, although certain morphological features, such as the width ratio of the frons against the head, relative length of the sensory vesicle against the length of the third palpal segment, presence or absence of the outer mandibular teeth, relative length of the fore- and hind basitarsi against their greatest width, and relative length of the claw tooth against the claw, are used for species identification (Takaoka et al. 2013, 2014b).

The possible involvement of undescribed cryptic species, as well as the difficulty of morphological identification of the females, is a serious problem for studies of the vectorial roles in the transmission of parasites and pathogens among species of the *S. asakoae* species group.

In this study, we first aimed to explore the fauna of the *S. asakoae* species group in Thailand by morphologically examining numerous females and males reared from pupae, their associated pupal exuviae and cocoons, and mature larvae collected in various provinces in this country, and secondly to molecularly resolve the genetic relationships of Thai members of the *S. asakoae* species group by a COI gene sequence-based analysis.

## Materials and methods

### Morphological analysis

The material examined in this study consisted of females and males, their associated pupal exuviae and cocoons, and mature larvae of the *S. asakoae* species group collected in various localities in Thailand. All specimens were fixed in 80% ethanol.

Methods of morphological observation, terms of features, descriptions, and illustrations followed Takaoka (2003) and partly followed Adler et al. (2004). The morphological identification at the species level was carried out by using certain female, male, pupal and larval features, such as the relative length of the female sensory vesicle, number of male upper-eye (large) facets, presence or absence of an anterodorsal projection on the cocoon, and color pattern of the larval abdomen. Diagnostic characters of each new species are provided in the “Diagnosis” to distinguish the species from all others in the *S. asakoae* species group.

Due to close morphological similarities of all new species, the description of the first new species was fully made based on as many morphological characters as possible, whereas those of the other new species were made only for morphological characters differing from those of the first new species.

The holotypes and paratypes of the new species are deposited in the Entomology Section of the Queen Sirikit Botanic Garden, Chiang Mai province, Thailand.

### Genetic analysis

Thoraces of females and males reared from pupae, and whole bodies of mature larvae were used for genetic analysis. The procedures for DNA extraction, COI gene amplification, sequencing, and data analysis followed those of Srisuka et al. (2019). The sequences were deposited in DDBJ/EMBL/GenBank under accession numbers in Figure 26. The following COI gene sequences registered in GenBank of eleven known species of the *S. asakoae* species group were used for comparison: *S. asakoae*, *S. brinchangense* Takaoka, Sofian-Azirun & Hashim, *S. izuae* Takaoka, Sofian-Azirun & Hashim, *S. lurauense* Takaoka, Sofian-Azirun & Hashim, *S. monglaense* Takaoka, Srisuka & Saeung, *S. myanmarense* Takaoka, Srisuka & Saeung, *S. rampae* Takaoka, Srisuka & Saeung, *S. roslihashimi* Takaoka & Sofian-Azirun, *S. tanahrataense* Takaoka, Sofian-Azirun & Ya’cob, *S. sofiani* Takaoka & Hashim, and *S. udomi* Takaoka & Choochote. Those of *S. leparense* Takaoka, Sofian-Azirun & Ya’cob, *S. sheilae* Takaoka & Davies, and *S. trangense* Jitklang et al., which are members of the *S. ceylonicum* species group, were also used for reference.

### Nomenclature

This paper and the nomenclatural acts have been registered in ZooBank (www.zoobank.org), the official register of the International Commission on Zoological Nomenclature. The Life Science Identifier (LSID) numbers are noted under each of the 21 new species of black flies.

## Results

### Morphological analysis

A total of 25 species was identified morphologically, comprising two known species (*S. asakoae* and *S. chiangdaoense* Takaoka & Srisuka), one newly confirmed species (*S. myanmarense* originally described from Myanmar), one newly transferred species (*S. inthanonense* Takaoka & Suzuki), and 21 new species. *Simulium inthanonense*, one of three Thai members of the *S. ceylonicum* species group, is here transferred to the *S. asakoae* species group based on the male ventral plate, which is emarginated on both sides (Fig. 24F) when viewed ventrally, one of the key characters of the *S. asakoae* species group (Takaoka 2012).

*Simulium asakoae*, originally described from Peninsular Malaysia (Takaoka and Davies 1995), was collected from various localities in many provinces (data not shown), indicating that it is the most common species among 27 species of this species group in Thailand.

Five taxa (*S. doisaketense*, S. sp. nr. asakoae-2, S. sp. nr. asakoae-3, S. sp. nr. asakoae-4, and S. sp. nr. sheilae-3) reported based on larval salivary gland chromosome studies by Jitklang et al. (2008), which are morphologically known only as pupae and larvae, were not recovered in this study.

The possibility of the distribution in Thailand of two Myanmar species (*S. myanmarense* and *S. monglaense*) was noted by Jomkumsing et al. (2019) judging from a COI gene sequence-based analysis. In our study, the distribution of *S. myanmarense* was confirmed based on morphological evidence (and also on molecular evidence), but that of *S. monglaense* was not confirmed by morphological examination.

All 21 new species are here described, and the male of *S. inthanonense* is fully described for the first time. Keys to identify all 27 members of the *S. asakoae* species group from Thailand are given for females, males, pupae, and larvae.

### Descriptions of new species

#### Simulium (Gomphostilbia) thungchangense

Taxon classificationAnimaliaDipteraSimuliidae

Takaoka, Srisuka & Saeung sp. nov.

EDD5F914-28A8-57AC-9FFC-572664F8C6F8

http://zoobank.org/9A667B98-3B35-4F7C-9F30-097D662CC2DB

**Figure 1.**
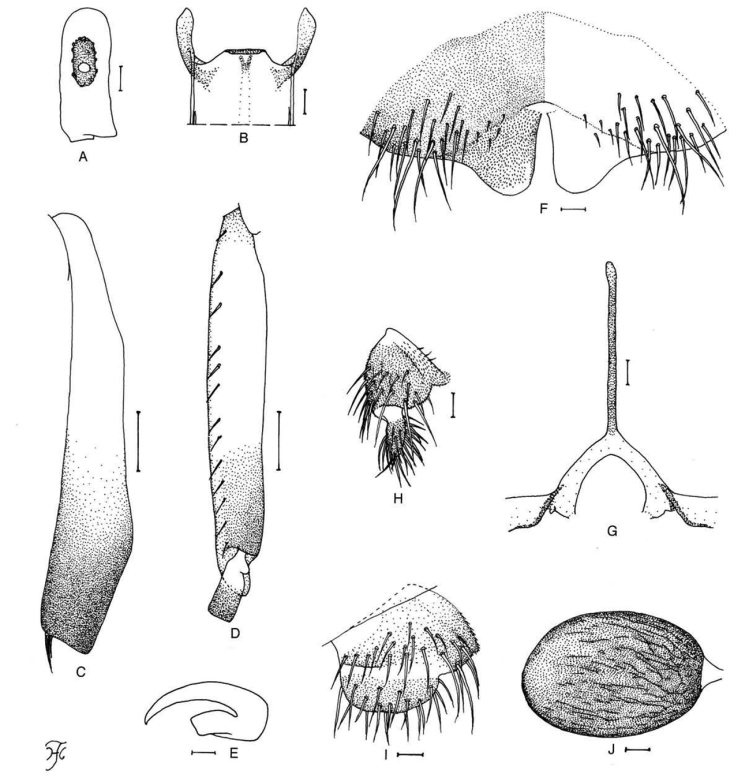
Female of *S. thungchangense* sp. nov. **A** third palpal segment with sensory vesicle (right side; anterior view) **B** cibarium (anterior view) **C** hind tibia (left side; lateral view) **D** hind basitarsus and second tarsomere (left side; lateral view) **E** claw **F** sternite 8 and ovipositor valves (ventral view) **G** genital fork (ventral view) **H, I** paraprocts and cerci (right side **H** ventral view **I** lateral view) **J** spermatheca. Scale bars: 0.1 mm (**C, D**); 0.02 mm (**A, B, F–J**); 0.01 mm (**E**).

**Figure 2.**
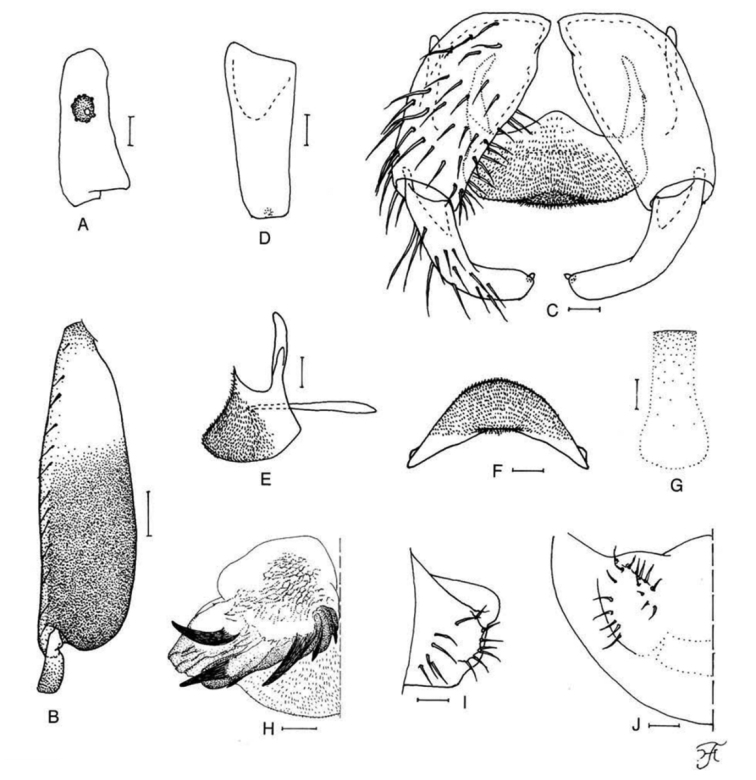
Male of *S. thungchangense* sp. nov. **A** sensory vesicle (right side; anterior view) **B** hind basitarsus and second tarsomere (left side; lateral view) **C** coxites, styles and ventral plate (ventral view) **D** style (right side; ventrolateral view) **E** ventral plate and median sclerite (lateral view) **F** ventral plate (caudal view) **G** median sclerite (caudal view) **H** paramere and aedeagal membrane (right half; caudal view) **I, J** tenth abdominal segments and cerci (right side **I** lateral view **J** caudal view). Scale bars: 0.1 mm (**B**); 0.02 mm (**A, C–J**).

**Figure 3.**
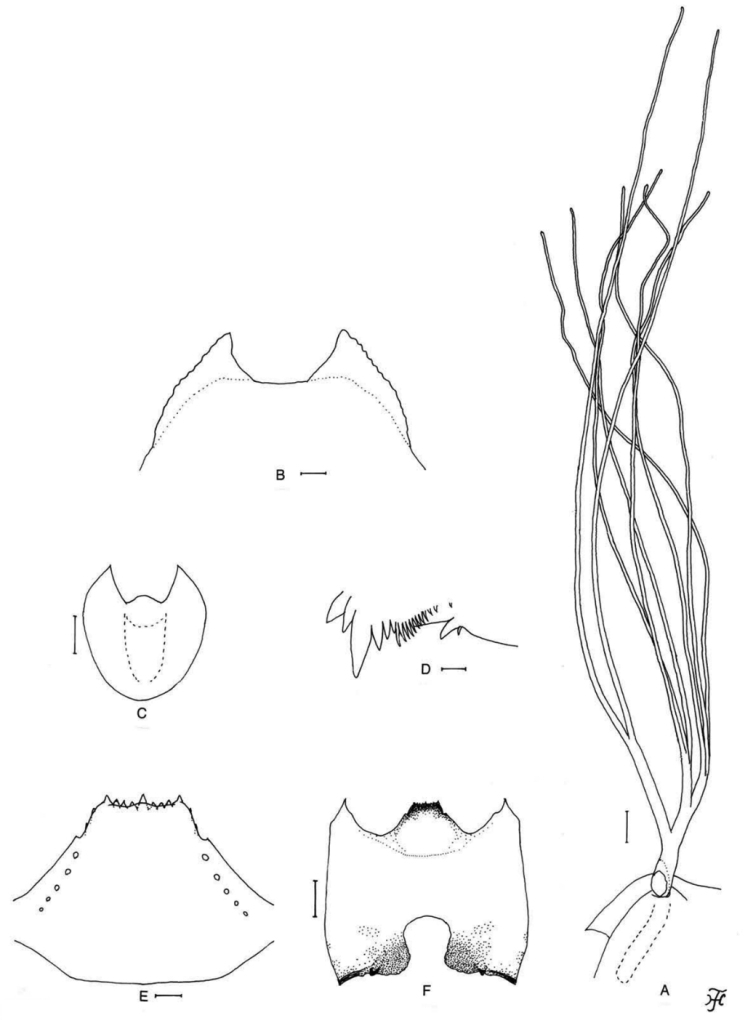
Pupa and larva of *S. thungchangense* sp. nov. **A–C** pupa **D–F** larva. **A** gill filaments (left side; lateral view) **B** terminal hooks (caudal view) **C** cocoon (dorsal view) **D** mandible **E** hypostoma **F** head capsule (ventral view). Scale bars: 1.0 mm (**C**); 0.1 mm (**A, F**); 0.02 mm (**E**); 0.01 mm for (**B, D**).

Figs 1 , 2 , 3 , 25Q

##### Material examined.

***Holotype***: Male (with its associated pupal exuviae and cocoon) (in 80% ethanol) labeled as “Holotype: *Simulium thungchangense* male, QSBG col. no. 8, Thailand, 24-I-2019, by W. Srisuka”, collected from a stream (width 60 cm, depth 10 cm, bed sandy, moderate flow, pH 6.9, 17.9 °C, exposed to the sun, elevation 1,376 m, 18°32'44.4"N, 98°30'53.0"E), at Siribhum waterfall, Chom thong District, Chiang Mai Province, Thailand, 24-I-2019, by W. Srisuka (Coll. No. 8).

***Paratypes***: 10 females, 10 males (one male for DNA analysis) (with their associated pupal exuviae and cocoons), and 20 mature larvae (two mature larvae for DNA analysis) (in 80% ethanol), same data as for holotype; one male (with its associated pupal exuviae and cocoon) and one pharate male (with its associated pupal exuviae and cocoon) (in 80% ethanol), collected from a stream (18 °C, elevation 1,470 m) from Phu Kha, Samorrophum, Thung Chang District, Nan Province, 2-XII-2004, by W. Choochote.

##### Diagnosis.

Female: mandible with several teeth on the outer margin and hind tibia yellowish on little more than the basal half (Fig. 1E). Male: small number of upper-eye facets in nine vertical columns and 12 horizontal rows, presence of many hairs on the subcosta and much widened hind basitarsus 1.5–1.7 times as wide as the hind femur. Pupa: dorsal surface of abdominal segments 1 and 2 sparsely covered with minute tubercles. Larva: postgenal cleft as long as or little longer than the postgenal bridge (Fig. 3F) and abdominal segments 1–4 light greenish or greenish grey (Fig. 25Q).

##### Description.

**Female** (*N* = 10). Body length 2.5–2.6 mm.

***Head.*** Slightly narrower than width of thorax. Frons dark brown, densely covered with yellowish white scale-like recumbent short hairs interspersed with few dark longer hairs near vertex; frontal ratio 1.8–1.9:1.0:2.5–2.6; frons:head ratio 1.0:4.2–4.7. Fronto-ocular area well developed, narrow, directed dorsolaterally. Clypeus dark brown, densely covered with yellowish white scale-like hairs interspersed with several dark longer hairs on each side. Labrum 0.62–0.68 times length of clypeus. Antenna composed of scape, pedicel and nine flagellomeres, dark brown except scape, pedicel and base of first flagellomere yellow. Maxillary palpus composed of five palpomeres, light to medium brown, proportional lengths of third, fourth, and fifth palpal segments 1.0:1.1:2.6; sensory vesicle (Fig. 1A) ellipsoidal, medium-long (0.33–0.38 times length of third palpal segment), with medium-sized opening. Lacinia with 10–12 inner and 12–16 outer teeth. Mandible with 23–26 inner teeth and five to eight outer teeth at some distance from tip. Cibarium (Fig. 1B) medially forming sclerotized plate folded forward from posterior margin, with weakly sclerotized mediolongitudinal ridge with dark bifid apex.

***Thorax.*** Scutum dark brown except anterolateral calli ochreous, and three blackish longitudinal vittae (one median, two submedian) faintly visible, thinly pruinose and shiny when illuminated at certain angles, densely covered with yellow scale-like recumbent short hairs. Scutellum medium brown, covered with yellow short hairs and dark-brown long upright hairs along posterior margin. Postnotum dark brown, slightly shiny when illuminated at certain angles, and bare. Pleural membrane ochreous and bare. Katepisternum longer than deep, medium to dark brown, shiny when illuminated at certain angles, moderately covered with fine yellow and brown short hairs.

***Legs.*** Foreleg: coxa and trochanter whitish yellow; femur dark yellow to light brown with apical cap medium brown (though extreme tip yellowish); tibia yellowish white except apical one-fourth brownish black and covered with white fine hairs on basal four-fifths; tarsus brownish black, with moderate dorsal hair crest; basitarsus moderately dilated, 6.6–6.9 times as long as its greatest width. Midleg: coxa medium brown except posterolateral surface dark brown; trochanter whitish yellow; femur light to medium brown with basal one-fourth whitish yellow and apical cap medium brown (though extreme tip yellowish); tibia whitish yellow on basal two-fifths and light to dark brown on rest (though whitish yellow on basal half or little more on posterior surface in some females), and covered with yellowish fine hairs on posterior and inner surfaces of basal two-thirds; tarsus dark brown to brownish black though basal one-third of basitarsus dark yellow (its border not well defined). Hind leg: coxa medium brown with apical one-third yellow; trochanter whitish yellow; femur medium brown with base whitish yellow and apical cap dark brown (though extreme tip yellowish white); tibia (Fig. 1C) yellowish white on basal half and light brown to brownish black on rest, covered with whitish fine hairs on outer and posterior surfaces of little more than basal three-fourths; tarsus brownish black except basal two-thirds (though base light brown) and basal half of second tarsomere yellowish white; basitarsus (Fig. 1D) narrow, nearly parallel-sided, though slightly narrowed apically, 5.9–6.4 times as long as wide, and 0.7–0.8 and 0.6 times as wide as greatest widths of tibia and femur, respectively; calcipala (Fig. 1D) nearly as long as width at base, and 0.5 times as wide as greatest width of basitarsus; pedisulcus (Fig. 1D) well developed; claw (Fig. 1E) with large basal tooth 0.5 times length of claw.

***Wing.*** Length 2.2–2.4 mm. Costa with dark spinules and hairs except basal patch of hairs yellow. Subcosta with dark hairs except near apex bare. Hair tuft on base of radius yellow. Basal portion of radius fully haired; R_1_ with dark spinules and hairs; R_2_ with hairs only. Basal cell absent.

***Halter*.** White except basal portion darkened.

***Abdomen.*** Basal scale ochreous, with fringe of whitish yellow hairs. Dorsal surface of abdomen medium to dark brown except anterior half of segment 2 ochreous, moderately covered with dark short to long hairs; tergites of segments 2 and 6–9 shiny when illuminated at certain angles. Ventral surface of segments 2–4 yellow and those of other segments medium to dark brown; sternal plate on segment 7 undeveloped.

***Terminalia.*** Sternite 8 (Fig. 1F) bare medially, with 19 or 20 medium-long to long hairs together with three or four slender short hairs on each side. Ovipositor valves (Fig. 1F) tongue-like, thin, membranous, each moderately covered with microsetae interspersed with one or two short hairs; inner margins slightly concave medially, somewhat sclerotized, and moderately separated from each other. Genital fork (Fig. 1G) of usual inverted-Y form, with slender stem; arms of moderate width, moderately folded medially. Paraproct in ventral view (Fig. 1H) somewhat concave anterolaterally, with five or six sensilla on anteromedial surface; paraproct in lateral view (Fig. 1I) somewhat produced ventrally beyond ventral tip of cercus, 0.6–0.7 times as long as wide, with 22–25 medium-long to long hairs on ventral and lateral surfaces. Cercus in lateral view (Fig. 1I) short, slightly rounded posteriorly, 0.4 times as long as wide. Spermatheca (Fig. 1J) ellipsoidal, 1.4 times as long as its greatest width, well sclerotized and darkened except duct and small area near juncture with duct unpigmented, and with many fissures on outer surface; internal setae absent; both accessory ducts slender, subequal in diameter to major one.

**Male** (*N* = 14). Body length 2.6–3.1 mm.

***Head.*** Nearly as wide as thorax. Upper eye vermilion, consisting of large facets in 9 (rarely 10) vertical columns and 12 horizontal rows on each side. Clypeus brownish black, whitish pruinose, densely covered with golden-yellow scale-like medium-long hairs (mostly directed upward) interspersed with several dark-brown simple longer hairs near lower margin. Antenna composed of scape, pedicel and nine flagellomeres, medium to dark brown except scape, pedicel, and base of first flagellomere yellow; first flagellomere elongate, 1.8 times length of second. Maxillary palpus light brown, with five palpal segments, proportional lengths of third, fourth, and fifth palpomeres 1.0:1.1–1.2:2.7–3.0; third palpomere (Fig. 2A) slender; sensory vesicle (Fig. 2A) small, ellipsoidal (0.18–0.24 times length of third palpal segment), and with small opening.

***Thorax.*** Scutum dark brown to brownish black except anterolateral calli ochreous, shiny and white pruinose when illuminated at certain angles, and densely covered with whitish yellow scale-like recumbent short hairs. Scutellum dark brown, covered with yellow short hairs and dark-brown long upright hairs along posterior margin. Postnotum brownish black, slightly shiny and white pruinose when illuminated at certain angles, and bare. Pleural membrane ochreous and bare. Katepisternum dark brown, longer than deep, shiny and white pruinose when illuminated at certain angles, moderately covered with yellow and brown fine short hairs.

***Legs.*** Foreleg: coxa whitish yellow; trochanter light brown; femur light brown except apical tip yellowish; tibia whitish yellow except little more than apical one-third dark brown, and covered with white hairs on whitish yellow portion; tarsus brownish black; basitarsus slightly dilated, 8.4–8.7 times as long as its greatest width. Midleg: coxa dark brown except posterolateral surface brownish black; trochanter dark yellow to light brown except base yellow; femur light to medium brown with base yellowish and apical cap dark brown (though apical tip yellow); tibia dark brown except basal one-third (or little more on posterior surface) whitish yellow; tarsus dark brown except basal one-fourth or less of basitarsus dark yellow to light brown (border not well defined). Hind leg: coxa dark brown; trochanter yellowish; femur medium to dark brown with base yellow and apical cap brownish black (though apical tip yellow); tibia dark brown to brownish black except little more than basal two-fifths whitish yellow; tarsus (Fig. 2B) brownish black except basal two-fifths of basitarsus and little less than basal half of second tarsomere whitish yellow; basitarsus (Fig. 2B) enlarged, 3.3–3.7 times as long as wide, and 1.1–1.2 and 1.5–1.7 times as wide as greatest width of tibia and femur, respectively; calcipala (Fig. 2B) slightly shorter than basal width, and 0.23 times as wide as greatest width of basitarsus; pedisulcus (Fig. 2B) well developed.

***Wing.*** Length 2.2–2.4 mm. Other characters as in female except subcosta with 9–13 hairs. ***Halter.*** Dull white except basal stem darkened.

***Abdomen.*** Basal scale dark brown, with fringe of light-brown hairs. Dorsal surface of abdomen medium brown to brownish black, covered with dark brown short to long hairs except segment 2 with yellowish hairs; segments 2 and 5–8 each with pair of shiny dorsolateral or lateral patches; ventral surface of segment 2 yellow, those of segments 3 and 4 yellow though sternal plates light brown, and those of other segments medium to dark brown.

***Genitalia.*** Coxite in ventral view (Fig. 2C) nearly rectangular, 1.7 times as long as its greatest width. Style in ventral view (Fig. 2C) bent inward, with triangular apex having single spine; style in ventrolateral view (Fig. 2D) slightly tapered toward apex, with truncated apex. Ventral plate in ventral view (Fig. 2C) with body transverse, 0.6 times as long as wide, with anterior margin produced anteromedially, posterior margin somewhat concave medially, and lateral margin emarginated medially, and densely covered with microsetae on ventral surface; basal arms of moderate length, slightly divergent, then convergent apically; ventral plate in lateral view (Fig. 2E) moderately produced ventrally; ventral plate in caudal view (Fig. 2F) rounded ventrally, densely covered with microsetae on posterior surface. Median sclerite (Fig. 2G) plate-like, wide. Parameres (Fig. 2H) of moderate size, each with four distinct long and medium-long stout hooks, and without minute setae on outer surface of basal arm. Aedeagal membrane (Fig. 2H) moderately setose; dorsal plate not defined. Ventral surface of abdominal segment 10 (Fig. 2I, J) slightly sclerotized along anterior margin and without distinct hairs near posterolateral corners. Cercus (Fig. 2I, J) small, rounded, with 12–17 hairs.

**Pupa** (*N* = 24). Body length 2.5–3.3 mm.

***Head.*** Integument deep yellow, moderately covered with small round tubercles except antennal sheaths and ventral surface almost bare; antennal sheath without any protuberances; frons with three pairs of unbranched long trichomes with or without coiled apices; face with pair of unbranched (rarely bifid) long trichomes with straight apices; three frontal trichomes on each side arising close together, subequal in length to one another and slightly longer than facial one.

***Thorax.*** Integument deep yellow, moderately covered with round tubercles, and with three long dorsomedial trichomes with coiled apices, two long anterolateral trichomes (anterior trichome more slender and shorter with straight or coiled apex, posterior one with coiled apex), one medium-long mediolateral trichome with straight apex, and three ventrolateral trichomes (one medium-long, two short) with straight apices, on each side; all trichomes unbranched. Gill (Fig. 3A) composed of eight slender thread-like filaments, arranged as [3+(1+2)]+2 (or rarely [(2+1)+(1+2)]+2 or [(2+1)+3]+2) from dorsal to ventral, with medium-long common basal stalk having somewhat swollen transparent basal fenestra at base; common basal stalk 0.7–0.8 times length of interspiracular trunk; dorsal and middle triplets sharing short stalk, and dorsal triplet mostly composed of three individual filaments arising at same level, middle triplet typically composed of one individual and two paired filaments with extremely short secondary stalk; stalk of ventral pair of filaments variable in length, 0.7–1.6 times length of common basal stalk, and 0.6–1.1 times length of interspiracular trunk, and 0.9 times as thick as common stalk of middle and dorsal triplets; primary stalk of dorsal triplet lying against that of lower pair at angle of 60–90° when viewed laterally; filaments of dorsal and middle triplets subequal in length (2.2–2.7 mm) and thickness to one another; two filaments of ventral pair subequal in length (3.0–3.4 mm) and thickness to each other and 1.3 times as thick as six other filaments of dorsal and middle triplets when compared basally; all filaments yellow to light brown, gradually tapered toward apex; cuticle of all filaments with well-defined annular ridges and furrows though becoming less marked apically, densely covered with minute tubercles.

***Abdomen.*** Dorsally, all segments unpigmented or light yellowish except segment 9 and bases of spine-combs of segments 6–8 yellow; segments 1 and 2 sparsely covered with minute tubercles; segment 1 with one unbranched slender short hair-like seta on each side; segment 2 with one unbranched slender short hair-like seta and five somewhat spinous minute setae submedially on each side; segments 3 and 4 each with four hooked spines and one somewhat spinous minute seta on each side; segment 5 lacking spine-combs and comb-like groups of minute spines on each side; segments 6–9 each with spine-combs in transverse row and comb-like groups of micro-spines on each side; segment 5 with four minute setae on each side; segments 6–8 each with two minute setae on each side; segment 9 with pair of wide flat terminal hooks (Fig. 3B), of which outer margin 2.7 times length of inner margin and crenulated when viewed caudally. Ventrally, segment 4 with one unbranched hook (subequal in size to those on segments 5–7) and few slender short setae, of which one is much longer and stouter, on each side; segment 5 with pair of bifid or trifid hooks submedially and few short slender setae on each side; segments 6 and 7 each with pair of bifid inner and unbranched outer hooks somewhat spaced from each other and few short slender setae on each side; segments 4–8 each with comb-like groups of micro-spines. Each side of segment 9 with three grapnel-shaped hooklets.

***Cocoon*** (Fig. 3C). Slipper-shaped, roughly to moderately woven, widely extended ventrolaterally; anterior margin thickly woven medially, often with bulge or short projection; posterior three-fifths with floor roughly woven; individual threads visible; 3.4–4.2 mm long by 2.2–3.5 mm wide.

**Mature larva** (*N* = 20). Body length 5.5–6.5 mm. Body creamy white to light ochreous with following color markings: thoracic segment 1 encircled with distinct ochreous band (though disconnected ventromedially), thoracic segments 2 and 3 ochreous on ventral surface; abdominal segments 1–4 entirely light green or greenish grey, abdominal segment 4 with reddish brown transverse band (though often partially faded, leaving narrow band or small spot(s) dorsally), abdominal segment 5 with distinct reddish brown, W-shaped, transverse band dorsally, abdominal segment 6 often with three distinct, reddish brown spots (one round dorsomedial spot and two lateral spots of various size and shape), dorsal and dorsolateral surface of abdominal segments 5–8 faintly to distinctly covered with pinkish pigment (Fig. 25Q).

***Head.*** Head capsule yellow to dark yellow except eye-spot region whitish, sparsely covered with minute setae (though moderately on dorsal surface); head spots faintly positive or indistinct. Antenna composed of three articles and apical sensillum, longer than stem of labral fan; proportional lengths of first, second, and third articles 1.0:0.8:0.7. Labral fan with 29–31 primary rays. Mandible (Fig. 3D) with three comb-teeth decreasing in length from first tooth to third; mandibular serration composed of two teeth (one medium sized, one small); major tooth at angle of little less than 90° against mandible on apical side; supernumerary serrations absent. Hypostoma (Fig. 3E) with row of nine apical teeth, of which median tooth little longer than each corner tooth; lateral margin smooth; four or five hypostomal bristles per side lying nearly parallel to lateral margin. Postgenal cleft (Fig. 3F) small, rounded, 1.0–1.2 times length of postgenal bridge. Cervical sclerites composed of pair of small yellow rod-like pieces.

***Thorax*** and ***abdomen***. Cuticle sparsely covered with unpigmented minute setae (though few posterior abdominal segments sparsely covered also with dark minute unbranched setae) dorsally; last abdominal segment densely covered with unbranched colorless minute setae on dorsolateral and lateral surfaces of each side of anal sclerite and on each lateral surface even down to base of ventral papilla. Rectal scales minute, unpigmented. Rectal organ compound, each of three lobes with seven or eight finger-like secondary lobules. Anal sclerite of usual X-form, with anterior arms 1.2 times as long as posterior ones, broadly sclerotized at base; no sensilla on broad base and posterior to posterior arms; accessory sclerite absent. Last abdominal segment with pair of large conical ventral papillae. Posterior circlet with 80–86 rows of hooklets with up to 14 or 15 hooklets per row.

##### Etymology.

The species name, *thungchangense*, refers to the district, Thung Chang, one of the two localities where this species was collected.

##### Distribution.

Thailand (Chiang Mai and Nan).

##### Discussion.

This new species is similar to *S. chaudinhense* Takaoka & Sofian-Azirun described from Vietnam (Takaoka et al. 2017a) in many characters including the presence of teeth on the outer margin of the female mandible, small number of male upper-eye facets, and pupal abdominal segments 1 and 2 each with small tubercles on the dorsal surface. However, it is distinguished from the latter species by the male hind basitarsus 1.5–1.7 times as wide as the hind femur, and larval abdominal segments 1–4 light green or greenish grey (Fig. 25Q) (in *S. chaudinhense*, the male hind basitarsus is 1.2–1.3 times as wide as the hind femur and larval abdominal segments 1 and 2 are greyish).

#### Simulium (Gomphostilbia) puaense

Taxon classificationAnimaliaDipteraSimuliidae

Takaoka, Srisuka & Saeung sp. nov.

EC61A600-92A2-5683-AC9B-30DAC5495EF5

http://zoobank.org/9B827DD8-0B95-41C4-B6AF-D5C403A7810C

Fig. 4

##### Material examined.

***Holotype***: Male (together with its associated pupal exuviae and cocoon) (in 80% ethanol) labeled as “Holotype: *Simulium puaense* male, QSBG col. no. 60, Thailand, 25-VII-2017, by W. Srisuka” reared from a pupa collected from a small waterfall (width 80 cm, depth 3 cm, flast flow, pH 7.2, 20.1 °C, exposed to the sun, elevation 1,157 m, 19°11'10.3"N, 101°04'41.7"E), Nam Dan Village, Pua District, Nan Province, northern Thailand, 25-VII-2017, by W. Srisuka (Coll. No.60).

***Paratypes***: One female (thorax for DNA analysis) (together with its associated pupal exuviae and cocoon) (in 80% ethanol), reared from a pupa collected from a stream (width 30 cm, depth 5 cm, bed sandy, moderate running, pH 6.3, 22.6 °C, exposed to the sun, elevation 1,097 m, 18°50'03.7"N, 99°22'32.2"E), at Pa Meing Village, Muang Pan District, Lampang Province, northern Thailand, 9-VIII-2016, by W. Srisuka (Coll. No. 86); three males (thorax of one male for DNA analysis) (together with their associated pupal exuviae and cocoons) (in 80% ethanol), same data as for holotype.

##### Diagnosis.

**Figure 4.**
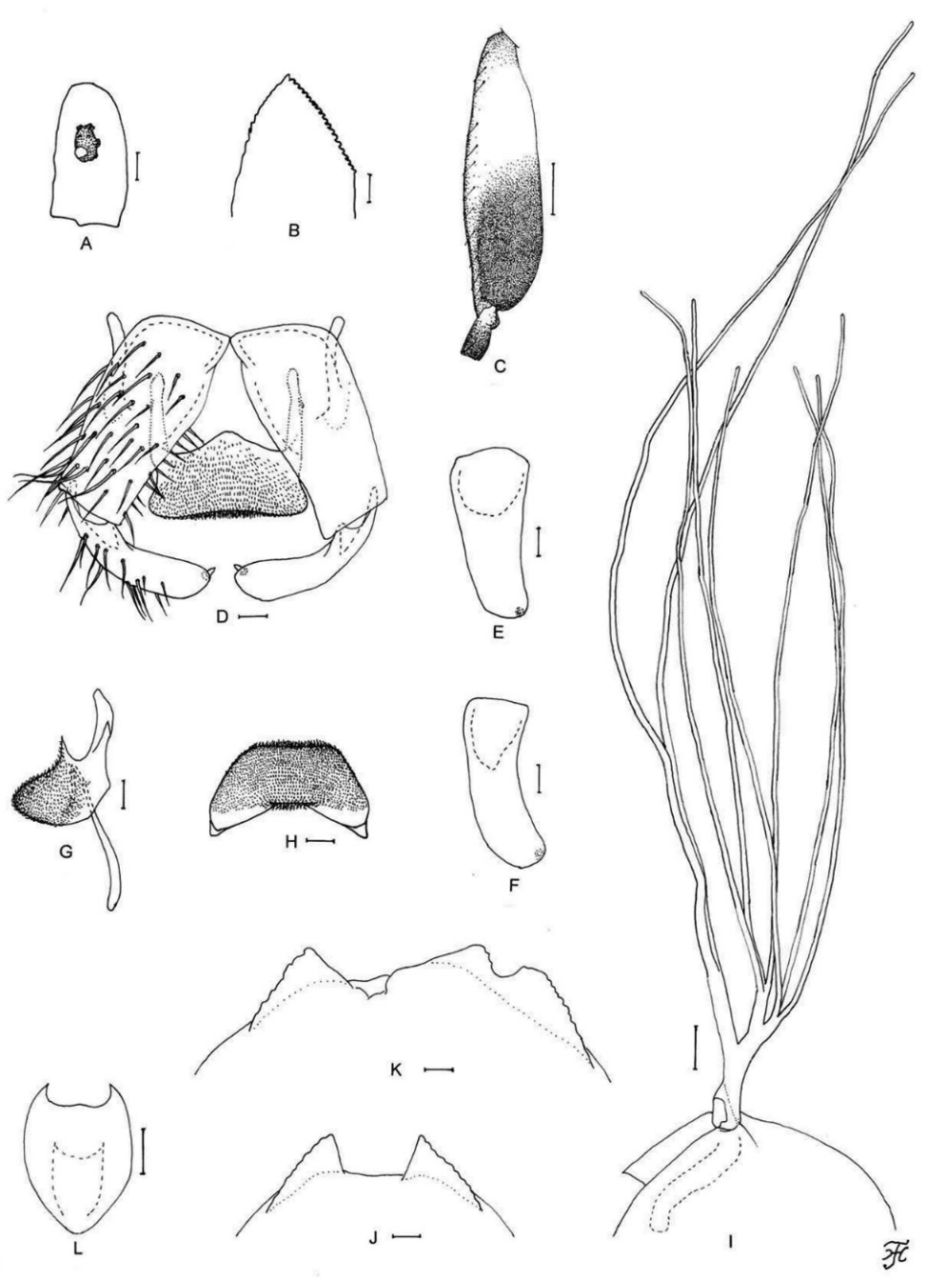
Female, male and pupa of *S. puaense* sp. nov. **A, B** female **C–H** male **I–L** pupa. **A** sensory vesicle (left side; anterior view) **B** mandible (left side) **C** hind basitarsus and second tarsomere (left side; lateral view) **D** coxites, styles and ventral plate (ventral view) **E, F** styles (right side; ventrolateral view **E** gradually tapered toward apex **F** nearly parallel-sided) **G** ventral plate and median sclerite (lateral view) **H** ventral plate (caudal view) **I** gill filaments (left side; lateral view) **J, K** terminal hooks (caudal view **J** normal **K** right hook abnormal) **L** cocoon (dorsal view). Scale bars: 1.0 mm (**L**); 0.1 mm (**C, I**); 0.02 mm (**A, D–H**); 0.01 mm (**B, J, K**).

Female: small sensory vesicle 0.24 times as long as the third palpal segment (Fig. 4A) and relatively shorter labrum against the clypeus. Male: small number of upper-eye (large) facets in nine or ten vertical columns and 12 horizontal rows, and hind basitarsus 3.2–3.6 times as long as its greatest width and 0.9–1.1 and 1.1–1.2 times as wide as the hind tibia and femur, respectively.

##### Description.

**Female** (*N* = 1). Body length 2.1 mm.

***Head.*** Frontal ratio 1.9:1.0:2.4 frons:head ratio 1.0:4.4. Labrum 0.56 times length of clypeus. Maxillary palpus: proportional lengths of third, fourth, and fifth palpal segments 1.0:1.1:2.0; sensory vesicle (Fig. 4A) 0.24 times length of third palpal segment. Lacinia with ten or eleven inner and 12 or 13 outer teeth. Mandible (Fig. 4B) with 20 inner teeth and two or three outer teeth at some distance from tip.

***Legs.*** Foreleg: coxa and trochanter whitish yellow; femur dark yellow to light brown with apical cap medium brown (though extreme tip yellowish); tibia yellowish white except little more than apical one-fourth brownish black; basitarsus moderately dilated, 5.8 times as long as its greatest width. Midleg: tarsus dark brown to brownish black though basal half of basitarsus dark yellow (its border not well defined). Hind leg: coxa light brown; tibia yellowish white on little more than basal half and light brown to brownish black on rest; basitarsus 5.9 times as long as wide, and 0.7 and 0.5 times as wide as greatest widths of tibia and femur, respectively; claw with large basal tooth 0.47 times length of claw.

***Wing.*** Length 2.0 mm.

***Abdomen.*** Dorsal surface of abdomen medium to dark brown except anterior two-thirds of segment 2 ochreous.

***Terminalia.*** Sternite 8 with 20 medium-long to long hairs together with two or three slender short hairs on each side. Paraproct in ventral view with four or five sensilla on anteromedial surface; paraproct in lateral view 0.6 times as long as wide, with 21–23 medium-long to long hairs on ventral and lateral surfaces.

**Male** (*N* = 4). Body length 2.1–2.4 mm.

***Head.*** Slightly wider than thorax. Upper eye dark brown, consisting of large facets in nine or ten vertical columns and 12 horizontal rows on each side. Antenna: first flagellomere 1.5–1.6 times length of second. Maxillary palpus light brown, with five palpal segments, proportional lengths of third, fourth, and fifth palpal segments 1.0:1.0:2.0; sensory vesicle 0.13–0.18 times length of third palpal segment.

***Legs.*** Foreleg: basitarsus moderately dilated, 6.6–7.2 times as long as its greatest width. Hind leg: tibia dark brown to brownish black except basal half whitish yellow; tarsus (Fig. 4C) brownish black except basal half or little less of basitarsus and basal one-third of second tarsomere whitish yellow; basitarsus (Fig. 4C) enlarged, 3.2–3.6 times as long as wide, and 0.9–1.1 and 1.0–1.2 times as wide as greatest width of tibia and femur, respectively; calcipala (Fig. 4C) slightly shorter than basal width, and 0.28 times as wide as greatest width of basitarsus.

***Wing*.** Length 2.0–2.1 mm. Subcosta with two to five hairs (though subcosta bare in one male).

***Genitalia.*** Coxite in ventral view (Fig. 4D) nearly rectangular, 1.7–2.2 times as long as its greatest width. Style in ventrolateral view (Fig. 4E, F) gradually tapered toward apex or nearly parallel-sided from basal one-third to apical one-fourth, with truncated apex. Ventral plate in ventral view (Fig. 4D) with basal arms of moderate length, nearly parallel-sided; ventral plate in caudal view (Fig. 4H) with ventral margin nearly straight. Cercus with 11–14 hairs.

**Pupa** (*N* = 5). Body length 2.6–3.0 mm.

***Thorax.*** Gill (Fig. 4I) composed of eight slender thread-like filaments, arranged as [3+(1+2)]+2 from dorsal to ventral; common basal stalk 0.6–0.7 times length of interspiracular trunk; dorsal and middle triplets sharing short stalk, and dorsal triplet mostly composed of three individual filaments arising at same level from extremely short stalk, middle triplet mostly composed of one individual and two paired filaments with extremely short secondary stalk; stalk of ventral pair of filaments variable in length, 0.8–1.3 times length of common basal stalk, and 0.6–0.8 times length of interspiracular trunk; primary stalk of dorsal triplet lying against that of lower pair at angle of 40–60° when viewed laterally; filaments of dorsal triplet subequal in length (1.5–1.8 mm) and thickness to one another; filaments of middle triplet subequal in length (1.7–2.0 mm); two filaments of ventral pair subequal in length (2.3–2.6 mm) and thickness to each other and 1.5 times as thick as six other filaments of dorsal and middle triplets when compared basally; all filaments light brown.

***Abdomen.*** Dorsally, all segments light yellowish; segments 1 and 2 without minute tubercles; segment 9 with pair of wide flat terminal hooks (Fig. 4J), of which outer margin is 2.0 times length of inner margin and crenulated when viewed caudally (terminal hooks abnormally formed in one pupa, Fig. 4K).

***Cocoon*** (Fig. 4L). Slipper-shaped, light yellow, moderately woven, moderately extended ventrolaterally; anterior margin thickly woven medially, rarely with small bulge; individual threads not visible; 3.0–3.5 mm long by 2.2–2.5 mm wide.

**Mature larva.** Unknown.

##### Etymology.

The species name, *puaense*, refers to the district, Pua, one of the two localities where this species was collected.

##### Distribution.

Thailand (Lampang and Nan)

##### Discussion.

This new species is similar to *S. vinhphucense* Takaoka & Low from Vietnam (Takaoka et al. 2017a) by having a small number of brown male upper-eye (large) facets, but is distinguished from the latter species by the relative length of the male fore basitarsus against its greatest width (6.6–7.2 in this new species versus 8.2 in *S. vinhphucense*) and relative width of the male hind basitarsus against the hind femur, which is 1.1–1.2 in this new species versus 1.5 in *S. vinhphucense*), and ventral plate in caudal view trapezoidal (Fig. 4H) (rounded ventrally in *S. vinhphucense*) .

This new species is also similar to *S. thungchangense* sp. nov. and *S. chaudinhense* from Vietnam (Takaoka et al. 2017a) in having a small number of male upper-eye (large) facets, but is barely distinguished from the two latter species by the upper-eye (large) facets medium brown (vermilion in the two latter species) and dorsum of pupal abdominal segments 1 and 2 bare (with minute tubercles in in the two latter species).

#### Simulium (Gomphostilbia) sutheppuiense

Taxon classificationAnimaliaDipteraSimuliidae

Takaoka, Srisuka & Saeung sp. nov.

AA667F48-F228-5606-AB37-885A6800ADF7

http://zoobank.org/10B68AD2-5E0C-4E3C-9E9F-040769084BE6

Figs 5 , 25C

##### Material examined.

***Holotype***: Male (with its associated pupal exuviae and cocoon) (in 80% ethanol) labeled as “Holotype: *Simulium sutheppuiense* male, QSBG col. no. 92, Thailand, 7-IX-2017, by W. Srisuka”, collected from a small stream (width 60 cm, depth 15 cm, bed sandy, moderate flow, pH 6.9, 20 °C, partially shaded, elevation 1,395 m, 18°49'09.5"N, 98°53'14.3"E), at Doi Pui Temple, Doi Suthep Pui, Muang District, Chiang Mai Province, Thailand, 7-IX-2017, by W. Srisuka (Coll. No. 92).

***Paratypes***: One female (thorax for DNA analysis), three males (thorax of one male for DNA analysis) (with their associated pupal exuviae and cocoons), and five mature larvae (one mature larva for DNA analysis) (in 80% ethanol), same data as for holotype;

##### Diagnosis.

**Figure 5.**
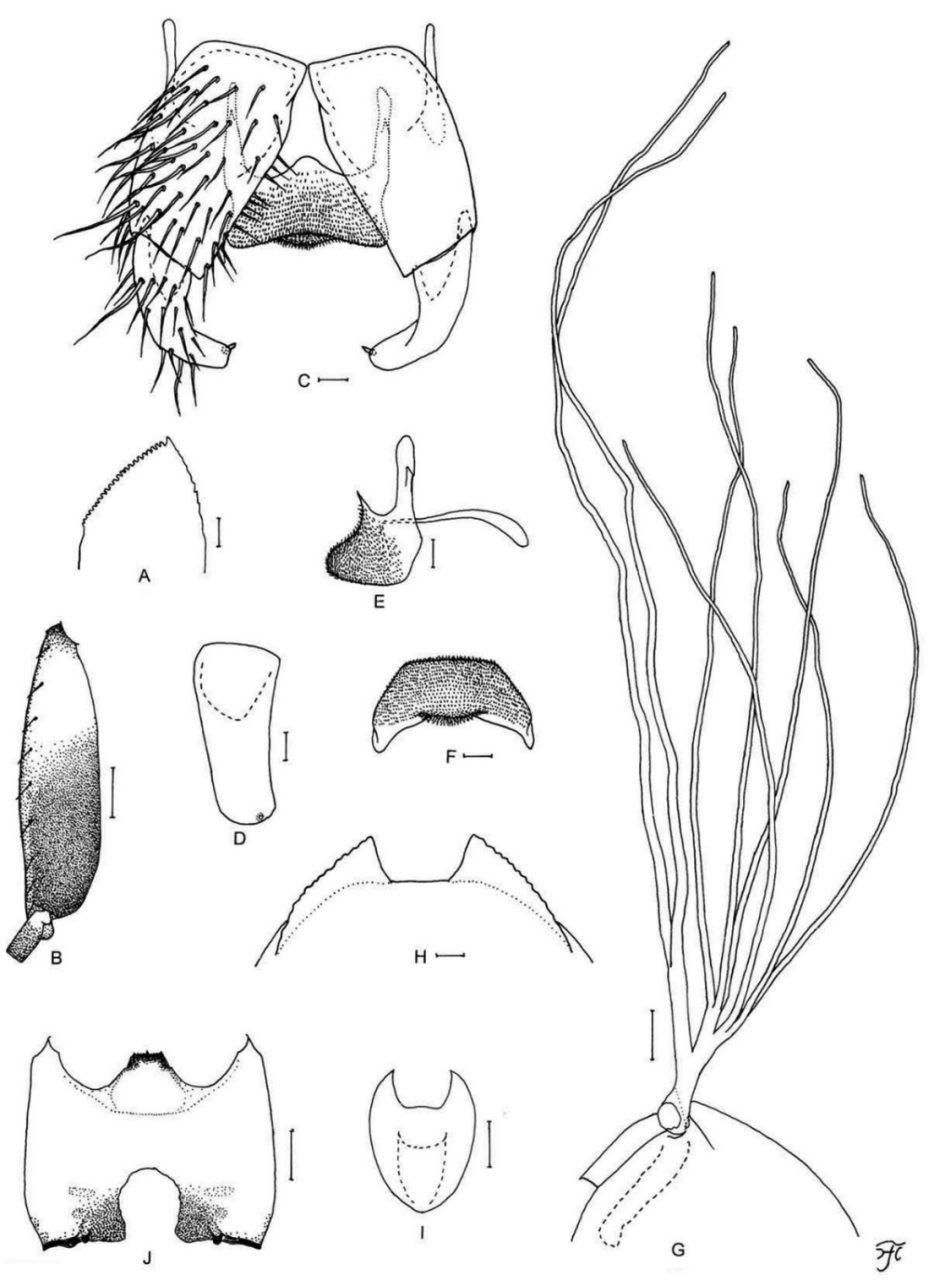
Female, male, pupa and larva of *S. sutheppuiense* sp. nov. **A** female **B–F** male **G–I** pupa **J** larva. **A** mandible (right side) **B** hind basitarsus and second tarsomere (left side; lateral view) **C** coxites, styles and ventral plate (ventral view) **D** style (right side; ventrolateral view) **E** ventral plate and median sclerite (lateral view) **F** ventral plate (caudal view) **G** gill filaments (left side; lateral view) **H** terminal hooks (caudal view) **I** cocoon (dorsal view) **J** head capsule (ventral view). Scale bars: 1.0 mm (**I**); 0.1 mm (**B, G, J**); 0.02 mm (**C–F**); 0.01 mm (**A, H**).

Female: mandible with three teeth on the outer margin (Fig. 5A). Male: small number of upper-eye facets in eleven vertical columns and 13 horizontal rows. Pupa: dorsal triplet of the gill filaments with an extremely short stalk (Fig. 5G). Larva: postgenal cleft 1.2–2.4 as long as the postgenal bridge (Fig. 5J) and abdominal segments 1–4 light ochreous (Fig. 25C).

##### Description.

**Female** (*N* = 1). Body length 2.0 mm.

***Head.*** Frontal ratio 2.0:1.0:2.5; frons:head ratio 1.0:4.6. Labrum 0.57 times length of clypeus. Maxillary palpus: proportional lengths of third, fourth, and fifth palpal segments 1.0:1.2:2.7; sensory vesicle 0.29–0.32 times length of third palpal segment. Lacinia with ten or eleven inner and 13 or 14 outer teeth. Mandible (Fig. 5A) with 23 inner teeth and three outer teeth at some distance from tip.

***Legs.*** Foreleg: basitarsus moderately dilated, 6.3 times as long as its greatest width. Midleg: tarsus dark brown to brownish black though basal half of basitarsus yellow (its border not well defined). Hind leg: coxa light brown; tibia yellowish white on basal three-fifths and light brown to brownish black on rest; basitarsus 5.3 times as long as wide, and 0.8 and 0.6 times as wide as greatest widths of tibia and femur, respectively.

***Wing.*** Length 2.0 mm.

***Abdomen.*** Dorsal surface of abdomen medium to dark brown except most of segment 2 ochreous.

***Terminalia.*** Sternite 8 with 25 or 26 medium-long to long hairs together with three or four slender short hairs on each side. Ovipositor valves each moderately covered with microsetae interspersed with two or three short hairs. Paraproct in ventral view with three or four sensilla on anteromedial surface; paraproct in lateral view 0.5 times as long as wide, and with 24 medium-long to long hairs on ventral and lateral surfaces. Cercus in lateral view 0.5 times as long as wide. Spermatheca 1.5 times as long as its greatest width.

**Male** (*N* = 4). Body length 2.0–2.2 mm.

***Head.*** Slightly wider than thorax. Upper eye dark brown, consisting of large facets in eleven vertical columns and 13 horizontal rows. Antenna light to medium brown except scape, pedicel, and base of first flagellomere yellow; first flagellomere elongate, 1.8–1.9 times length of second one. Maxillary palpus: proportional lengths of third, fourth, and fifth palpal segments 1.0:1.1:2.6; sensory vesicle 0.15 times length of third palpal segment.

***Legs.*** Foreleg: tibia whitish yellow except apical three-tenths dark brown; basitarsus slightly dilated, 7.5–7.9 times as long as its greatest width. Hind leg: coxa medium brown; tibia dark brown to brownish black except little less than basal half whitish yellow; tarsus (Fig. 5B) brownish black except basal two-fifths of basitarsus and basal one-third of second tarsomere whitish yellow; basitarsus (Fig. 5B) enlarged, 3.6–3.8 times as long as wide, and 1.0 and 1.1 times as wide as greatest width of tibia and femur, respectively; calcipala (Fig. 5B) 0.3 times as wide as greatest width of basitarsus.

***Wing.*** Length 1.9–2.0 mm. Subcosta with 1–11 hairs (though no hairs in one male).

***Genitalia.*** Coxite in ventral view (Fig. 5C) nearly rectangular, 1.8 times as long as its greatest width. Style in ventral view (Fig. 5C) with round or truncate apex; style in ventrolateral view (Fig. 5D) slightly tapered toward apex, with truncated apex, and 0.8 times as long as coxite. Ventral plate in caudal view (Fig. 5F) with ventral margin nearly straight. Cercus with 17 or 18 hairs.

**Pupa** (*N* = 5). Body length 2.5–2.7 mm.

***Head.*** Integument yellow.

***Thorax.*** Integument yellow, moderately covered with round tubercles except dorsolateral surface of posterior half sparsely covered with tubercles. Gill (Fig. 5G) composed of eight slender thread-like filaments, arranged as (3+3)+2 or [3+(1+2)]+2 from dorsal to ventral; common basal stalk 0.6–0.7 times length of interspiracular trunk; dorsal and middle triplets sharing short stalk, and each composed of three individual filaments arising at same level except middle triplet rarely composed of one individual and two paired filaments with extremely short secondary stalk; stalk of ventral pair of filaments 1.0–1.3 times length of common basal stalk, and 0.6–0.8 times length of interspiracular trunk; primary stalk of dorsal triplet lying against that of lower pair at angle of 40–70° when viewed laterally; filaments of dorsal and middle triplets subequal in length (1.5–1.8 mm) and thickness to one another; two filaments of ventral pair subequal in length (2.3–2.5 mm) and thickness to each other and 1.3–1.5 times as thick as six other filaments of dorsal and middle triplets when compared basally.

***Abdomen.*** Dorsally, all segments unpigmented except segments 1, 2, and 9 light yellowish; segments 1 and 2 without minute tubercles; segment 9 with pair of wide flat terminal hooks (Fig. 5H), of which outer margin 2.8–2.9 times length of inner margin and crenulated when viewed caudally.

***Cocoon*** (Fig. 5I). Light yellow to light brown, slipper-shaped, moderately woven, widely extended ventrolaterally; anterior margin not thickly woven medially, without bulge or short projection; individual threads visible; 3.2–3.6 mm long by 1.9–2.5 mm wide.

**Mature larva** (*N* = 4). Body length 4.7–5.5 mm. Body light ochreous with following color markings: thoracic segment 1 encircled with light to dark brown band (though disconnected ventrally), thoracic segments 2 and 3 ochreous on ventral surface; abdominal segment 4 faintly with reddish brown transverse band (though often entirely faded), abdominal segments 5 and 6 each with distinct reddish brown, W-shaped, transverse band dorsally along posterior margin, though often partially faded leaving one round dorsomedial spot and two lateral spots of various size and shape, dorsal and dorsolateral surface of abdominal segments 5–8 faintly to moderately covered with pinkish or reddish brown pigment (Fig. 25C).

***Head.*** Head capsule yellow except eye-spot region whitish; head spots moderately positive or indistinct. Antenna: proportional lengths of first, second, and third articles 1.0:0.7–0.8:0.8. Labral fan with 29 or 30 primary rays. Postgenal cleft (Fig. 5J) small to medium sized, rounded or arrow-headed, 1.2–2.4 times length of postgenal bridge.

***Thorax*** and ***Abdomen.*** Thoracic and abdominal cuticle sparsely covered with unpigmented minute setae, though few posterior abdominal segments sparsely covered with dark minute unbranched setae dorsally. Rectal organ compound, each of three lobes with nine or ten finger-like secondary lobules. Posterior circlet with 86–89 rows of hooklets with up to 14 or 15 hooklets per row.

##### Etymology.

The species name, *sutheppuiense*, refers to the locality name, Doi Suthep Pui, where this species was collected.

##### Distribution.

Thailand (Chiang Mai).

##### Discussion.

This new species is similar to *S. asakoae* in many characters including the presence of teeth on the outer margin of the female mandible, and small number of male upper-eye facets. However, it is distinguished from the latter species in the female by the shorter sensory vesicle relative to the third palpal segment, in the male by the dark brown upper-eye (large) facets, in the pupa by the light yellow dorsum of abdominal segments 1–3, and in the larva by abdominal segments 1–4 light ochreous (Fig. 25C).

#### Simulium (Gomphostilbia) teerachanense

Taxon classificationAnimaliaDipteraSimuliidae

Takaoka, Srisuka & Fukuda sp. nov.

1AFB148B-AE5C-5648-9B34-71D6A36C21D6

http://zoobank.org/40239302-BA1E-4A02-A968-0C4AA624FF15

Figs 6 , 25A

##### Material examined.

***Holotype***: Male (with its associated pupal exuviae and cocoon) (in 80% ethanol) labeled as “Holotype: *Simulium teerachanense* male, QSBG col. no. 105, Thailand, 14-VII-2017, by W. Srisuka”, collected from a small stream (width 25 cm, depth 2.5 cm, bed sandy, moderate flow, pH 6.8, 21.9 °C, partially shaded, elevation 974 m, 18°20'35.8"N, 98°01'23.8"E), at Tee Ra Chan Waterfall, Mae La-noi, Mae Hong Son Province, Thailand, 14-VII-2017, by W. Srisuka (Coll. No. 105).

***Paratypes***: Eight males (thoraces of two males for DNA analysis) (with their associated pupal exuviae and cocoons) and four mature larvae (in 80% ethanol), same data as for holotype. One mature larva (for DNA analysis) collected from a stream (elevation 817m, 19°11'22.0"N, 98°04'12.1"E) , Huai Hee Village, Mae Sarieng, Mae Hong Song Province, northern Thailand, 13-VII-2017, by W. Srisuka (Coll. No. 106).

##### Diagnosis.

**Figure 6.**
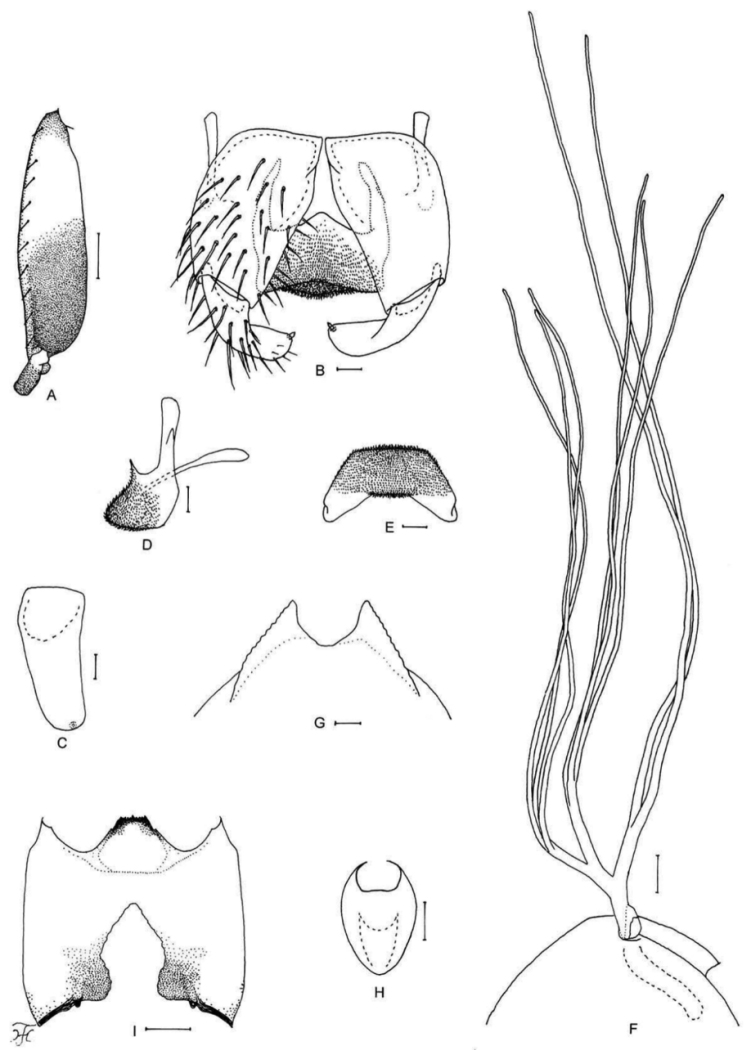
Male, pupa and larva of *S. teerachanense* sp. nov. **A–E** male **F–H** pupa **I** larva. **A** hind basitarsus and second tarsomere (left side; lateral view) **B** coxites, styles and ventral plate (ventral view) **C** style (right side; ventrolateral view) **D** ventral plate and median sclerite (lateral view) **E** ventral plate (caudal view) **F** gill filaments (right side; lateral view) **G** terminal hooks (caudal view) **H** cocoon (dorsal view) **I** head capsule (ventral view). Scale bars: 1.0 mm (**H**); 0.1 mm (**A, F, I**); 0.02 mm (**B–E**); 0.01 mm (**G**).

Male: small number of upper-eye facets in eleven vertical columns and 13 horizontal rows and antenna almost entirely yellow. Larva: postgenal cleft long, 2.8–3.3 times as long as the postgenal bridge (Fig. 6I) and abdominal segments 1–4 dull ochreous (Fig. 25A).

##### Description.

**Male** (*N* = 9). Body length 2.0–2.2 mm.

***Head.*** Somewhat wider than thorax. Upper eye dark brown, consisting of large facets in eleven vertical columns and 13 horizontal rows. Antenna entirely yellow, though few apical flagellomeres slightly darkened in some males; first flagellomere elongate, 1.8 times length of second. Maxillary palpus: proportional lengths of third, fourth, and fifth palpal segments 1.0:1.2:2.5; sensory vesicle small, globular or ellipsoidal (0.16–0.17 times length of third palpal segment).

***Legs.*** Foreleg: tibia whitish yellow except apical three-tenths dark brown; basitarsus slightly dilated, 7.3–7.5 times as long as its greatest width. Hind leg: coxa light brown; tibia dark brown to brownish black except little more than basal two-fifths whitish yellow; tarsus (Fig. 6A) brownish black except little less than basal half of basitarsus and basal one-third of second tarsomere whitish yellow; basitarsus (Fig. 6A) enlarged, wedge-shaped, 3.6–3.8 times as long as wide, and 0.9–1.0 and 1.0 times as wide as greatest width of tibia and femur, respectively; calcipala (Fig. 6A) slightly shorter than basal width, and 0.35 times as wide as greatest width of basitarsus.

***Wing.*** Length 1.9–2.0 mm. Subcosta with one to four hairs, though no hairs in three males.

***Abdomen.*** Dorsal surface of abdomen medium brown to brownish black, except most of segment 2 yellow to light ochreous.

***Genitalia.*** Ventral plate in ventral view (Fig. 6B) with basal arms nearly parallel-sided, then convergent apically; ventral plate in caudal view (Fig. 6E) with ventral margin nearly straight. Cercus with 12 or 13 hairs.

**Pupa** (*N* = 9). Body length 2.4–2.6 mm.

***Head.*** Integument yellow.

***Thorax.*** Integument yellow, moderately covered with round tubercles, except dorsolateral surface of posterior half sparsely covered with tubercles. Gill (Fig. 6F) composed of eight slender thread-like filaments, arranged as [3+(1+2)]+2 or (3+3)+2 from dorsal to ventral; common basal stalk 0.6–0.7 times length of interspiracular trunk; stalk of ventral pair of filaments variable in length, 0.8–1.3 times length of common basal stalk, and 0.5–0.8 times length of interspiracular trunk; primary stalk of dorsal triplet lying against that of lower pair at angle of 70–90° when viewed laterally; filaments of dorsal and middle triplet subequal in length (1.5–2.1 mm) and thickness to one another; two filaments of ventral pair subequal in length (2.5–3.0 mm) and thickness to each other and 1.4–1.7 times as thick as six other filaments of dorsal and middle triplets when compared basally.

***Abdomen.*** Dorsally, all segments unpigmented except segments 1, 2, and 9 and bases of spine-combs of segments 6–8 light yellow; segments 1 and 2 without minute tubercles; segment 9 with pair of wide flat terminal hooks (Fig. 6G), of which outer margin 2.6–2.9 times length of inner margin and crenulated when viewed caudally.

***Cocoon*** (Fig. 6H). Yellow to dark brown, slipper-shaped, moderately woven, extended ventrolaterally; anterior margin thickly woven medially, without bulge or short projection; individual threads visible; 2.9–3.3 mm long by 2.0–2.1 mm wide.

**Mature larva** (*N* = 4). Body length 4.5–5.5 mm. Body dull ochreous with following color markings: thoracic segment 1 encircled with dark brown band (though disconnected ventrally), abdominal segments 5 and 6 each with distinct reddish brown, W-shaped, transverse band dorsally along posterior margin, though that on segment 6 often partially faded leaving one round dorsomedial spot and two lateral spots of various size and shape), dorsal and dorsolateral surface of abdominal segments 5–8 faintly covered with pinkish or reddish brown pigments (Fig. 25A).

***Head.*** Head capsule yellow except eye-spot region whitish; head spots indistinct except those on lateral and ventral surfaces faintly positive. Antenna: proportional lengths of first, second, and third articles 1.0:0.7–0.8:0.8–1.0. Labral fan with 28–30 primary rays. Postgenal cleft (Fig. 6I) long, rounded or arrow-headed, 2.8–3.3 times length of postgenal bridge.

***Thorax*** and ***Abdomen.*** Thoracic and abdominal cuticle sparsely covered with unpigmented minute setae, though few posterior abdominal segments sparsely covered with dark minute unbranched setae dorsally. Rectal organ compound, each of three lobes with 7–11 finger-like secondary lobules. Posterior circlet with 90–95 rows of hooklets with up to 14 hooklets per row.

**Female.** Unknown.

##### Etymology.

The species name, *teerachanense*, refers to the name of the waterfall, Tee Ra Chan, where this species was collected.

##### Distribution.

Thailand (Mae Hong Son).

##### Discussion.

This new species is similar to *S. roslihashimi* described from Peninsular Malaysia (Takaoka et al. 2011b) in many characters including the small number of the male upper-eye large facets and male antenna almost entirely yellow. However, it is distinguished from the latter species by the male fore basitarsus 7.3–7.5 times as long as its greatest width (6.6–6.8 times in *S. roslihashimi*) and ventral plate with its ventral margin nearly straight (Fig. 6E) when viewed posteriorly (ventral plate rounded ventrally in *S. roslihashimi*).

#### Simulium (Gomphostilbia) maewongense

Taxon classificationAnimaliaDipteraSimuliidae

Takaoka, Srisuka & Saeung sp. nov.

07DA9195-C1BF-585A-BE8F-3D4C5B91BD08

http://zoobank.org/232C34A2-71F4-4F44-B1C9-E0E8D405219F

Figs 7 , 25M

##### Material examined.

***Holotype***: Male (with its associated pupal exuviae and cocoon) (in 80% ethanol) labeled as “Holotype: *Simulium maewongense* male, QSBG col. no. 80, Thailand, 12-VII-2018, by W. Srisuka”, collected from a small stream (width 20 cm, depth 2 cm bed sandy, slow flow, 19.5 °C, partially shaded, elevation 1,322 m, 16°06'02.9"N, 99°06'23.8"E, 98°30'53.0"E), at Chon Yen, Mae Wong National Park, Klong Lan District, Kham Phaeng Phet Province, Thailand, 12-VII-2018, by W. Srisuka (Coll. No. 80).

***Paratypes***: Three females, four males (thorax of one male for DNA analysis) (with their associated pupal exuviae and cocoons), and 14 mature larvae (two mature larvae for DNA analysis) (in 80% ethanol), same data as for holotype; one female (with its associated pupal exuviae and cocoon). (in 80% ethanol), collected from a stream of Klong Nam Lai (width 1.4 m, depth 13 cm, bed sandy, moderate flow, pH 6.23, 25.8 °C, exposed to the sun, elevation 196 m, 16°12'28.3"N, 99°15'47.8"E), at Klong Lan District, Kham Phaeng Phet Province, Thailand, 27-VI-2013, by W. Srisuka (Coll. No. 144).

##### Diagnosis.

**Figure 7.**
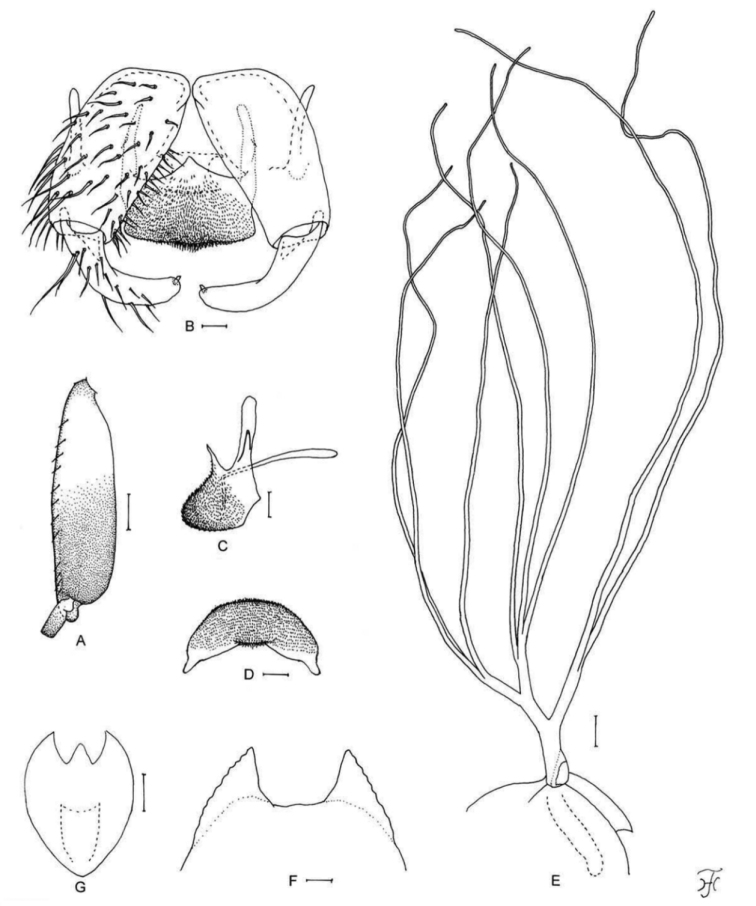
Male and pupa of *S. maewongense* sp. nov. **A–D** male **E–G** pupa. **A** hind basitarsus and second tarsomere (left side; lateral view) **B** coxites, styles and ventral plate (ventral view) **C** ventral plate and median sclerite (lateral view) **D** ventral plate (caudal view) **E** gill filaments (right side; lateral view) **F** terminal hooks (caudal view) **G** cocoon (dorsal view). Scale bars: 1.0 mm (**G**); 0.1 mm (**A, E**); 0.02 mm (**B–D**); 0.01 mm (**F**).

Female: mandible lacking teeth on the outer margin. Male: upper-eye (large) facets in eleven vertical columns and 13 or 14 horizontal rows. Pupa: thorax bare except the anterior two-fifths or half and small area of the dorsal surface near the posterior margin moderately covered with tubercles, and cocoon with a short anterodorsal projection or bulge (Fig. 7G). Larva: abdominal segments 1 and 2 greyish (Fig. 25M), and the postgenal cleft medium-long, 0.9–1.2 times as long as the postgenal bridge.

##### Description.

**Female** (*N* = 4). Body length 2.1–2.2 mm.

***Head.*** Frontal ratio 1.8–2.0:1.0:2.3–3.3. Frons: head ratio 1.0:4.3–5.4. Labrum 0.68 times length of clypeus. Maxillary palpus: proportional length of third, fourth and fifth palpal segments 1.0:1.0–1.1:2.2–2.3; sensory vesicle medium-long, 0.36 times as long as third palpal segment. Lacinia with 8–11 inner and 12 or 13 outer teeth. Mandible with 21–23 teeth on inner margin and lacking teeth on outer margin (though outer margin undulated).

***Legs.*** Fore basitarsus 6.3–6.5 times as long as its greatest width. Hind basitarsus 5.9–6.1 times as long as its greatest width and 0.7 and 0.6 times as wide as greatest width of hind tibia and femur, respectively. Calcipala nearly as long as wide and 0.56 times as wide as greatest width of hind basitarsus.

***Wing.*** Length 2.2–2.3 mm.

***Terminalia.*** Sternite 8 with 17–22 medium to long stout hairs and three to five short slender hairs. Ovipositor valve with one to four short hairs. Paraproct 0.5 times as long as wide, and with 22–25 short to medium-long hairs on outer surface. Cercus 0.4 times as long as wide. Spermatheca ellipsoidal, 1.4 times as long as its greatest width.

**Male** (*N* = 5). Body length 2.6 mm.

***Head.*** Upper eye dark brown, consisting of large facets in eleven vertical columns and 13 or 14 horizontal rows on each side. Antenna: first flagellomere 1.6 times as long as second. Maxillary palpus: proportional length of third, fourth and fifth palpal segments 1.0:1.2:2.8; sensory vesicle small, globular or ellipsoidal, 0.19–0.21 times as long as palpal segment 3.

***Legs.*** Foreleg: basitarsus 7.7 times as long as its greatest width. Hind leg: basitarsus (Fig. 7A) dark brown except little less than basal half yellowish white, and enlarged, 3.5 times as long as its greatest width, and 1.0 and 1.2–1.3 times as wide as greatest width of hind tibia and femur, respectively. Calcipala (Fig. 7A) as long as wide and 0.3 times as wide as greatest width of hind basitarsus.

***Wing.*** Length 2.2 mm. Subcosta haired except near apex bare.

***Genitalia.*** Ventral plate in ventral view (Fig. 7B) somewhat emarginated on each lateral margin; ventral plate in caudal view (Fig. 7D) rounded ventrally. Cercus with 15–17 hairs.

**Pupa** (*N* = 9). Body length 3.0 mm.

***Thorax.*** Integument almost bare except anterior two-fifths to half moderately covered with round tubercles and small dorsal area near posterior margin sparsely covered with tubercles. Gill (Fig. 7E) composed of eight slender thread-like filaments, arranged as [3+(1+2)]+2or [(2+1)+(1+2)]+2 or [(2+1)+3]+2 from dorsal to ventral, with medium-long common basal stalk having somewhat swollen transparent basal fenestra; common basal stalk 0.6–0.7 times length of interspiracular trunk; dorsal and middle triplets sharing short stalk, and dorsal triplet mostly composed of three individual filaments arising at same level, middle triplet mostly composed of one individual and two paired filaments with extremely short secondary stalk; stalk of ventral pair of filaments, 0.9–1.3 times length of common basal stalk, and 0.6–0.9 times length of interspiracular trunk, and 0.9 times as thick as common stalk of middle and dorsal triplets; primary stalk of dorsal triplet lying against that of lower pair at angle of 60–90° when viewed laterally; filaments of dorsal and middle triplets subequal in length (1.8–2.2 mm) and thickness to one another; two filaments of ventral pair subequal in length (2.8–3.2 mm) and thickness to each other and 1.6 times as thick as six other filaments of dorsal and middle triplets when compared basally.

***Abdomen.*** Dorsally, segments 1 and 2 bare. Terminal hooks (Fig. 7F) with outer margin 2.0 times as long as inner margin.

***Cocoon*** (Fig. 7G). Slipper-shaped, moderately woven, widely extended ventrolaterally; anterior margin thickly woven medially, with bulge or short projection; 3.0–4.0 mm long by 2.5–2.8 mm wide.

**Mature larva** (*N* = 12). Body length 5.5–6.5 mm. Body creamy white to light ochreous with following color markings: thoracic segment 1 encircled with ochreous or reddish brown band (though disconnected ventromedially), thoracic segments 2 and 3 ochreous on ventral surface; abdominal segments 1 and 2 entirely grey, dorsal and dorsolateral surface of abdominal segment 4 with faint reddish brown band or spot (though completely faded in some larvae), abdominal segments 5 and 6 each with reddish brown, W-shaped, transverse band near posterior margin of dorsal and dorsolateral surface, which is always distinct on abdominal segment 5 but is faded to varying extent on abdominal segment 6 leaving one small round dorsomedial spot and two distinct lateral areas, abdominal segments 7 and 8 distinctly covered with reddish brown pigment on each dorsolateral surface (Fig. 25M), and ventral surface of abdominal segments 6 and 7 each with two light reddish brown spots (though absent in some larvae).

***Head.*** Head spots faintly (or rarely moderately) positive or indistinct. Antenna: proportional lengths of first, second, and third articles 1.0:0.8:0.7–0.8. Labral fan with 27–29 primary rays. Hypostoma: median tooth little longer than each corner tooth. Postgenal cleft small, rounded, 0.9–1.2 times length of postgenal bridge.

***Abdomen.*** Rectal organ compound, each of three lobes with 6–8 finger-like secondary lobules. Anal sclerite with anterior arms 1.1–1.2 times as long as posterior ones. Posterior circlet with 91–96 rows of hooklets with up to 14 or 15 hooklets per row.

##### Etymology.

The species name, *maewongense*, refers to the name of the national park, Mae Wong, where this species was collected.

##### Distribution.

Thailand (Kham Phaeng Phet).

##### Discussion.

Among 36 species of the *S. asakoae* species group, *S. gyorkosae* Takaoka & Davies from Indonesia (Takaoka and Davies 1996), *S. jianfengense* Long et al. from Hainan Island, China (Long et al. 1994), *S. myanmarense* from Myanmar (Takaoka et al. 2017b), *S. yunnanense* Chen & Zhang from Yunnan, China (Chen and Zhang 2004), and *S. phulocense* Takaoka & Chen and *S. unii* Takaoka & Pham, both from Vietnam (Takaoka et al. 2015, 2017a), have a similar cocoon with a short anterodorsal projection. However, *S. maewongense* sp. nov. is distinguished from these six known species by the following characters (those of each related species in parentheses): from *S. gyorkosae* by the female mandible lacking outer teeth (four or five outer teeth); from *myanmarense* by the number of male upper-eye facets in eleven vertical columns (15 or rarely 14 vertical columns); from *S. jianfengense* by the ventral plate produced anteromedially when viewed ventrally (Fig. 7B) (not produced), and with its ventral margin rounded when viewed posteriorly (Fig. 7D) (pointed ventrally); from *S. yunnanense* by the yellow hair tuft of the base of the radial vein (black hair tuft); from *S. phulocense* by the relative length of the female sensory vesicle against the third palpal segment 0.36 (0.26–0.27) and male upper-eye facets in eleven vertical columns (12 or 13 vertical columns); from *S. unii* by upper-eye facets in eleven vertical columns (13 or 14 vertical columns), and the ventral plate with its lateral margins emarginated medially (Fig. 7B) (ventral plate narrowed posteriorly).

The larval body color pattern (Fig. 25M) of this new species is similar to that of *S. tuenense* Takaoka from Taiwan (Takaoka 1979), although *S. tuenense* differs from this new species by the number of male upper-eye facets in 15 vertical columns and 15 or 16 horizontal rows and cocoon without an anterodorsal projection (Huang et. al. 2011).

#### Simulium (Gomphostilbia) loeiense

Taxon classificationAnimaliaDipteraSimuliidae

Takaoka, Srisuka & Fukuda sp. nov.

D10D597F-B51C-5E37-9F8E-BAE841AC3820

http://zoobank.org/E183734D-DE58-48AA-BE1F-731ECC7B3861

Figs 8 , 25H

##### Material examined.

***Holotype***: Male (with its associated pupal exuviae and cocoon) (in 80% ethanol) labeled as “Holotype: *Simulium loeiense* male, QSBG col. no. 43, Thailand, 6-IX-2018, by W. Srisuka”, collected from a small stream (width 40 cm, depth 2 cm, bed sandy, slow flow, pH 5.8, 19.5 °C, partially shaded, elevation 1,525 m, 17°16'51.7"N, 101°31'02.5"E), at Khok Nok Kra Ba, Phu Luang, Phu Ruea District, Loei Province, Thailand, 6-IX-2018, by W. Srisuka (Coll. No. 43).

***Paratypes***: Five females, five males (thorax of one male for DNA analysis) (with their associated pupal exuviae and cocoons), and 15 mature larvae (two mature larvae for DNA analysis) (in 80% ethanol), same data as for holotype.

##### Diagnosis.

**Figure 8.**
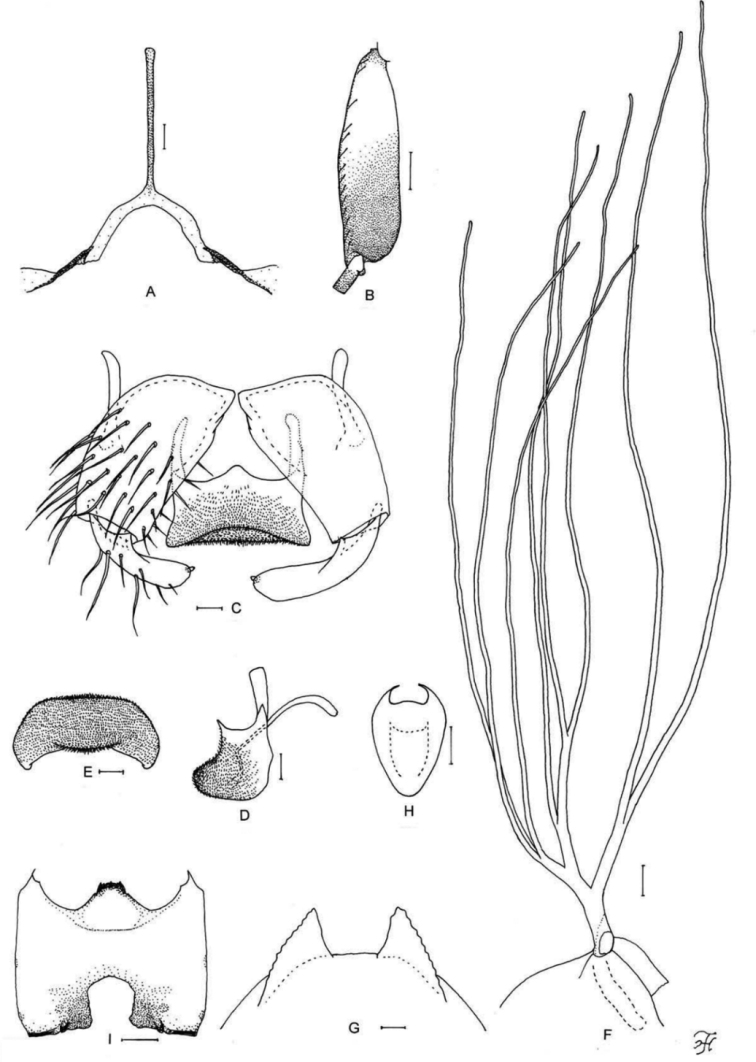
Female, male, pupa and larva of *S. loeiense* sp. nov. **A** female **B–E** male **F–H** pupa **I** larva. **A** genital fork (ventral view) **B** hind basitarsus and second tarsomere (left side; lateral view) **C** coxites, styles and ventral plate (ventral view) **D** ventral plate and median sclerite (lateral view) **E** ventral plate (caudal view) **F** gill filaments (right side; lateral view) **G** terminal hooks (caudal view) **H** cocoon (dorsal view) **I** head capsule (ventral view). Scale bars: 1.0 mm (**H**); 0.1 mm (**B, F, I**); 0.02 mm (**A, C–E**); 0.01 mm (**G**).

Female: mandible with several teeth on the outer margin. Male: small number of upper-eye facets in eleven vertical columns and 13 horizontal rows, presence of many hairs on subcosta and much widened hind basitarsus (Fig. 8B) 1.2–1.3 times as wide as the hind femur. Pupa: dorsal surface of abdominal segments 1 and 2 with several minute tubercles on each side. Larva: postgenal cleft 1.2–1.3 times as long as the postgenal bridge (Fig. 8I) and abdominal segments 1–5, 7 and 8 grey (Fig. 25H).

##### Description.

**Female** (*N* = 5). Body length 1.9–2.2 mm.

***Head.*** Frontal ratio 1.9:1.0:2.7–2.9; frons:head ratio 1.0:4.7–4.8. Labrum 0.7 times length of clypeus. Maxillary palpus: proportional lengths of third, fourth, and fifth palpal segments 1.0:1.1–1.2:2.6; sensory vesicle 0.26–0.32 times length of third palpal segment. Lacinia with 10–12 inner and 12–16 outer teeth. Mandible with 19–21 inner teeth and three to four outer teeth at some distance from tip.

***Legs.*** Foreleg: trochanter yellow to dark yellow; femur light brown with apical cap medium brown (though extreme tip yellowish); tibia white except apical one-fourth brownish black; basitarsus moderately dilated, 6.1–6.7 times as long as its greatest width. Midleg: trochanter dark yellow; femur light brown except base and extreme apical tip yellowish); tibia whitish on basal one-third, greyish on middle one-third and dark brown on apical one-third (though whitish on basal two-thirds on posterior surface); tarsus dark brown to brownish black except basal one-fourth to half yellow (its border not well defined). Hind leg: femur light to medium brown with base whitish yellow and apical cap dark brown (though extreme tip yellowish); tibia white to yellowish white on basal two-thirds or little less and brownish black on rest; tarsus brownish black except little more than basal two-thirds (though base light brown) and basal half of second tarsomere white; basitarsus 5.6–6.3 times as long as wide, and 0.7–0.8 and 0.6 times as wide as greatest widths of tibia and femur, respectively; calcipala nearly as long as width at base, and 0.53–0.57 times as wide as greatest width of basitarsus.

***Wing.*** Length 2.0–2.2 mm.

***Abdomen.*** Dorsal surface of abdomen medium to dark brown except anterior four-fifths of segment 2 ochreous.

***Terminalia.*** Sternite 8 with 21–26 medium-long to long hairs together with three or four slender short hairs on each side. Ovipositor valve moderately covered with microsetae interspersed with two to four short hairs. Genital fork (Fig. 8A) with inner margins of arms divergent apically. Paraproct with four to six sensilla on anteromedial surface; paraproct in lateral view 0.6 times as long as wide, with 23–29 medium-long to long hairs on ventral and lateral surfaces. Cercus in lateral view 0.5 times as long as wide. Spermatheca 1.3–1.4 times as long as its greatest width; both accessory ducts slender, subequal in diameter to each other and slightly thicker than major one.

**Male** (*N* = 6). Body length 2.3–2.6 mm.

***Head.*** Upper eye bright medium brown, consisting of (large) facets in eleven vertical columns and 13 horizontal rows. Antenna light to medium brown except scape, pedicel, and base of first flagellomere yellow; first flagellomere elongate, 1.5 times length of second. Maxillary palpus: proportional lengths of third, fourth, and fifth palpal segments 1.0:1.1:2.4–2.5; sensory vesicle globular or ellipsoidal (0.14–0.19 times length of third palpal segment).

***Legs.*** Foreleg: trochanter dark yellow; femur light brown except apical tip yellowish; tibia whitish except apical one-third dark brown and inner margin dark yellow to light brown, and covered with white hairs on whitish yellow portion; tarsus brownish black; basitarsus somewhat dilated, 7.1–7.6 times as long as its greatest width. Midleg: trochanter yellow; femur light brown with base yellowish and apical cap medium brown (though apical tip yellow); tibia light to medium brown except basal one-third (or little more on posterior surface) whitish; tarsus dark brown except basal one-fourth to one-third of basitarsus dark yellow (border not well defined). Hind leg: coxa light brown; trochanter yellowish; femur light to medium brown with base yellow and apical cap dark brown (though apical tip yellow); tibia medium to dark brown except little less than basal half yellowish white; tarsus (Fig. 8B) dark brown to brownish black except basal two-fifths of basitarsus (though basal half or little more of narrow portion along anterior margin) and little less than basal half of second tarsomere whitish yellow; basitarsus (Fig. 8B) 3.3–3.7 times as long as wide, and 1.0 and 1.2–1.3 times as wide as greatest width of tibia and femur, respectively; calcipala (Fig. 8B) slightly shorter than basal width, and 0.24 times as wide as greatest width of basitarsus.

***Wing.*** Length 2.0–2.1 mm. Subcosta with three to six hairs.

***Genitalia.*** Coxite in ventral view (Fig. 8C) 1.8 times as long as its greatest width. Style in ventral view (Fig. 8C) bent inward, with round apex having single spine; style in ventrolateral view slightly tapered toward apex, or nearly parallel-sided from middle to apex, with round or truncated apex, and 0.8 times length of coxite. Ventral plate in ventral view (Fig. 8C) with body transverse, 0.54 times as long as wide, with lateral margin emarginated medially; basal arms of moderate length, nearly parallel-sided, then convergent apically; ventral plate in lateral view (Fig. 8D) moderately produced ventrally; ventral plate in caudal view (Fig. 8E) trapezoidal ventrally, with ventral margin nearly straight, densely covered with microsetae on posterior surface. Cercus with 15–19 hairs.

**Pupa** (*N* = 11). Body length 2.5–2.8 mm.

***Thorax.*** Gill (Fig. 8F) composed of eight slender thread-like filaments, arranged as [(1+2)+(1+2)]+2 from dorsal to ventral; common basal stalk 0.7–0.9 times length of interspiracular trunk; primary and second stalks of dorsal triplet short, those of middle triplet mostly short (though medium-long in few pupae); stalk of ventral pair of filaments variable in length, 1.0–1.7 times length of common basal stalk, and 0.8–1.2 times length of interspiracular trunk; primary stalk of dorsal triplet lying against that of lower pair at angle of 70–80° when viewed laterally; filaments of dorsal triplet subequal in length (2.4–2.5 mm) and thickness to one another; filaments of middle triplet subequal in length (2.8–2.9 mm) and thickness to one another; two filaments of ventral pair subequal in length (3.1–3.2 mm) and thickness to each other and 1.3 times as thick as six other filaments of dorsal and middle triplets according to measurement of intact right gill of one pupa.

***Abdomen.*** Segment 9 with pair of wide flat terminal hooks (Fig. 8G), of which outer margin 2.2 times length of inner margin and crenulated when viewed caudally.

***Cocoon*** (Fig. 8H). Slipper-shaped, moderately woven, somewhat extended ventrolaterally; anterior margin thickly woven medially, often with bulge; individual threads visible or not; 2.6–3.2 mm long by 1.3–2.2 mm wide.

**Mature larva** (*N* = 13). Body length 5.0–5.5 mm. Body creamy white to light ochreous with following color markings: thoracic segment 1 encircled with distinct ochreous band (though disconnected ventromedially and dorsomedially), thoracic segments 2 and 3 ochreous or dark grey on ventral surface; abdominal segments 1–4 entirely greenish grey, abdominal segments 5, 7, and 8 dark grey on dorsal and dorsolateral surface; abdominal segments 5 and 6 each with distinct reddish brown, W-shaped, transverse band along posterior margin dorsally, though that on abdominal segment 6 often partially faded, leaving three distinct, reddish brown spots (one round dorsomedial spot and two lateral spots of various size and shape), dorsal and dorsolateral surface of abdominal segments 7 and 8 faintly to distinctly covered with reddish brown pigment (Fig. 25H); ventral surface of abdominal segments 5–7 each with pair of reddish brown small spots.

***Head.*** Head capsule yellow except narrow areas along posterior margin and surrounding areas of postgenal cleft somewhat darkened, and eye-spot region whitish; head spots distinctively positive. Antenna: proportional lengths of first, second, and third articles 1.0:0.8:0.9. Labral fan with 35 or 36 primary rays. Hypostoma: four hypostomal bristles per side lying nearly parallel to lateral margin. Postgenal cleft (Fig. 8I) medium sized, rounded, 1.3–1.4 times length of postgenal bridge.

***Abdomen.*** Rectal organ compound, each of three lobes with 10–15 finger-like secondary lobules. Anal sclerite of usual X-form, with anterior arms 0.9 times as long as posterior ones. Posterior circlet with 76–86 rows of hooklets with up to 14 or 15 hooklets per row.

##### Etymology.

The species name, *loeiense*, refers to the name of the province, Loei, where this species was collected.

##### Distribution.

Thailand (Loei).

##### Discussion.

This new species is similar to *S. asakoae* in many characters including the presence of teeth on the outer margin of the female mandible, and small number of male upper-eye facets. However, it is distinguished from the latter species by the dorsum of pupal abdominal segments 1 and 2 yellowish (the dorsum of pupal abdominal segments 1 and 2 darkened in *S. asakoae*).

#### Simulium (Gomphostilbia) maelanoiense

Taxon classificationAnimaliaDipteraSimuliidae

Takaoka, Srisuka & Saeung sp. nov.

000E73CA-E2B7-5781-B3B3-D18E00195374

http://zoobank.org/5776AAC8-0AE0-45E1-8873-B482DD48F13E

Fig. 9

##### Material examined.

***Holotype***: Male (with its associated pupal exuviae and cocoon) (in 80% ethanol) labeled as “Holotype: *Simulium maelanoiense* male, QSBG col. no. 105, Thailand, 14-VII-2017, by W. Srisuka”, collected from a small stream (width 25 cm, depth 2.5 cm, bed sandy, moderate flow, pH 6.8, 21.9 °C, partially shaded, elevation 974 m, 18°20'35.8"N, 98°01'23.8"E), at Tee La Chan Waterfall, Mae La Noi District, Mae Hong Son Province, Thailand, 14-VII-2017, by W. Srisuka (Coll. No. 105).

***Paratypes***: Two females (thoraces for DNA analysis) (with its associated pupal exuviae and cocoon) (in 80% ethanol), collected from a stream (width 110 cm, depth 12 cm, bed sandy, moderate flow, pH 6.9, 18.3 °C, partially shaded, elevation 1,446 m, 18°51'38.8"N, 99°22'15.2"E), at Kiew Fin, Muang Pan District, Lampang Province, Thailand, 6-IV-2018, by W. Srisuka (Coll. No. 36); two males (thorax of one male for DNA analysis) (with their associated pupal exuviae and cocoons) (in 80% ethanol), same data as for holotype.

##### Diagnosis.

**Figure 9.**
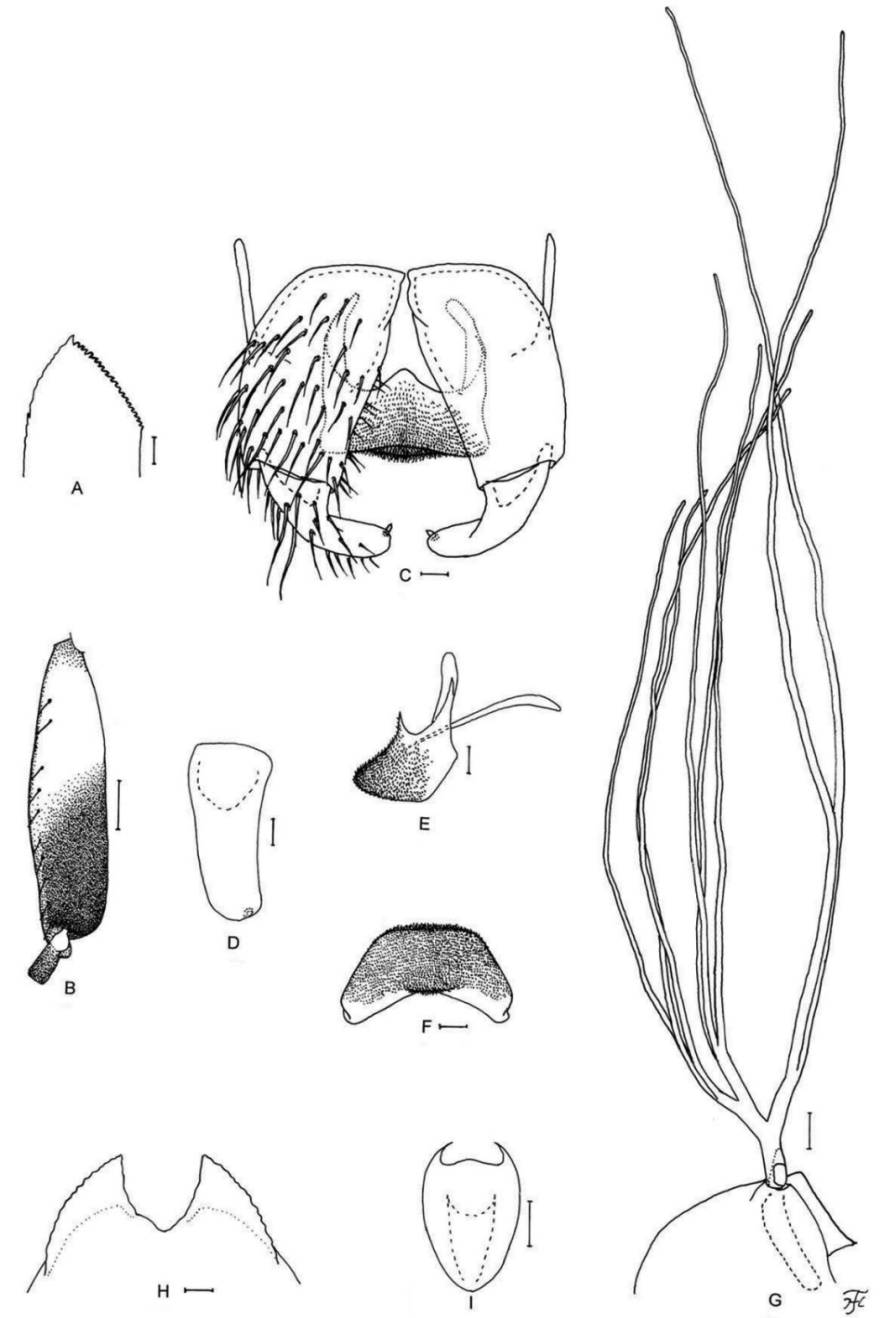
Female, male and pupa of *S. maelanoiense* sp. nov. **A** female **B–F** male **G–I** pupa. **A** mandible (left side) **B** hind basitarsus and second tarsomere (left side; lateral view) **C** coxites, styles and ventral plate (ventral view) **D** style (right side; ventrolateral view) **E** ventral plate and median sclerite (lateral view) **F** ventral plate (caudal view) **G** gill filaments (right side; lateral view) **H** terminal hooks (caudal view) **I** cocoon (dorsal view). Scale bars: 1.0 mm (**I**); 0.1 mm (**B, G**), 0.02 mm (**C–F**); 0.01 mm (**A, H**).

Female: mandible with one tooth on the outer margin (Fig. 9A). Male: small number of upper-eye facets in eleven vertical columns and 13 horizontal rows, and hind basitarsus (Fig. 9B) 1.0–1.1 times as wide as the hind femur.

##### Description.

**Female** (*N* = 2). Body length 2.0 mm.

***Head.*** Frontal ratio 1.9:1.0:2.7; frons:head ratio 1.0:4.9. Labrum 0.70 times length of clypeus. Maxillary palpus: proportional lengths of third, fourth, and fifth palpal segments 1.0:1.1:2.1; sensory vesicle 0.26–0.32 times length of third palpal segment. Lacinia with ten or eleven inner and 12 or 13 outer teeth. Mandible (Fig. 9A) with 20 inner teeth and one outer tooth at some distance from tip.

***Legs.*** Foreleg: basitarsus moderately dilated, 6.6 times as long as its greatest width. Midleg: tarsus dark brown to brownish black though basal half of basitarsus dark yellow (its border not well defined). Hind leg: coxa light brown; tibia yellowish white on basal three-fifths and light brown to brownish black on rest; tarsus brownish black except basal three-fifths (though base light brown) and basal half of second tarsomere yellowish white; basitarsus 6.9 times as long as wide, and 0.7 and 0.5 times as wide as greatest widths of tibia and femur, respectively; calcipala 0.6 times as wide as greatest width of basitarsus; claw with large basal tooth 0.44 times length of claw.

***Wing.*** Length 2.0 mm.

***Abdomen.*** Dorsal surface of abdomen medium to dark brown except anterior four-fifths of segment 2 ochreous.

***Terminalia.*** Sternite 8 with 27 medium-long to long hairs together with two to four slender short hairs on each side. Ovipositor valves each moderately covered with microsetae interspersed with one to three short hairs. Paraproct in ventral view with four sensilla on anteromedial surface; paraproct in lateral view 0.6 times as long as wide, with 19 or 20 medium-long to long hairs on ventral and lateral surfaces.

**Male** (*N* = 3). Body length 2.3–2.4 mm.

***Head.*** Somewhat wider than thorax. Upper eye medium brown, consisting of large facets in eleven (rarely ten) vertical columns and 13 horizontal rows on each side. Antenna light to medium brown except scape, pedicel, and base of first flagellomere whitish yellow; first flagellomere elongate, 1.9 times length of second. Maxillary palpus: proportional lengths of third, fourth, and fifth palpal segments 1.0:1.2:2.6; sensory vesicle 0.16–0.21 times length of third palpal segment.

***Legs.*** Foreleg: tibia yellowish white except apical three-tenths dark brown; basitarsus moderately dilated, 6.6–7.8 times as long as its greatest width. Hind leg: coxa light brown; tibia dark brown to brownish black except little less than basal half yellowish white; tarsus (Fig. 9B) brownish black except little more than basal two-fifths of basitarsus and basal one-third of second tarsomere yellowish white; basitarsus (Fig. 9B) 3.6–3.8 times as long as wide, and 1.0 and 1.0–1.1 times as wide as greatest width of tibia and femur, respectively; calcipala (Fig. 9B) slightly shorter than basal width, and 0.28 times as wide as greatest width of basitarsus.

***Wing.*** Length 2.0–2.1 mm. Subcosta with 2–13 hairs, though rarely without hair.

***Genitalia.*** Style in ventrolateral view (Fig. 9D) slightly tapered toward apex, with round apex. Ventral plate in ventral view (Fig. 9C) with basal arms of moderate length, nearly parallel-sided, then convergent apically; ventral plate in caudal view (Fig. 9F) with ventral margin nearly straight. Cercus with 13–15 hairs.

**Pupa** (*N* = 5). Body length 2.5–3.0 mm.

***Head.*** Integument yellow.

***Thorax.*** Integument yellow, moderately covered with round tubercles except dorsal surface of posterior half sparsely covered with tubercles, and dorsolateral surface of posterior half almost bare. Gill (Fig. 9G) composed of eight slender thread-like filaments, arranged as (3+3)+2 (or rarely [3+(1+2)]+2) from dorsal to ventral; common basal stalk 0.6–0.7 times length of interspiracular trunk; dorsal triplet composed of three individual filaments arising at same level, and with extremely short stalk in some pupae, middle triplet mostly composed of three individual filaments arising at same level, or rarely composed of one individual and two paired filaments with extremely short secondary stalk; stalk of ventral pair of filaments variable in length, 0.6–1.3 times length of common basal stalk, and 0.5–1.0 times length of interspiracular trunk; primary stalk of dorsal triplet lying against that of lower pair at angle of 60–80° when viewed laterally; filaments of dorsal triplet subequal in length (1.5–2.0 mm) and thickness to one another; filaments of middle triplet subequal in length (1.7–2.5 mm) and thickness, and two filaments of ventral pair subequal in length (2.5–3.6 mm) and thickness to each other and 1.4–1.8 times as thick as six other filaments of dorsal and middle triplets when compared basally.

***Abdomen.*** Dorsally, segments 1 and 2 without minute tubercles; segment 9 with pair of wide flat terminal hooks (Fig. 9H), of which outer margin 2.1–2.3 times length of inner margin and crenulated when viewed caudally.

***Cocoon*** (Fig. 9I). Dark brown, slipper-shaped, moderately woven, moderately extended ventrolaterally; anterior margin thickly woven medially, often with bulge; 3.0–3.8 mm long by 2.0–2.5 mm wide.

**Mature larva.** Unknown.

##### Etymology.

The species name, *maelanoiense*, refers to the district, Mae La Noi, where this species was collected.

##### Distribution.

Thailand (Lampang and Mae Hong Son).

##### Discussion.

*Simulium maelanoiense* sp. nov. is most similar in the male and pupa to *S. sutheppuiense* sp. nov. in many characters including the number of male upper-eye facets, relative width of the hind basitarsus compared to the hind tibia and femur, shape of the ventral plate when viewed caudally, and arrangement of the pupal gill. However, this new species is barely distinguished in the female from *S. sutheppuiense* sp. nov. by the number of the outer teeth of the mandible (one tooth in this new species versus three teeth in *S. sutheppuiense* sp. nov.) and the length ratio of the labrum against the clypeus (0.7 in this new species versus 0.6 in *S. sutheppuiense* sp. nov.).

#### Simulium (Gomphostilbia) phapeungense

Taxon classificationAnimaliaDipteraSimuliidae

Takaoka, Srisuka & Fukuda sp. nov.

29E090A6-CBD1-5523-B41C-236188BC5925

http://zoobank.org/25D1EED4-BFCB-4080-9122-9D511575114C

Figs 10 , 25N

##### Material examined.

***Holotype***: Male (thorax for DNA analysis) (with its associated pupal exuviae and cocoon) (in 80% ethanol) labeled as “Holotype: *Simulium phapeungense* male, QSBG col. no. 107, Thailand, 12-VII-2017, by W. Srisuka”, collected from a stream (width 120 cm, depth 2 cm, bed sandy, fast flow, pH 7.6, 21.2 °C, partially shade, elevation 1,034 m, 19°36'58.5"N, 97°59'48.2"E), at Pha Peung, Muang District, Mae Hong Son Province, Thailand, 12-VII-2017, by W. Srisuka (Coll. No.107).

***Paratype***: One male (thorax for DNA analysis) (with its associated pupal exuviae and cocoon) (in 80% ethanol), same data as for holotype; five mature larvae (one mature larva for DNA analysis) (in 80% ethanol), collected from a stream (width 80 cm, depth 2.5 cm, moderate flow, pH 7.2, 20.1 °C, partially shaded, elevation 1,157 m, 19°11'10.3"N, 101°04'41.7"E), Nam Dan Village, Pua District, Nan Province, Thailand, 25-VII-2017, by W. Srisuka (Coll. No. 60).

##### Diagnosis.

**Figure 10.**
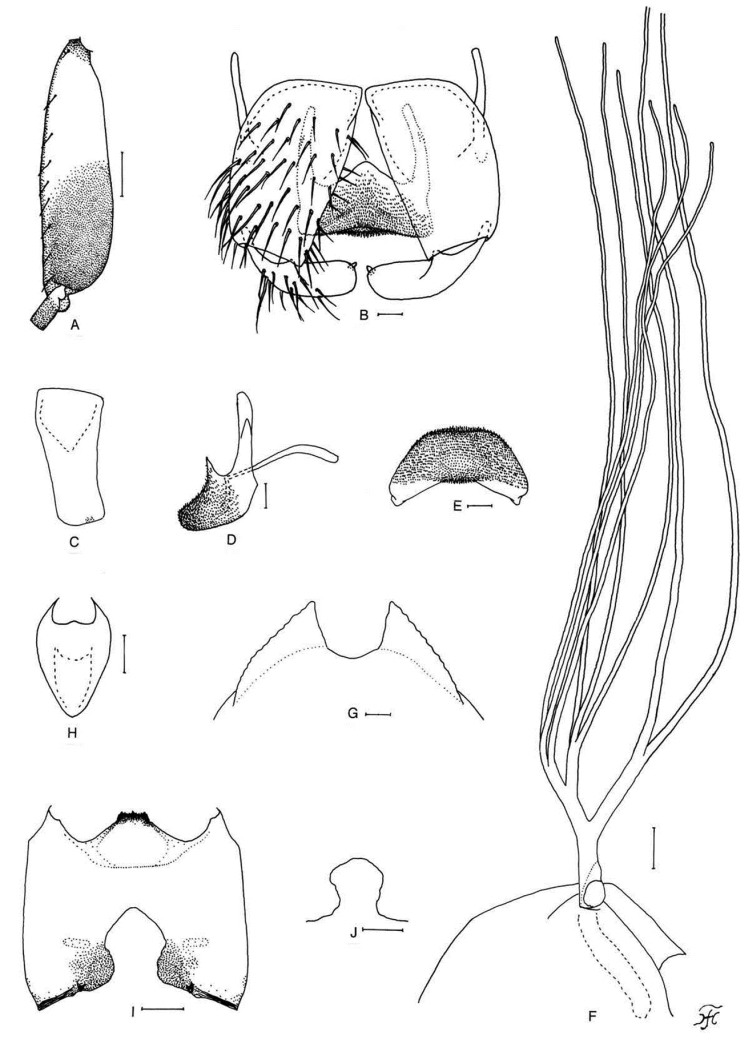
Male, pupa and larva of *S. phapeungense* sp. nov. **A–E** male **F–H** pupa **I, J** larva **A** hind basitarsus and second tarsomere (left side; lateral view) **B** coxites, styles and ventral plate (ventral view) **C** style (right side; ventrolateral view) **D** ventral plate and median sclerite (lateral view) **E** ventral plate (caudal view) **F** gill filaments (right side; lateral view) **G** terminal hooks (caudal view) **H** cocoon (dorsal view) **I** head capsule showing postgenal cleft (ventral view) **J** postgenal cleft (ventral view). Scale bars: 1.0 mm (**H**); 0.1 mm (**F, I, J**); 0.02 mm (**A–E**); 0.01 mm (**G**).

Male: small number of brown upper-eye (large) facets in eleven vertical columns and 13 horizontal rows, and widened hind basitarsus (Fig. 10A) 1.2 times as wide as the hind femur. Pupa: dorsum of pupal abdominal segments 1 and 2 without minute tubercles. Larva: abdominal segments 1, 3, 4, and 5 light grey (Fig. 25N).

##### Description.

**Male** (*N* = 2). Body length 2.2 mm.

***Head.*** Somewhat wider than thorax. Upper eye dark brown, consisting of large facets in eleven vertical columns and 13 horizontal rows on each side. Antenna: first flagellomere elongate, 1.5 times length of second. Maxillary palpus: proportional lengths of third, fourth, and fifth palpomeres 1.0:1.3:2.7; sensory vesicle ellipsoidal, 0.18–0.21 times length of third palpomere.

***Legs.*** Foreleg: tibia whitish except apical three-tenths dark brown; basitarsus somewhat dilated, 6.9 times as long as its greatest width. Midleg: tarsus dark brown except basal one-third of basitarsus dark yellow (border not well defined). Hind leg: coxa light brown; tibia light to dark brown except little less than basal half whitish; tarsus (Fig. 10A) dark brown except basal half of basitarsus and basal one-third of second tarsomere whitish to yellowish white; basitarsus (Fig. 10A) enlarged, 3.5 times as long as wide, and 1.0 and 1.2 times as wide as greatest width of tibia and femur, respectively; calcipala (Fig. 10A) 0.27 times as wide as greatest width of basitarsus.

***Wing.*** Length 2.0 mm. Subcosta with five hairs.

***Genitalia.*** Style in ventral view (Fig. 10B) bent inward, with round apex. Ventral plate in ventral view (Fig. 10B) with basal arms nearly parallel-sided, then convergent apically; ventral plate in caudal view (Fig. 10E) with ventral margin nearly straight or slightly concave. Cercus with 12 or 13 hairs.

**Pupa** (*N* = 2). Body length 2.5 mm.

***Head.*** Integument yellow.

***Thorax.*** Integument yellow, moderately covered with round tubercles except dorsal surface of posterior two-thirds sparsely covered with tubercles and dorsolateral surfaces of posterior two-thirds almost bare. Gill (Fig. 10F) composed of eight slender thread-like filaments, arranged as (3+3)+2 from dorsal to ventral; common basal stalk 0.7–0.8 times length of interspiracular trunk; dorsal and middle triplets each composed of three individual filaments arising at same level; stalk of ventral pair of filaments variable in length, 0.9–1.3 times length of common basal stalk, and 0.7–1.0 times length of interspiracular trunk; primary stalk of dorsal triplet lying against that of lower pair at angle of 50–60° when viewed laterally; filaments of dorsal triplet subequal in length (1.9–2.0 mm) and thickness to one another; filaments of middle triplet subequal in length (2.0–2.2 mm) and thickness to one another; two filaments of ventral pair, of which apical tips were lost, probably ca. 2.5 mm long and 1.3–1.8 times as thick as six other filaments of dorsal and middle triplets when compared basally.

***Abdomen.*** Dorsally, all segments light yellowish except bases of spine-combs of segments 6–8 yellow; segments 1 and 2 without minute tubercles; segment 9 with pair of wide flat terminal hooks (Fig. 10G), of which outer margin 2.5–2.6 times length of inner margin and crenulated when viewed caudally.

***Cocoon*** (Fig. 10H). Pale yellow or medium brown, slipper-shaped, roughly or moderately woven, somewhat extended ventrolaterally; anterior margin thickly woven medially, often with small bulge; individual threads visible; 2.8–3.3 mm long by 2.0 mm wide.

**Mature larva** (*N* = 4). Body length 4.8–5.2 mm. Body creamy white to light ochreous with following color markings: thoracic segment 1 encircled with distinct grey band (though disconnected ventromedially), thoracic segments 2 and 3 grey on ventral surface; abdominal segments 1, 3, 4, and 5 light grey, abdominal segment 4 with reddish brown small spots dorsolaterally in one larva, abdominal segments 5 and 6 each with distinct reddish brown, W-shaped, transverse band dorsally (though that on abdominal segment 6 partially faded, leaving one round dorsomedial spot and two lateral spots of various size and shape), dorsal and dorsolateral surface of abdominal segments 7 and 8 faintly greyish partially, overlaid by reddish brown pigment (Fig. 25N).

***Head.*** Head capsule yellow except narrow portion along posterior margin moderately darkened connected to dark posterolateral spots; head spots faintly to moderately positive. Antenna: proportional lengths of first, second, and third articles 1.0:0.8:0.8. Labral fan with 30–33 primary rays. Hypostoma: lateral margin with four to six hypostomal bristles per side. Postgenal cleft (Fig. 10I, J) rounded (in three larvae) or somewhat pointed (in two larvae), 1.0–2.0 times length of postgenal bridge.

***Abdomen.*** Rectal organ compound, each of three lobes with 11–14 finger-like secondary lobules. Posterior circlet with 85–87 rows of hooklets with up to 14 or 15 hooklets per row.

**Female.** Unknown.

##### Etymology.

The species name, *phapeungense*, refers to the name of the village, Pha Peung, where this species was collected.

##### Distribution.

Thailand (Mae Hong Son and Nan).

##### Discussion.

*Simulium phapeungense* sp. nov. is similar to *S. loeiense* sp. nov. described above by having the small number of brown upper-eye (large) facets in eleven vertical columns and 13 horizontal rows and a widened hind basitarsus (Fig. 10A) 1.2 times as wide as the hind femur. However, this new species is readily distinguished from the latter species by the dorsum of pupal abdominal segments 1 and 2 without minute tubercles and larval abdominal segments 1, 3, 4, and 5 light grey (Fig. 25N).

#### Simulium (Gomphostilbia) nanthaburiense

Taxon classificationAnimaliaDipteraSimuliidae

Takaoka, Srisuka & Fukuda sp. nov.

E7339C71-5982-5EFE-A77C-1B4AD394799F

http://zoobank.org/BDE6F4E0-EAE2-4806-AA76-016012E171A0

Fig. 11

##### Material examined.

***Holotype***: Male (with its associated pupal exuviae and cocoon) (in 80% ethanol) labeled as “Holotype: *Simulium nanthaburiense* male, QSBG col. no. 60, Thailand, 25-VII-2017, by W. Srisuka”, collected from a stream (width 80 cm, depth 2.5 cm, moderate flow, pH 7.2, 20.1 °C, partially shaded, elevation 1,157 m, 19°11'10.3"N, 101°04'41.7"E), Nam Dan Village, Pua District, Nan Province, Thailand, 25-VII-2017, by W. Srisuka (Coll. No. 60).

***Paratypes***: One female (thorax for DNA analysis) and 10 males (thorax of one male for DNA analysis) (with their associated pupal exuviae) (in 80% ethanol), same date and data as the holotype; one female (thorax for DNA analysis) (with their associated pupal exuviae) (in 80% ethanol) collected from a stream (width 120 cm, depth 10 cm, bed sandy, moderate flow, pH 6.63, 17.1 °C, partially shaded, elevation 1,154 m, 19°03'36.8"N, 99°19'15.7"E), Huai Mor Nuea Village, Doi Saket, Chiang Mai Province, northern Thailand, 2-II-2019, by W. Srisuka and A. Saeung (Coll. No. 49).

##### Diagnosis.

Female: mandible with two distinct teeth on the outer margin (11A). Male: small number of upper-eye facets in 12 vertical columns and 13 or 14 horizontal rows.

##### Description.

**Female** (*N* = 2). Body length 2.0 mm.

**Figure 11.**
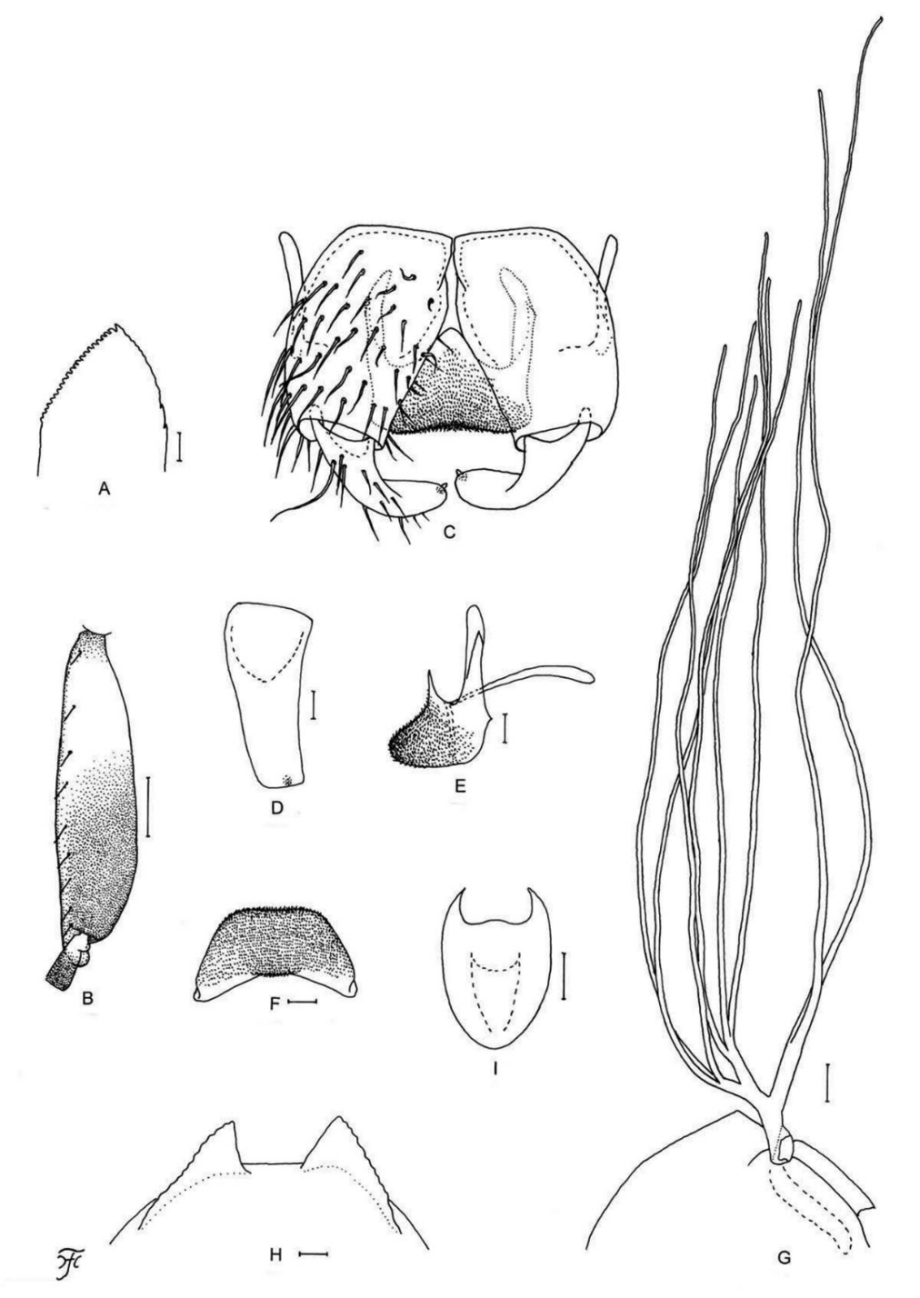
Female, male and pupa of *S. nanthaburiense* sp. nov. **A** female **B–F** male **G–I** pupa. **A** mandible (right side) **B** hind basitarsus and second tarsomere (left side; lateral view) **C** coxites, styles and ventral plate (ventral view) **D** style (right side; ventrolateral view) **E** ventral plate and median sclerite (lateral view) **F** ventral plate (caudal view) **G** gill filaments (right side; lateral view) **H** terminal hooks (caudal view) **I** cocoon (dorsal view). Scale bars: 1.0 mm (**I**); 0.1 mm (**B, G**); 0.02 mm (**C–F**); 0.01 mm (**A, H**).

***Head.*** Frontal ratio 1.7:1.0:2.1; frons:head ratio 1.0:4.3. Labrum 0.64 times length of clypeus. Maxillary palpus: proportional lengths of third, fourth, and fifth palpal segments 1.0:1.1:2.4; sensory vesicle 0.26–0.29 times length of third palpal segment. Maxillary lacinia with ten or eleven inner and 13 or 14 outer teeth. Mandible (Fig. 11A) with 22 inner teeth and two outer teeth at some distance from tip.

***Legs.*** Foreleg: coxa and trochanter whitish yellow; femur dark yellow to light brown with apical cap medium brown (though extreme tip yellowish); tibia yellowish white except little less than apical three-tenths brownish black; basitarsus moderately dilated, 6.0 times as long as its greatest width. Midleg: tarsus dark brown to brownish black though basal half or little less of basitarsus dark yellow (its border not well defined). Hind leg: coxa light brown with apical one-third yellow; tibia yellowish white on basal two-thirds and brownish black on rest; basitarsus 6.2 times as long as wide, and 0.65 and 0.53 times as wide as greatest widths of tibia and femur, respectively; calcipala slightly longer than width at base, and 0.56 times as wide as greatest width of basitarsus; claw with large basal tooth 0.45 times length of claw.

***Wing.*** Length 2.0 mm.

***Abdomen.*** Dorsal surface of abdomen medium to dark brown except most of segment 2 ochreous.

***Terminalia.*** Sternite 8 with 22 or 23 medium-long to long hairs together with three to six slender short hairs on each side. Paraproct in ventral view with three or four sensilla on anteromedial surface; paraproct in lateral view 0.6 times as long as wide, with 27–29 medium-long to long hairs on ventral and lateral surfaces. Cercus in lateral view 0.5 times as long as wide. Spermatheca 1.6 times as long as its greatest width.

**Male** (*N* = 11). Body length 2.0–2.3 mm.

***Head.*** Somewhat wider than thorax. Upper eye dark brown, consisting of large facets in 12 vertical columns and 13 or 14 horizontal rows. Antenna light to medium brown except scape, pedicel and base of first flagellomere yellowish white; first flagellomere elongate, 1.8 times length of second. Maxillary palp light brown, with five palpal segments, proportional lengths of third, fourth, and fifth palpal segments 1.0:1.1.2:2.3; sensory vesicle 0.19 times length of third palpal segment.

***Legs.*** Foreleg: trochanter yellow to dark yellow; tibia whitish except apical three-tenths dark brown and subbasal portion dark yellow to light brown (though outer surface whitish); basitarsus moderately dilated, 6.8–7.4 times as long as its greatest width. Midleg: tarsus dark brown except basal one-fourth to one-third of basitarsus dark yellow to light brown (border not well defined). Hind leg: coxa light brown; tarsus (Fig. 11B) brownish black except basal half to two-fifths of basitarsus and basal one-third of second tarsomere yellow; basitarsus (Fig. 11B) 3.8–4.1 times as long as wide, and 0.9 and 1.0 times as wide as greatest width of tibia and femur, respectively; calcipala (Fig. 11B) slightly shorter than basal width, and 0.33 times as wide as greatest width of basitarsus.

***Wing.*** Length 1.9–2.0 mm. Subcosta with 1–10 hairs, though no hair in two males.

***Genitalia.*** Coxite in ventral view (Fig. 11C) 1.6 times as long as its greatest width. Style in ventral view (Fig. 11C) with round apex. Ventral plate in caudal view (Fig. 11F) with ventral margin nearly straight. Cercus small, rounded, with 12 or 13 hairs.

**Pupa** (*N* = 13). Body length 2.5–2.7 mm.

***Head.*** Integument yellow. Thorax. Integument yellow, moderately covered with round tubercles except dorsolateral surface of posterior half sparsely covered with tubercles. Gill (Fig. 11G) composed of eight slender thread-like filaments, arranged as (3+3)+2 or [3+(1+2)]+2 or [(2+1)+(1+2)]+2 or [(2+1)+3]+2) from dorsal to ventral; common basal stalk 0.6–0.8 times length of interspiracular trunk; stalk of ventral pair of filaments short, 0.6–0.9 times length of common basal stalk, and 0.4–0.7 times length of interspiracular trunk; primary stalk of dorsal triplet lying against that of lower pair at angle of 70–90° when viewed laterally; filaments of dorsal triplet subequal in length (1.9–2.0 mm) and thickness to one another; filaments of middle triplet subequal in length (2.0–2.3 mm) and thickness to one another; two filaments of ventral pair subequal in length (2.9–3.0 mm) and thickness to each other and 1.5–1.7 times as thick as six other filaments of dorsal and middle triplets when compared basally; all filaments light brown (rarely dark brown).

***Abdomen.*** Dorsally, all segments light yellowish; segments 1 and 2 without minute tubercles; segment 9 with pair of wide flat terminal hooks (Fig. 11H), of which outer margin 1.9–2.1 times length of inner margin and crenulated when viewed caudally.

***Cocoon*** (Fig. 11I). Pale yellow, slipper-shaped, moderately woven, moderately extended ventrolaterally; anterior margin thickly woven medially, often with bulge; individual threads visible or not; 3.0–3.3 mm long by 1.8–2.5 mm wide.

**Mature larva.** Unknown.

##### Etymology.

The species name, *nanthaburiense*, refers to the historical name of Nan Province, Nanthaburi, where this species was collected.

##### Distribution.

Thailand (Nan).

##### Discussion.

This new species is similar to *S. brinchangense* described from Peninsular Malaysia (Takaoka et al. 2014b), in having a similar number of male upper-eye facets, but is distinguished by the relative length of the stalk of the ventral pair of filaments against the common basal stalk, which is 0.6–0.9 in this new species but 1.1–1.2 in *S. brinchangense*, and relative length of the outer margin against the inner margin of the pupal terminal hooks, which is 1.9–2.1 in this new species but 3.0–3.6 in *S. brinchangense*.

#### Simulium (Gomphostilbia) nanoiense

Taxon classificationAnimaliaDipteraSimuliidae

Takaoka, Srisuka & Saeung sp. nov.

8F973479-0808-5384-AB51-809228A4F6AF

http://zoobank.org/2DE34247-A149-4F4D-BF0C-AD6A8EBC6D64

Figs 12 , 25J

##### Material examined.

***Holotype***: Male (with its associated pupal exuviae and cocoon) (in 80% ethanol) labeled as “Holotype: *Simulium nanoiense* male, QSBG col. no. 66, Thailand, 5-VIII-2017, by W. Srisuka”, collected from a stream (width 1 m, depth 3 cm, bed sandy, moderate flow, pH 6.2, 19.6 °C, partially shaded, elevation 1,349 m, 18°16'40.4"N, 100°30'19.0"E), Khun Sathan Village, Na Noi District, Nan Province, Thailand, 5-VIII-2017, by W. Srisuka (Coll. No. 66).

***Paratypes***: One female, nine males (thoraces of two males for DNA analysis) (with their associated pupal exuviae and cocoons), and 10 mature larvae (one mature larva for DNA analysis) (in 80% ethanol), same data as for holotype.

**Figure 12.**
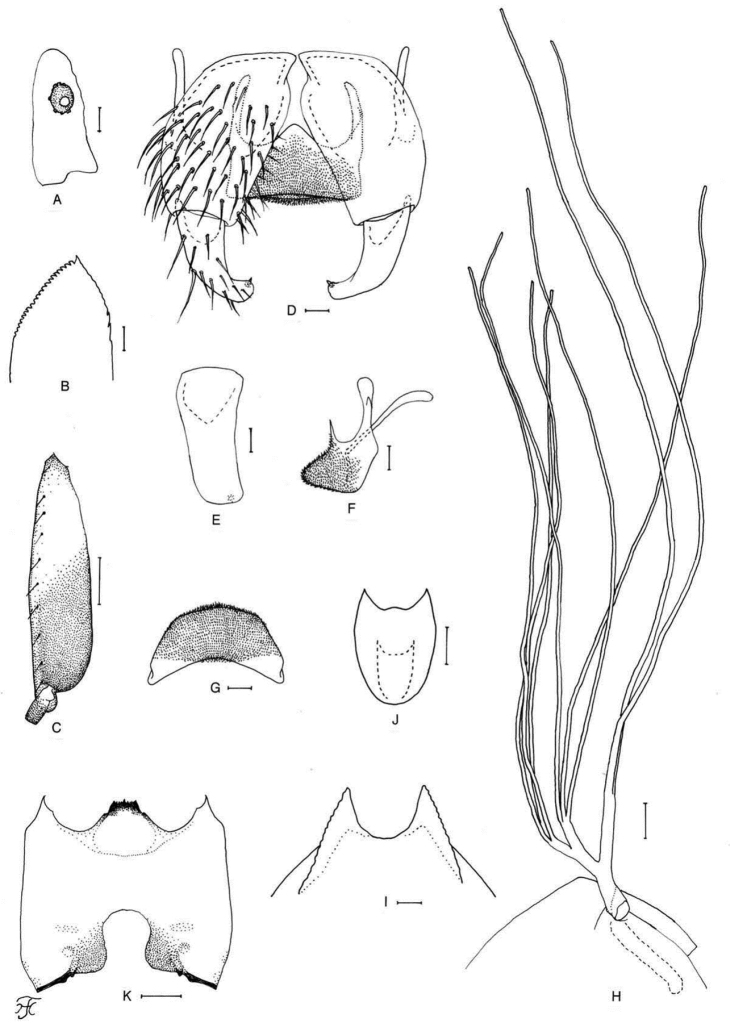
Female, male, pupa and larva of *S. nanoiense* sp. nov. **A, B** female **C–G** male **H–J** pupa **K** larva. **A** sensory vesicle (right side; anterior view) **B** mandible (right side) **C** hind basitarsus and second tarsomere (left side; lateral view) **D** coxites, styles and ventral plate (ventral view) **E** style (right side; ventrolateral view) **F** ventral plate and median sclerite (lateral view) **G** ventral plate (caudal view) **H** gill filaments (right side; lateral view) **I** terminal hooks (caudal view) **J** cocoon (dorsal view) **K** head capsule (ventral view). Scale bars: 1.0 mm (**J**); 0.1 mm (**C, H, K**); 0.02 mm (**A, D–G**); 0.01 mm(**B, I**).

**Diagnosis.** Female: small sensory vesicle 0.22–0.24 times as long as the third palpal segment (Fig. 12A) and mandible with two teeth on the outer margin (Fig. 12B). Male: medium number of upper-eye (large) facets in 12 or 13 vertical columns and 14 (rarely 13) horizontal rows. Larva: postgenal cleft as long as or little longer than the postgenal bridge (Fig. 12K) and abdominal segments 1–3, 7, and 8 greyish (Fig. 25J).

##### Description.

**Female** (*N* = 1). Body length 2.0 mm.

***Head.*** Frontal ratio 1.8:1.0:2.7; frons:head ratio 1.0:5.0. Labrum 0.66 times length of clypeus. Maxillary palpus: proportional lengths of third, fourth, and fifth palpal segments 1.0:1.1:2.4; sensory vesicle (Fig. 12A) small, ellipsoidal (0.22–0.24 times length of third palpal segment). Lacinia with ten or eleven inner and 13 outer teeth. Mandible (Fig. 12B) with 21 inner teeth and two outer teeth at some distance from tip.

***Legs.*** Foreleg: tibia yellowish white except apical three-tenths brownish black; basitarsus moderately dilated, 7.1 times as long as its greatest width. Hind leg: coxa light brown; tibia yellowish white on little more than basal half and light brown to brownish black on rest; tarsus brownish black except basal three-tenths (though base light brown) and basal half of second tarsomere yellowish white; basitarsus 6.2 times as long as wide, and 0.7 and 0.6 times as wide as greatest widths of tibia and femur, respectively; calcipala nearly as long as width at base, and 0.6 times as wide as greatest width of basitarsus.

***Wing.*** Length 2.2 mm.

***Abdomen.*** Basal scale ochreous, with fringe of whitish yellow hairs. Dorsal surface of abdomen medium to dark brown except anterior four-fifths of segment 2 ochreous.

***Terminalia.*** Sternite 8 bare medially, with 27 or 28 medium-long to long hairs together with two to four slender short hairs on each side. Ovipositor valves each moderately covered with microsetae interspersed with two short hairs. Paraproct in ventral view with four or five sensilla on anteromedial surface; paraproct in lateral view 0.6 times as long as wide, with 19–21 medium-long to long hairs on ventral and lateral surfaces. Cercus in lateral view 0.5 times as long as wide. Spermatheca 1.5 times as long as its greatest width.

**Male** (*N* = 10). Body length 2.1–2.3 mm.

***Head.*** Slightly wider than thorax. Upper eye medium brown, consisting of large facets in 12 or 13 vertical columns and 14 (rarely 13) horizontal rows. Antenna: first flagellomere elongate, 1.7 times length of second. Maxillary palpus: proportional lengths of third, fourth, and fifth palpal segments 1.0:1.3:2.6; sensory vesicle 0.14–0.17 times length of third palpal segment.

***Legs.*** Foreleg: tibia whitish yellow except little more than apical one-third dark brown, and small subbasal area on inner and lateral surfaces light brown; basitarsus somewhat dilated, 7.0–7.1 times as long as its greatest width. Midleg: tarsus dark brown except basal one-fourth to one-third of basitarsus dark yellow (border not well defined). Hind leg: coxa light brown; femur light to medium brown with base yellow and apical cap brownish black (though apical tip yellow); tibia dark brown to brownish black except basal half or little less whitish yellow; tarsus (Fig. 12C) brownish black except basal two-fifths of basitarsus and basal one-third of second tarsomere whitish yellow; basitarsus (Fig. 12C) 3.8–4.0 times as long as wide, and 0.9–1.0 and 1.1 times as wide as greatest width of tibia and femur, respectively; calcipala (Fig. 12C) slightly shorter than basal width, and 0.32 times as wide as greatest width of basitarsus.

***Wing.*** Length 1.9–2.0 mm. Subcosta bare in three males but with one to seven hairs in six males.

***Genitalia.*** Style in ventrolateral view (Fig. 12E) slightly tapered from base to middle, then nearly parallel-sided toward apex, with truncated apex, and 0.8 times as long as coxite. Ventral plate in ventral view (Fig. 12D) with basal arms slightly convergent apically; ventral plate in caudal view (Fig. 12G) rounded ventrally. Cercus small, rounded, with 12 or 13 hairs.

**Pupa** (*N* = 11). Body length 2.4–2.6 mm.

***Head.*** Integument yellow. Thorax. Integument yellow, moderately covered with round tubercles except dorsal and dorsolateral surfaces of posterior half almost bare or sparsely covered with tubercles, though small dorsal area near posterior margin sparsely to moderately covered with tubercles.

***Thorax.*** Gill (Fig. 12H) composed of eight slender thread-like filaments, arranged as (3+3)+2 or [(2+1)+3]+2) or [3+(1+2)]+2 from dorsal to ventral; common basal stalk 0.7–0.8 times length of interspiracular trunk; dorsal and middle triplets sharing short stalk, and mostly composed of three individual filaments arising at same level; stalk of ventral pair of filaments variable in length, 0.9–1.1 times length of common basal stalk, and 0.7–0.8 times length of interspiracular trunk; primary stalk of dorsal triplet lying against that of lower pair at angle of 70–80° when viewed laterally; filaments of dorsal triplet subequal in length (1.6–1.9 mm) and thickness to one another; filaments of middle triplets subequal in length (2.0–2.3 mm) and thickness to one another; two filaments of ventral pair subequal in length (2.5–3.0 mm) and thickness to each other and 1.4 times as thick as six other filaments of dorsal and middle triplets when compared basally.

***Abdomen.*** Dorsally, all segments light yellowish; segments 1 and 2 without tubercles; segment 9 with pair of wide flat terminal hooks (Fig. 12I), of which outer margin 2.0–2.1 times length of inner margin and crenulated when viewed caudally.

***Cocoon*** (Fig. 12J). Light yellow, slipper-shaped, roughly to moderately woven, widely extended ventrolaterally; anterior margin not thickly woven medially, without bulge or short projection; individual threads visible or not; 2.9–3.2 mm long by 2.0–2.8 mm wide.

**Mature larva** (*N* = 9). Body length 5.0–5.6 mm. Body creamy white with following color markings: thoracic segment 1 encircled with dark grey band (though disconnected ventromedially), dorsal surface of thoracic segments 1–3 often light grey except posterior half of segment 3 light ochreous; ventral surface of thoracic segment 2 dark grey; ventral surface of thoracic segment 3 light grey on anterior one-third and light ochreous on posterior two-thirds; abdominal segments 1–3 entirely grey, abdominal segments 7 and 8 light grey dorsally, abdominal segment 4 with purplish transverse band (though often partially faded, leaving narrow band or small spot(s) dorsomedially), abdominal segments 5 and 6 each with distinct, dark purplish, W-shaped, transverse band dorsally (though that on segment 6 often faded partially, leaving one distinct round dorsomedial spot and two dorsolateral spots), dorsal and dorsolateral surfaces of abdominal segments 7 and 8 each covered with dark purplish pigment, though dorsomedial portions usually faded (Fig. 25J).

***Head.*** Head spots faintly or moderately positive. Antenna: proportional lengths of first, second, and third articles 1.0:0.7:0.8. Labral fan with 33–38 primary rays. Postgenal cleft (Fig. 12K) small, rounded, 1.0–1.1 times length of postgenal bridge.

***Thorax*** and ***Abdomen***. Thoracic and abdominal cuticle sparsely covered with unpigmented minute setae dorsally except last abdominal segment densely covered with unbranched colorless minute setae on dorsolateral and lateral surfaces of each side of anal sclerite and on each lateral surface even down to base of ventral papilla. Rectal organ compound, each of three lobes with ten or eleven finger-like secondary lobules. Anal sclerite with anterior arms slightly longer than posterior ones. Posterior circlet with 85–88 rows of hooklets with up to 14 hooklets per row.

##### Etymology.

The species name, *nanoiense*, refers to the district, Na Noi, where this species was collected.

##### Distribution.

Thailand (Nan).

##### Discussion.

This new species is similar to *S. brinchangense* described from Peninsular Malaysia (Takaoka et al. 2014b), in having a similar number of male upper-eye facets but is distinguished by the male ventral plate with its ventral margin rounded (Fig. 12G) when viewed posteriorly (ventral margin is nearly straight or slightly concave in *S. brinchangense*).

This new species is similar in larval body color to *S. junkumae* sp. nov. but is distinguished from the latter species by the dorsal and dorsolateral surfaces of larval abdominal segments 7 and 8 light grey (Fig. 25J).

#### Simulium (Gomphostilbia) muangpanense

Taxon classificationAnimaliaDipteraSimuliidae

Takaoka, Srisuka & Fukuda sp. nov.

97A51E2F-A553-58F2-929F-12EA88AF17DC

http://zoobank.org/637B60FF-279B-4E5C-988D-BDDB11C4A469

Figs 13 , 25O

##### Material examined.

***Holotype***: Male (with its associated pupal exuviae and cocoon) (in 80% ethanol) labeled as “Holotype: *Simulium muangpanense* male, QSBG col. no. 86, Thailand, 9-VIII-2016, by W. Srisuka”, collected from a stream (width 30 cm, depth 5 cm, bed sandy, moderate running, pH 6.3, 22.6 °C, exposed to the sun, elevation 1,097 m, 18°50'03.7"N, 99°22'32.2"E), at Pa Meing Village, Muang Pan District, Lampang Province, northern Thailand, 9-VIII-2016, by W. Srisuka (Coll. No. 86).

***Paratypes***: Three females (thorax of one female for DNA analysis), four males (thorax of one male for DNA analysis) (with their associated pupal exuviae and cocoons), and five mature larvae (one mature larva for DNA analysis) (in 80% ethanol), same data as for holotype.

##### Diagnosis.

**Figure 13.**
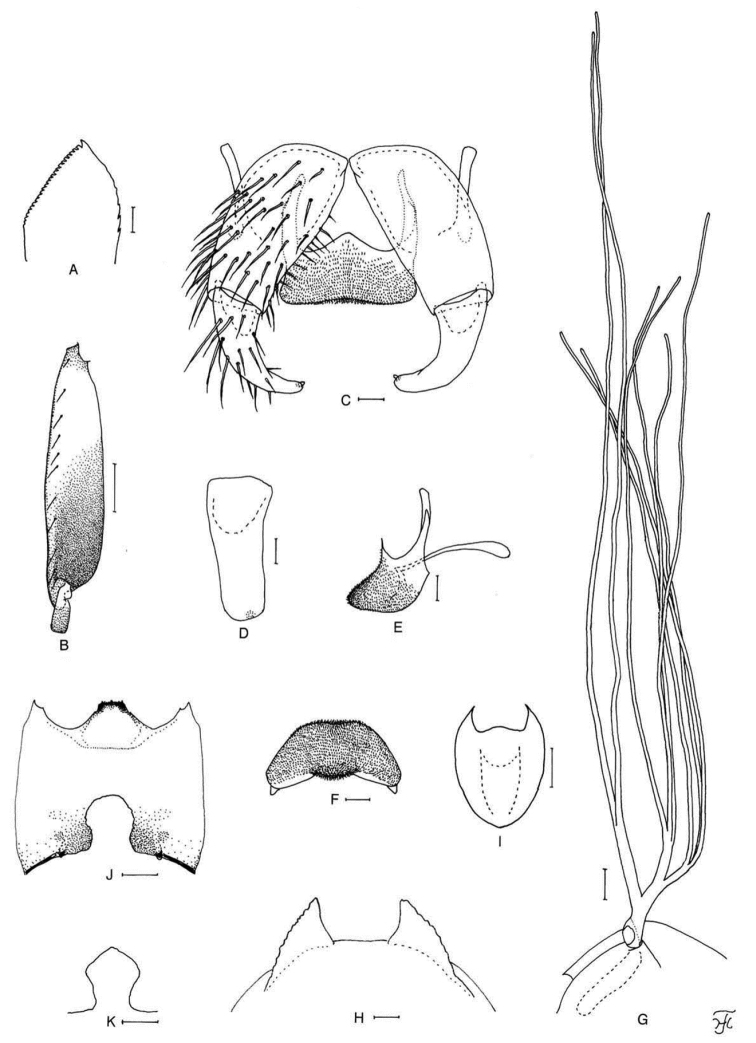
Female, male, pupa and larva of *S. muangpanense* sp. nov. **A** female **B–F** male **G–I** pupa **J, K** larva. **A** mandible (right side) **B** hind basitarsus and second tarsomere (left side; lateral view) **C** coxites, styles and ventral plate (ventral view) **D** style (right side; ventrolateral view) **E** ventral plate and median sclerite (lateral view) **F** ventral plate (caudal view) **G** gill filaments (left side; lateral view) **H** terminal hooks (caudal view) **I** cocoon (dorsal view) **J** head capsule (ventral view) **K** postgenal cleft. Scale bars: 1.0 mm (**I**); 0.1 mm (**B, G, J, K**); 0.02 mm (**C–F**); 0.01 mm (**A, H**).

Female: mandible with two or three outer teeth (Fig. 13A). Male: number of upper-eye facets in 13 vertical columns and 14 horizontal rows, hind basitarsus (Fig. 13B) 0.9 times as wide as the hind femur. Larva: abdominal segments 1, 4, 5, 7, and 8 grey at least on dorsal and dorsolateral surface (Fig. 25O).

##### Description.

**Female** (*N* = 3). Body length 2.0–2.1 mm.

***Head.*** Frontal ratio 1.8:1.0:2.1; frons:head ratio 1.0:4.0. Labrum 0.61 times length of clypeus. Maxillary palpus: proportional lengths of third, fourth, and fifth palpal segments 1.0:1.0:2.5; sensory vesicle medium sized, ellipsoidal (0.29–0.31 times length of third palpal segment), with small opening. Lacinia with nine or ten inner and 14 outer teeth. Mandible (Fig. 13A) with 20 inner teeth and two or three outer teeth at some distance from tip.

***Legs.*** Foreleg: basitarsus moderately dilated, 6.4 times as long as its greatest width. Midleg: femur light brown with basal one-fourth whitish yellow and apical cap medium brown (though extreme tip yellowish); tarsus dark brown to brownish black though little less than basal half of basitarsus yellow (its border not well defined). Hind leg: coxa light brown with apical one-third yellowish white; tibia white to yellowish white on basal two-thirds and light brown to brownish black on rest; basitarsus 6.04 times as long as wide, and 0.76 and 0.58 times as wide as greatest widths of tibia and femur, respectively; calcipala nearly as long as or lightly longer than width at base, and 0.5–0.6 times as wide as greatest width of basitarsus.

***Wing.*** Length 2.0 mm.

***Terminalia.*** Sternite 8 with 18–21 medium-long to long hairs together with four slender short hairs on each side. Ovipositor valve moderately covered with microsetae interspersed with two or three short hairs. Paraproct in ventral view with four or five or six sensilla on anteromedial surface; paraproct in lateral view 0.6 times as long as wide, with 24–28 medium-long to long hairs on ventral and lateral surfaces. Cercus in lateral view 0.46 times as long as wide.

**Male** (*N* = 5). Body length 2.1–2.5 mm.

***Head.*** Slightly wider than thorax. Upper eye dark brown, consisting of large facets in 13 vertical columns and 14 horizontal rows on each side. Antenna: first flagellomere 1.5–1.7 times length of second. Maxillary palpus: proportional lengths of third, fourth, and fifth palpal segments1.0:1.2:2.9–3.1; sensory vesicle globular or ellipsoidal, 0.15–0.18 times length of third palpal segment.

***Legs.*** Foreleg: femur light brown except apical cap medium brown (though apical tip yellowish); tibia light brown on basal one-third (though basal tip yellow and outer surface narrowly yellowish white), yellowish white on middle one-third, and brownish black on apical one-third; basitarsus moderately dilated, 6.7–7.3 times as long as its greatest width. Midleg: femur light brown with base yellowish on inner surface and apical cap medium brown (though apical tip yellowish); tibia medium brown except basal one-third (or little more on posterior surface) yellowish white; tarsus dark brown except base of basitarsus dark yellow to light brown (border not well defined). Hind leg: coxa light brown with apical one-third yellowish; femur light to medium brown with base whitish yellow and apical cap brownish black (though apical tip yellow); tibia dark brown to brownish black except basal half or little less whitish yellow; tarsus dark brown except basal two-fifths of basitarsus and little less than basal half of second tarsomere whitish yellow; basitarsus (Fig. 13B) 4.0–4.2 times as long as wide, and 0.9 and 0.9 times as wide as greatest width of tibia and femur, respectively; calcipala (Fig. 13B) slightly shorter than basal width, and 0.33 times as wide as greatest width of basitarsus.

***Wing.*** Length 1.6–1.9 mm. Other characters as in female except subcosta with 0–6 hairs.

***Genitalia.*** Coxite in ventral view (Fig. 13C) 1.9 times as long as its greatest width. Style in ventral view (Fig. 13D) with round apex, and in ventrolateral view (Fig. 13D) slightly tapered toward apex or nearly parallel-sided from middle to apex, and 0.8 times length of coxite. Ventral plate in ventral view (Fig. 13C) with basal arms nearly parallel-sided, then convergent apically; ventral plate in caudal view (Fig. 13F) rounded ventrally, though ventral margin slightly concave medially. Ventral surface of abdominal segment 10 moderately sclerotized along anterior margin. Cercus with 13–16 hairs.

**Pupa** (*N* = 8). Body length 2.3–2.6 mm.

***Thorax.*** Gill (Fig. 13G) composed of eight slender thread-like filaments, arranged as [(2+1)+(1+2)]+2 or [3+(1+2)]+2 from dorsal to ventral; common basal stalk 0.6–0.8 times length of interspiracular trunk; stalk of ventral pair of filaments variable in length, 1.0–1.5 times length of common basal stalk, and 0.7–1.0 times length of interspiracular trunk; filaments of dorsal triplet subequal in length to one another (2.0–2.2 mm) and those of middle triplet subequal in length to one another (2.4–2.5); two filaments of ventral pair subequal in length to each other (3.0–3.2 mm) and thickness to each other and 1.6 times as thick as six other filaments of dorsal and middle triplets when compared basally.

***Abdomen.*** Dorsally, all segments pale yellowish; segments 1 and 2 almost bare or sparsely covered with minute tubercles; segment 9 with pair of flat terminal hooks (Fig. 13H), of which outer margin 2.8–3.1 times length of inner margin and crenulated when viewed caudally.

***Cocoon*** (Fig. 13I). Slipper-shaped, moderately woven, moderately extended ventrolaterally; anterior margin thickly woven medially, rarely with bulge; 3.0–3.4 mm long by 2.0–2.5 mm wide.

**Mature larva** (*N* = 4). Body length 4.8–5.0 mm. Body ochreous with following color markings: thoracic segment 1 encircled with grey band (though disconnected ventromedially), thoracic segments 2 and 3 grey on ventral surface (though often faded on segment 3); abdominal segments 1 and 4 entirely grey, abdominal segments 5, 7, and 8 grey on dorsal and dorsolateral surface; abdominal segment 4 with faint narrow reddish brown transverse band on dorsal and dorsolateral surface in one larva; abdominal segment 5 with distinct reddish brown, W-shaped, transverse band dorsally, abdominal segment 6 often with three reddish brown spots (one round dorsomedial spot and two lateral spots of various size and shape), and abdominal segments 7 and 8 distinctly covered with reddish brown pigment usually on dorsolateral surface (Fig. 25O).

***Head.*** Head capsule yellow with narrow area along posterior margin darkened; head spots faintly or moderately positive. Antenna: proportional lengths of first, second, and third articles 1.0:0.7–0.8:0.8–0.9. Labral fan with 31 or 32 primary rays. Hypostoma with five hypostomal bristles per side lying nearly parallel to lateral margin. Postgenal cleft (Fig. 13J, K) medium-long, 1.2–2.1 times length of postgenal bridge, usually with apical margin round (though angulated in one larva).

***Thorax*** and ***Abdomen.*** Thoracic and abdominal cuticle almost bare except abdominal segments 5–8 moderately covered with unbranched dark minute setae (though setae rarely bifid) on dorsal and dorsolateral surface. Rectal organ compound, each of three lobes with seven to nine finger-like secondary lobules. Anal sclerite with anterior arms 1.1–1.2 times as long as posterior ones. Posterior circlet with 76–80 rows of hooklets with up to 14 or 15 hooklets per row.

##### Etymology.

The species name, *muangpanense*, refers to the district, Muang Pan, where this species was collected.

##### Distribution.

Thailand (Lampang).

##### Discussion.

The male of this new species is similar to *S. phulocense* from Vietnam (Takaoka et al. 2015) by having the same number of upper-eye (large) facets, but is distinguished in the female by the fore basitarsus 5.8 times as long as its greatest width (6.9 times in *S. phulocense*), in the male by the fore basitarsus 6.7–7.3 times as long as its greatest width (8.3 times in *S. phulocense*), and ventral plate trapezoidal ventrally when viewed posteriorly (rounded ventrally in *S. phulocense*) and in the pupa by the cocoon without an anterodorsal projection (with an anterodorsal projection in *S. phulocense*).

*Simulium tanahrataense* from Peninsular Malaysia (Takaoka et al. 2014b) and *S. confertum* Takaoka & Sofian-Azirun from Vietnam (Takaoka et al. 2015) have a similar number of male upper-eye facets (in 14 vertical columns and 15 horizontal rows) and a similar shape of the ventral plate (trapezoidal in caudal view), but are barely distinguished from this new species by the male hind basitarsi as wide as the hind femora (0.9 times as wide as the hind femur in this new species) and the length of the ventral paired filaments of the pupa 2.6–2.7 mm (3.0–3.2 mm in this new species).

The larva of this new species is almost the same in body color pattern as *S. monglaense* described from Myanmar (Takaoka et al. 2017b) but is barely distinguished from the latter species by the posterior circlet with 76–80 rows (85–93 rows in *S. monglaense*). This new species is distinguished from *S. monglaense* in the female by the mandible with distinct outer teeth and in the male by the relative length of the fore basitarsus against its greatest width (6.7–7.3 in this new species versus 8.0 in in *S. monglaense*).

#### Simulium (Gomphostilbia) maehongsonense

Taxon classificationAnimaliaDipteraSimuliidae

Takaoka, Srisuka & Saeung sp. nov.

8E6F2C6F-F587-5C75-B991-D960297CD504

http://zoobank.org/BD4BF904-907D-460F-9276-168D189BC582

Figs 14 , 25P

##### Material examined.

***Holotype***: Male (with its associated pupal exuviae and cocoon) (in 80% ethanol) labeled as “Holotype: *Simulium maehongsonense* male, QSBG col. no. 107, Thailand, 12-VII-2017, by W. Srisuka”, collected from a stream (width 120 cm, depth 2 cm, bed sandy, fast flow, pH 7.6, 21.2 °C, partially shade, elevation 1,034 m, 19°36'58.5"N, 97°59'48.2"E), at Pha Peung, Muang District, Mae Hong Son Province, Thailand, 12-VII-2017, by W. Srisuka (Coll. No. 107).

***Paratypes***: Two females (thorax of one female for DNA analysis), seven males (thorax of one male for DNA analysis) (with their associated pupal exuviae and cocoons), and six mature larvae (one mature larva for DNA analysis) (in 80% ethanol), same data as for holotype.

##### Diagnosis.

**Figure 14.**
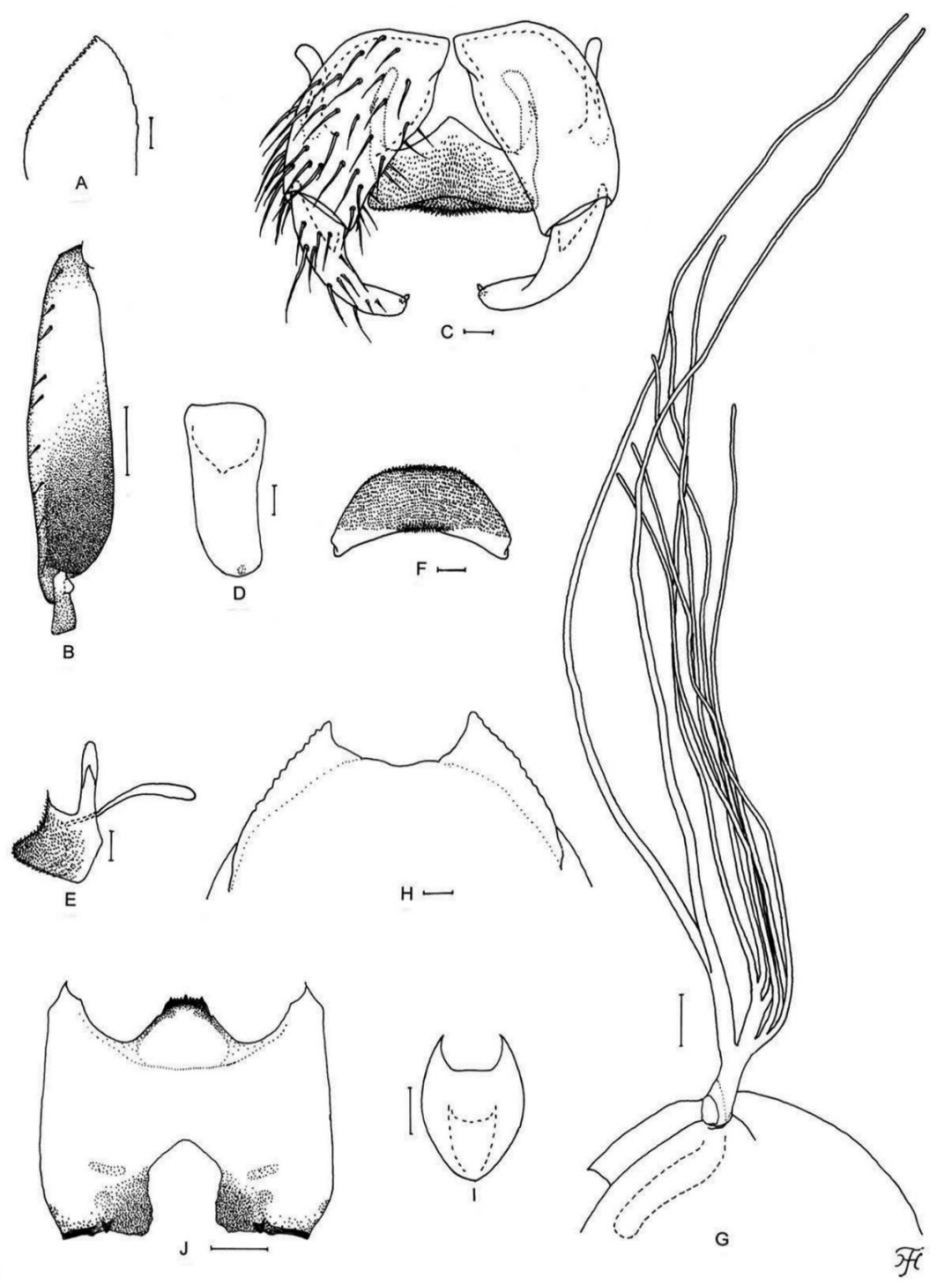
Female, male, pupa and larva of *S. maehongsonense* sp. nov. **A** female **B–F** male **G–I** pupa **J** larva. **A** mandible (right side) **B** hind basitarsus and second tarsomere (left side; lateral view) **C** coxites, styles and ventral plate (ventral view) **D** style (right side; ventrolateral view) **E** ventral plate and median sclerite (lateral view) **F** ventral plate (caudal view) **G** gill filaments (left side; lateral view) **H** terminal hooks (caudal view) **I** cocoon (dorsal view) **J** head capsule (ventral view). Scale bars: 1.0 mm (**I**); 0.1 mm (**B, G, J**); 0.02 mm (**C–F**); 0.01 mm (**A, H**).

Female: short sensory vesicle and mandible lacking distinct outer teeth (Fig. 14A). Male: number of upper-eye facets in 13 vertical columns and 14 horizontal rows. Pupa: relatively shorter gill filaments (longest 2.1–2.3 mm long) (Fig. 14G) and relatively wider terminal hooks (Fig. 14H). Larva: postgenal cleft 1.4–2.2 times as long as the postgenal bridge (Fig. 14J) and abdominal segment 1 light grey (Fig. 25P).

##### Description.

**Female** (*N* = 2). Body length 1.9 mm.

***Head.*** Frontal ratio 1.9:1.0:2.1; frons:head ratio 1.0:4.2. Labrum 0.52 times length of clypeus. Maxillary palpus: proportional length of third, fourth and fifth palpal segments 1:1.1:2.2; sensory vesicle medium-long (0.26 times length of third palpal segment. Lacinia with 9–11 inner and 11–13 outer teeth. Mandible (Fig. 14A) with 23 inner teeth and lacking distinct outer teeth (though three vestigial teeth present at some distance from tip).

***Legs.*** Foreleg: tibia yellowish white except apical three-tenths brownish black; basitarsus moderately dilated, 5.5 times as long as its greatest width. Midleg: tarsus dark brown though basal half of basitarsus yellow (its border not well defined). Hind leg: coxa light brown; tibia yellowish white on basal two-thirds and light brown to brownish black on rest; basitarsus 6.7 times as long as wide, and 0.6 and 0.5 times as wide as greatest widths of tibia and femur, respectively; calcipala 0.5 times as wide as greatest width of basitarsus; claw with large basal tooth 0.46 times length of claw.

***Wing.*** Length 1.9 mm.

***Abdomen.*** Dorsal surface of abdomen medium to dark brown except most of segment 2 ochreous (though narrow portion along posterior margin somewhat darkened).

***Terminalia.*** Sternite 8 bare medially, with 20–22 medium-long to long hairs together with two or three slender short hairs on each side. Ovipositor valves each moderately covered with microsetae interspersed with two or three short hairs. Paraproct in lateral view 0.6 times as long as wide, with 18 medium-long to long hairs on ventral and lateral surfaces. Spermatheca ellipsoid, 1.3 times as long as its greatest width.

**Male** (*N* = 8). Body length 2.0–2.4 mm.

***Head.*** Slightly wider than thorax. Upper eye dark brown, consisting of large facets in 13 vertical columns and 14 horizontal rows on each side. Maxillary palpus: proportional lengths of third, fourth, and fifth palpal segments 1.0:1.2:2.8; sensory vesicle 0.17 times length of third palpal segment.

***Legs.*** Foreleg: tibia yellowish white except l apical one-third dark brown, and inner and lateral surfaces of subbasal portion light brown; basitarsus moderately dilated, 6.5–7.3 times as long as its greatest width. Hind leg: coxa light brown; tibia dark brown to brownish black except little less than basal half yellowish white; tarsus dark brown except little less than basal half of basitarsus and basal one-third of second tarsomere yellowish; basitarsus (Fig. 14B) 3.8–3.9 times as long as wide, and 1.0 and 1.0 times as wide as greatest width of tibia and femur, respectively; calcipala (Fig. 14B) 0.32 times as wide as greatest width of basitarsus.

***Wing.*** Length 1.9–2.0 mm. Subcosta with four to nine hairs in four males but bare in four males.

***Genitalia.*** Coxite in ventral view (Fig. 14C) 1.8 times as long as its greatest width. Style in ventral view (Fig. 14C) with round apex; style in ventrolateral view (Fig. 14D) slightly tapered toward apex, with round apex. Ventral plate in ventral view (Fig. 14C) with basal arms of moderate length, nearly parallel- sided, then convergent apically; ventral plate in caudal view (Fig. 14F) with ventral margin nearly straight or slightly concave. Cercus with 14 hairs.

**Pupa** (*N* = 10). Body length 2.5–2.7 mm.

***Head.*** Integument yellow. Thorax. Integument yellow.

***Thorax.*** Gill (Fig. 14G) composed of eight slender thread-like filaments, arranged as (3+3) +2 or [3+(1+2)]+2 from dorsal to ventral; common basal stalk 0.5–0.7 times length of interspiracular trunk; dorsal triplet with extremely short stalk; stalk of ventral pair of filaments 0.9–1.5 times length of common basal stalk, and 0.5–0.8 times length of interspiracular trunk; primary stalk of dorsal triplet lying against that of lower pair at angle of 70° when viewed laterally; filaments of dorsal triplet subequal in length (1.1–1.3 mm) and thickness to one another; filaments of middle triplet subequal in length (1.5–1.7 mm) and thickness to one another; two filaments of ventral pair subequal in length (2.1–2.3 mm) and thickness to each other and 1.6 times as thick as six other filaments of dorsal and middle triplets when compared basally.

***Abdomen.*** Dorsally, all segments light yellowish; segments 1 and 2 without minute tubercles; segment 9 with pair of wide flat terminal hooks (Fig. 14H), of which outer margin 3.3–4.0 times length of inner margin and crenulated when viewed caudally.

***Cocoon*** (Fig. 14I). Pale yellow, slipper-shaped, roughly to moderately woven, moderately extended ventrolaterally; anterior margin thickly woven medially, without bulge or short projection; 3.0–3.5 mm long by 2.0–2.9 mm wide.

**Mature larva** (*N* = 5). Body length 4.5–5.5 mm. Body light ochreous with following color markings: thoracic segment 1 encircled with distinct greyish band (though disconnected ventromedially), thoracic segment 2 greyish and thoracic segment 3 ochreous on ventral surface; abdominal segment 1 light grey, abdominal segments 5 and 6 each with distinct reddish brown spot of various size and shape dorsomedially, and dorsal and dorsolateral surface of abdominal segments 4–8 faintly to moderately covered with pinkish to reddish brown pigments (Fig. 25P).

***Head.*** Head spots moderately positive. Antenna: proportional lengths of first, second, and third articles 1.0:0.8:0.7–0.9. Labral fan with 38–40 primary rays. Hypostoma with four to six hypostomal bristles per side lying nearly parallel to lateral margin. Postgenal cleft (Fig. 14J) medium sized, 1.4–2.2 times length of postgenal bridge.

***Abdomen.*** Posterior circlet with 88–96 rows of hooklets with up to 14 or 15 hooklets per row.

##### Etymology.

The species name, *maehongsonense*, refers to the province, Mae Hong Son, where this species was collected.

##### Distribution.

Thailand (Mae Hong Son).

##### Discussion.

The male of this new species is similar to *S. phulocense* from Vietnam (Takaoka et al. 2015) by having the same number of upper-eye large facets, but is distinguished by the fore basitarsus 6.5–7.3 times as long as its greatest width (8.3 times in *S. phulocense*), and ventral plate trapezoidal ventrally (Fig. 14F) when viewed posteriorly (rounded ventrally in *S. phulocense*) and in the pupa by the cocoon without an anterodorsal projection (Fig. 14I) (with an anterodorsal projection in in *S. phulocense*).

#### Simulium (Gomphostilbia) chaowaense

Taxon classificationAnimaliaDipteraSimuliidae

Takaoka, Srisuka & Saeung sp. nov.

B576CECF-E482-5BD7-A692-4CFF660F762B

http://zoobank.org/20CF4BE9-8A76-48F6-A1A8-C37CA48336D7

Figs 15 , 25G

##### Material examined.

***Holotype***: Male (with its associated pupal exuviae and cocoon) (in 80% ethanol) labeled as “Holotype: *Simulium chaowaense* male, QSBG col. no. 164, Thailand, 16-III-2017, by W. Srisuka”, collected from a small stream (width 40 cm, depth 3.5 cm, bed sandy, moderate flow, pH 7.3, 21.9 °C, partially, elevation 582 m, 18°45'30.2"N, 100°20'11.4"E), Chao Wa Waterfall, Song District, Phrae Province, Thailand, 16-III-2017, by W. Srisuka (Coll. No. 164).

***Paratypes***: Three males (thorax of one male for DNA analysis) (with their associated pupal exuviae and cocoons), and six mature larvae (one mature larva for DNA analysis) (in 80% ethanol), same data as in the holotype

##### Diagnosis.

**Figure 15.**
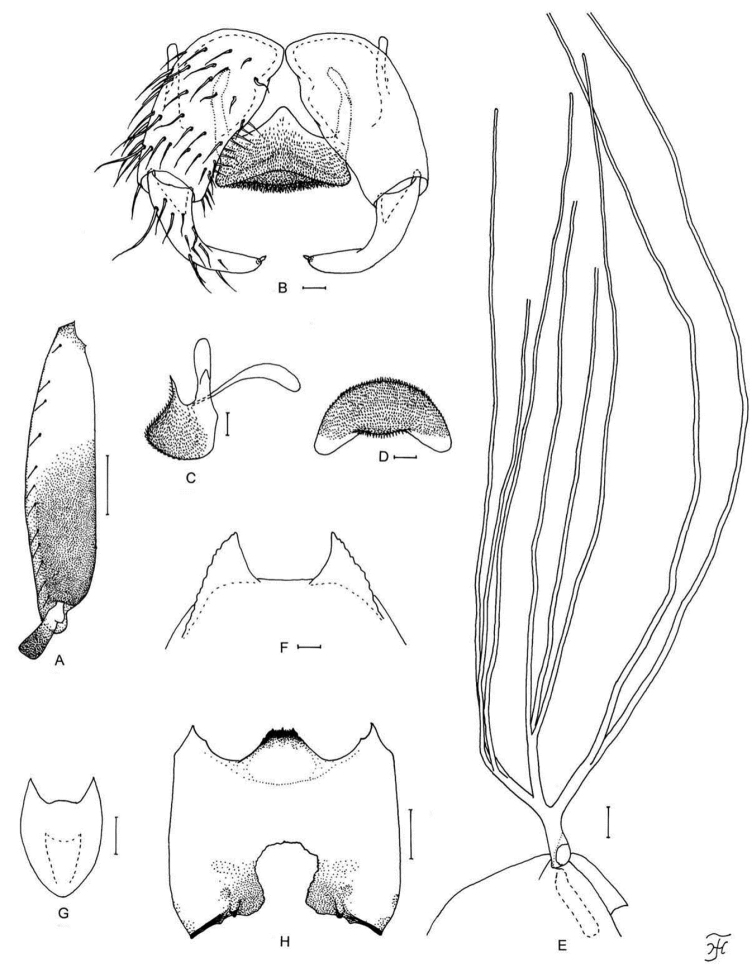
Male, pupa and larva of *S. chaowaense* sp. nov. **A–D** male **E–G** pupa **H** larva. **A** hind basitarsus and second tarsomere (left side; lateral view) **B** coxites, styles and ventral plate (ventral view) **C** ventral plate and median sclerite (lateral view) **D** ventral plate (caudal view) **E** gill filaments (right side; lateral view) **F** terminal hooks (caudal view) **G** cocoon (dorsal view) **H** head capsule (ventral view). Scale bars: 1.0 mm (**G**); 0.1 mm (**A, E, H**); 0.02 mm (**B–D**); 0.01 mm (**F**).

Male: number of upper-eye facets in 14 or 15 vertical columns and 14 or 15 horizontal rows, and moderately widened hind basitarsus (Fig. 15A) 0.9 times as wide as hind femur. Larva: medium-long postgenal cleft (Fig. 15H) 1.2–1.3 times as long as the postgenal bridge and all abdominal segments greyish (Fig. 25G).

##### Description.

**Male** (*N* = 4). Body length 2.0–2.3 mm.

***Head.*** Distinctly wider than thorax. Upper eye dark brown, consisting of large facets in 14 or 15 vertical columns and 14 or 15 horizontal rows on each side. Antenna light to medium brown except scape, pedicel, and base of first flagellomere yellow; first flagellomere elongate, 1.8 times length of second. Maxillary palpus: proportional lengths of third, fourth, and fifth palpal segments 1.0:1.0–1.2:2.5–2.8; sensory vesicle globular or ellipsoidal, 0.16–0.17 times length of third palpal segment.

***Thorax.*** Scutum with faint longitudinal vittae (one median and two submedian).

***Legs.*** Foreleg: tibia whitish yellow except basal one-fourth light brown and apical one-third dark brown; basitarsus moderately dilated, 7.3–7.4 times as long as its greatest width. Hind leg: coxa light brown; femur light to medium brown with base yellow and apical cap dark brown (though apical tip yellow); tibia dark brown except little less than basal half yellow; tarsus (Fig. 15A) brownish black except little less than basal half of basitarsus and little less than basal half of second tarsomere whitish yellow; basitarsus (Fig. 15A) 3.8–4.0 times as long as wide, and 0.8–0.9 and 0.9 times as wide as greatest width of tibia and femur, respectively; calcipala (Fig. 15A) slightly shorter than basal width, and 0.36 times as wide as greatest width of basitarsus.

***Wing.*** Length 1.6–1.7 mm. Subcosta with 2–8 hairs.

***Genitalia.*** Coxite in ventral view (Fig. 15B) nearly rectangular, 1.8 times as long as its greatest width. Style in ventrolateral view slightly tapered toward apex, with truncated apex, and 0.8 times as long as coxite. Ventral plate in ventral view (Fig. 15B) with body transverse, 0.7 times as long as wide, posteroventral margin somewhat concave medially (though slightly convex medially on posterodorsal margin), and lateral margin emarginated medially; basal arms of moderate length, slightly divergent, then convergent apically; ventral plate in caudal view (Fig. 15D) rounded ventrally. Cercus with 15 or 16 hairs.

**Pupa** (*N* = 4). Body length 2.3–2.5 mm.

***Head.*** Integument yellow.

***Thorax.*** Integument yellow, moderately covered with round tubercles except posterior half sparsely covered with small tubercles on dorsolateral and lateral surfaces. Gill (Fig. 15E) composed of eight slender thread-like filaments, arranged asr [(2+1)+3]+2 from dorsal to ventral; common basal stalk 0.7–0.8 times length of interspiracular trunk; dorsal and middle triplets sharing short stalk, and dorsal and middle triplets mostly composed of one individual and two paired filaments with extremely short secondary stalk; stalk of ventral pair of filaments 1.0–1.2 times length of common basal stalk, and 0.7–0.9 times length of interspiracular trunk; primary stalk of dorsal triplet lying against that of lower pair at angle of 70–80° when viewed laterally; filaments of dorsal and middle triplets subequal in length (2.5–2.6 mm) and thickness to one another; two filaments of ventral pair subequal in length (3.0 mm) and thickness to each other (though inner filaments slightly thicker than inner one) and 1.5 times as thick as six other filaments of dorsal and middle triplets when compared basally.

***Abdomen.*** Dorsally, all segments light yellowish; segments 1 and 2 without minute tubercles; segment 9 with pair of wide flat terminal hooks (Fig. 15F), of which outer margin is 1.7–2.4 times length of inner margin and crenulated when viewed caudally.

***Cocoon*** (Fig. 15G). Slipper-shaped, moderately woven, moderately extended ventrolaterally; anterior margin without bulge or projection; individual threads visible; 3.2–3.6 mm long by 2.0–2.4 mm wide.

**Mature larva** (*N* = 5). Body length 4.1–4.5 mm. Body with following color markings: thoracic segment 1 encircled with greyish band (though disconnected ventromedially), ventral surface of thoracic segment 2 grey and that of thoracic segment 3 ochreous; abdominal segments 1–4 encircled with grey transverse band, and abdominal segments 5–8 greyish dorsally and dorsolaterally; abdominal segments 5 and 6 each overlaid with light reddish brown transverse band, appearing W-shaped, on dorsal and dorsolateral surfaces (though often faded out to varying extent, leaving small round medial spot and dorsolateral spots, or only small medial spot), and also overlaid with pair of round spots on ventral surface; abdominal segments 7 and 8 each overlaid with light reddish brown pigments to varying extent on dorsal and dorsolateral surfaces (completely faded out in one larva) (Fig. 25G); abdominal segment 7 with light reddish brown transverse band on ventral surface (often faded out, leaving pair of round spots).

***Head.*** Head capsule yellow to dark yellow except eye-spot region whitish, sparsely covered with minute setae (though moderately on dorsal surface); head spots faintly to moderately positive. Antenna: proportional lengths of first, second, and third articles 1.00:0.65–0.75:0.81–0.95. Labral fan with 34–38 primary rays. Hypostoma with row of nine apical teeth, of which median tooth little longer than each corner tooth; lateral margin smooth; four hypostomal bristles per side lying nearly parallel to lateral margin. Postgenal cleft (Fig. 15H) rounded, 1.2–1.3 times length of postgenal bridge.

***Thorax*** and ***Abdomen.*** Thoracic and abdominal cuticle very sparsely covered with unpigmented minute setae (though few posterior abdominal segments sparsely to moderately covered with dark minute unbranched or bifid setae (rarely trifid setae) on dorsal and dorsolateral surfaces; last abdominal segment densely covered with unbranched colorless minute setae on dorsolateral and lateral surfaces of each side of anal sclerite and on each lateral surface even down to base of ventral papilla. Rectal scales minute, unpigmented. Rectal organ compound, each of three lobes with seven to nine finger-like secondary lobules. Anal sclerite of usual X-form, with anterior arms 1.1 times as long as posterior ones. Posterior circlet with 73–78 rows of hooklets with up to 13 or 14 hooklets per row.

**Female.** Unknown.

##### Etymology.

The species name, *chaowaense*, refers to the name of the waterfall, Chao Wa, where this species was collected.

##### Distribution.

Thailand (Phrae).

##### Discussion.

This new species is similar to *S. tanahrataense* described from males and their associated pupal exuviae collected from Peninsular Malaysia (Takaoka et al. 2014b) in many characters including the number of male upper-eye facets. However, it is barely distinguished in the male from the latter species by the first flagellomere of the antenna 1.8 times as long as the second (2.1 times in *S. tanahrataense*), male hind basitarsus 0.9 times as wide as the hind femur (1.0 time in *S. tanahrataense*), and ventral plate with the ventral margin rounded (Fig. 15D) when viewed posteriorly (straight or slightly concave in *S. tanahrataense*). The pupa of this new species is almost indistinguishable from that of *S. tanahrataense*, although there is a slight difference in the length of filaments of the dorsal and middle triplets including their stalks and the common basal stalk (2.5–2.6 mm in this new species versus 1.6–2.2 mm in *S. tanahrataense*).

This new species is similar to *S. confertum* from Vietnam (Takaoka et al. 2017a) in having the similar number of male upper-eye large facets but is distinguished from the latter species by the ventral plate with the ventral margin rounded when viewed posteriorly (nearly straight in *S. confertum*).

#### Simulium (Gomphostilbia) pitasawatae

Taxon classificationAnimaliaDipteraSimuliidae

Takaoka, Srisuka & Saeung sp. nov.

7897D0C5-C109-5477-919A-8B5F60CE800A

http://zoobank.org/D35AD402-A33C-4560-B98A-5718CDA4B683

Figs 16 , 25B

##### Material examined.

***Holotype***: Male (with its associated pupal exuviae and cocoon) (in 80% ethanol) labeled as “Holotype: *Simulium pitasawatae* male, QSBG col. no. 49, Thailand, 2-II-2019, by W. Srisuka”, collected from a stream (width 120 cm, depth 10 cm, bed sandy, moderate flow, pH 6.6, 17.1 °C, partially shaded, elevation 1,154 m, 19°03'36.8"N, 99°19'15.7"E), Huai Mor Nuea Village, Doi Saket, Chiang Mai, Thailand, 2-II-2019, by W. Srisuka and A. Saeung (Coll. No. 49).

***Paratypes***: Three females, eight males (thorax of one male for DNA analysis) (with their associated pupal exuviae and cocoons), and 10 mature larvae (one mature larva for DNA analysis) (in 80% ethanol), same data as for holotype.

##### Diagnosis.

**Figure 16.**
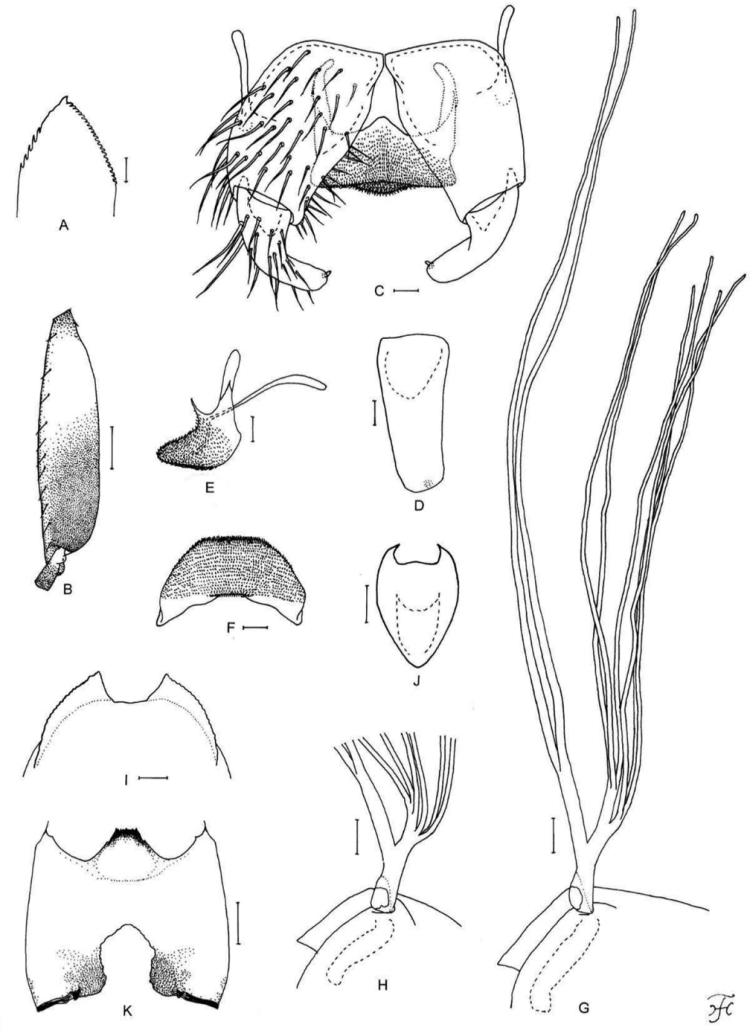
Female, male, pupa and larva of *S. pitasawatae* sp. nov. **A** female **B–F** male **G–J** pupa **K** larva. **A** mandible (left side) **B** hind basitarsus and second tarsomere (left side; lateral view) **C** coxites, styles and ventral plate (ventral view) **D** style (right side; ventrolateral view) **E** ventral plate and median sclerite (lateral view) **F** ventral plate (caudal view) **G, H** gill filaments (left side; lateral view) **I** terminal hooks (caudal view) **J** cocoon (dorsal view) **K** head capsule (ventral view). Scale bars: 1.0 mm (**J**); 0.1 mm (**B, G, H, K**); 0.02 mm (**C–F**); 0.01 mm (**A, I**).

Female: small sensory vesicle and mandible with three to five distinct teeth on outer margin (Fig. 16A). Male: number of upper-eye facets in 14 vertical columns and 15 horizontal rows. Pupa: dorsal triplet of the gill filaments without their stalk or with an extremely short stalk (Fig. 16G, H). Larva: postgenal cleft as long as or little longer than the postgenal bridge (Fig. 16K) and abdominal segments 1–4 light ochreous (Fig. 25B).

##### Description.

**Female** (*N* = 3). Body length 2.0 mm.

***Head.*** Frontal ratio 1.7–1.8:1.0:2.3–2.5; frons:head ratio 1.0:4.2–4.9. Labrum 0.61–0.64 times length of clypeus. Maxillary palpus: proportional lengths of third, fourth, and fifth palpal segments 1.0:1.1–1.2:2.0–2.3; sensory vesicle medium sized, ellipsoidal (0.32–0.35 times length of third palpal segment). Lacinia with nine or ten inner and 12–14 outer teeth. Mandible (Fig. 16A) with 20–23 inner teeth and three to five outer teeth at some distance from tip.

***Legs.*** Foreleg: basitarsus moderately dilated, 6.3–6.7 times as long as its greatest width. Hind leg: coxa medium brown; tibia yellowish white on basal three-fifths and light brown to brownish black on rest; basitarsus 5.7–6.0 times as long as wide, and 0.7–0.8 and 0.6 times as wide as greatest widths of tibia and femur, respectively; claw with large basal tooth 0.43 times length of claw.

***Wing.*** Length 2.0–2.1 mm.

***Abdomen.*** Dorsal surface of abdomen medium to dark brown except anterior one-fifths of segment 2 ochreous.

***Terminalia.*** Sternite 8 bare medially, with 19–23 medium-long to long hairs together with two to four slender short hairs on each side. Paraproct in ventral view with four sensilla on anteromedial surface; paraproct in lateral view 0.6–0.7 times as long as wide, with 18–20 medium-long to long hairs on ventral and lateral surfaces. Cercus in lateral view 0.5 times as long as wide.

**Male** (*N* = 9). Body length 2.1–2.3 mm.

***Head.*** Somewhat wide than thorax. Upper eye dark brown, consisting of large facets in 14 (rarely 13) vertical columns and 15 horizontal rows on each side. Antenna: first flagellomere elongate, 1.8 times length of second. Maxillary palpus: proportional lengths of third, fourth, and fifth palpal segments 1.0:1.1:2.4; sensory vesicle small, ellipsoidal (0.22–0.25 times length of third palpal segment).

***Legs.*** Foreleg: tibia light grey to light brown except basal tip and outer surface of basal two-thirds whitish, and apical one-third dark brown; basitarsus 7.9–8.3 times as long as its greatest width. Hind leg: coxa medium brown; tarsus (Fig. 16B) 3.7–4.0 times as long as wide, and 0.9–1.0 and 1.0–1.1 times as wide as greatest width of tibia and femur, respectively; calcipala (Fig. 16B) slightly shorter than basal width, and 0.3 times as wide as greatest width of basitarsus.

***Wing.*** Length 2.0–2.1 mm. Subcosta bare in three males but with one to five hairs in six males.

***Genitalia.*** Coxite in ventral view (Fig. 16C) nearly rectangular, 1.6 times as long as its greatest width. Style in ventrolateral view (Fig. 16D) 0.8 times length of coxite. Ventral plate in ventral view with basal arms slightly divergent, then convergent apically; ventral plate in caudal view (Fig. 16F) with ventral margin nearly straight or slightly concave medially. Paramere with basal arm bare or rarely with few minute setae on outer surface. Cercus with 15–18 hairs.

**Pupa** (*N* = 12). Body length 2.4–2.7 mm.

***Head.*** Integument yellow, Thorax. Integument yellow, moderately covered with round tubercles except dorsal and dorsolateral surfaces of posterior half sparsely covered with tubercles.

***Thorax.*** Gill (Fig. 16G, H) composed of eight slender thread-like filaments, arranged as [3+(1+2)]+2 or (3+3)+2 or (2+1+3)+2 from dorsal to ventral; common basal stalk 0.6–0.8 times length of interspiracular trunk; dorsal and middle triplets sharing short stalk, and dorsal triplet mostly composed of three individual filaments arising at same level from extremely short stalk or directly from stalk of middle triplet, middle triplet mostly composed of three individual filaments arising at same level or one individual and two paired filaments with extremely short secondary stalk; stalk of ventral pair of filaments 1.1–1.3 times length of common basal stalk, and 0.7–0.9 times length of interspiracular trunk; primary stalk of dorsal triplet lying against that of lower pair at angle of 50–60° when viewed laterally; filaments of dorsal and middle triplets subequal in length (1.7–2.0 mm) and thickness to one another; two filaments of ventral pair subequal in length (2.5–3.0 mm) and thickness to each other and 1.5 times as thick as six other filaments of dorsal and middle triplets when compared basally.

***Abdomen.*** Dorsally, all segments light yellowish; segments 1 and 2 without tubercles; segment 5 with one spine (same size as those on segments 6–9) on one side in two pupae; segment 9 with pair of wide flat terminal hooks (Fig. 16I), of which outer margin 3.6–3.9 times length of inner margin and crenulated when viewed caudally.

***Cocoon*** (Fig. 16J). Whitish yellow, slipper-shaped, roughly to moderately woven, widely extended ventrolaterally; anterior margin moderately woven medially, without bulge or short projection; individual threads invisible; 3.0–3.5 mm long by 1.7–2.4 mm wide.

**Mature larva** (*N* = 9). Body length 4.3–5.4 mm. Body light ochreous with following color markings: thoracic segment 1 encircled with light to dark brown (rarely reddish brown) band (though disconnected ventrally), thoracic segments 2 and 3 ochreous on ventral surface; abdominal segment 4 with reddish brown transverse band (though often entirely faded out), abdominal segments 5 and 6 each with distinct reddish brown, W-shaped, transverse band dorsally along posterior margin, though often partially faded out leaving one round dorsomedial spot and two lateral spots of various size and shape), dorsal and dorsolateral surface of abdominal segments 5–8 faintly to moderately covered with pinkish or reddish brown pigments (Fig. 25B).

***Head.*** Head capsule yellow except eye-spot region whitish; head spots moderately positive. Antenna: proportional lengths of first, second, and third articles 1.0:0.7–0.8:0.8–1.0. Labral fan with 23 or 24 primary rays. Postgenal cleft (Fig. 16K) rounded or pentagonal, 1.1–1.4 times length of postgenal bridge.

***Thorax*** and ***Abdomen.*** Thoracic and abdominal cuticle sparsely covered with unpigmented minute setae, though few posterior abdominal segments sparsely covered also with dark minute unbranched setae dorsally. Rectal organ compound, each of three lobes with 7–11 finger-like secondary lobules. Anal sclerite of usual X-form, with anterior arms 1.1 times as long as posterior ones. Posterior circlet with 81–85 rows of hooklets with up to 14 or 15 hooklets per row.

##### Etymology.

The species name, *pitasawatae*, is in honor of Associate Prof. Dr. Benjawan Pitasawat, Head of Department of Parasitology, Faculty of Medicine, Chiang Mai University, Thailand, who kindly supported A. Saeung in collections of black flies.

##### Distribution.

Thailand (Chiang Mai).

##### Discussion.

This new species is similar to *S. tamdaoense* Takaoka, Sofian-Azirun & Ya’cob described from Vietnam (Takaoka et al. 2014a) and *S. tanahrataense* described from Peninsular Malaysia (Takaoka et al. 2014b) in many characters including the number of male upper-eye facets and shape of the ventral plate in caudal view. However, it is distinguished from the latter two species by the dorsal triplet of the pupal gill filaments without their stalk or with an extremely short stalk (with their stalk nearly as long as the common stalk of the dorsal and middle triplet in the latter two species), from *S. tamdaoense* by the male fore basitarsus 7.9–8.3 times as long as its greatest width (6.8 times in *S. tamdaoense*) and the outer margin of the pupal terminal hook 3.7–3.9 times as long as the inner margin (1.8–1.9 times in *S. tamdaoense*), and from *S. tanahrataense* by the male first flagellomere of the antenna 1.8 times as long as the second (2.1 times in *S. tanahrataense*), and male sensory vesicle 0.22–0.25 times length of third palpal segment (0.16–0.18 time in *S. tanahrataense*).

The larva of this new species is similar in the body color pattern to S. (G.) sutheppuiense sp. nov. but is barely distinguished from the latter species by the labral fan with 23 or 24 primary rays (29 or 30 primary rays in S. (G.) sutheppuiense sp. nov.).

#### Simulium (Gomphostilbia) banluangense

Taxon classificationAnimaliaDipteraSimuliidae

Takaoka, Srisuka & Fukuda sp. nov.

066E11B7-6170-5819-895F-C0F99B65CC36

http://zoobank.org/A5D4E739-20B2-43F0-95F8-D43A76A51A62

Fig. 17

##### Material examined.

***Holotype***: Male (with its associated pupal exuviae and cocoon) (in 80% ethanol) labeled as “Holotype: *Simulium banluangense* male, Thailand, 12-VI-2001, by W. Choochote & H. Takaoka”, reared from a pupa collected from a stream (width 1.0 m, 25 °C, exposed to the sun, elevation 804 m, 18°32'33.3"N, 98°35'32.5"E), at Ban Luang, Chiang Mai Province, Thailand, 12-VI-2001, by W. Choochote and H. Takaoka.

##### Diagnosis.

**Figure 17.**
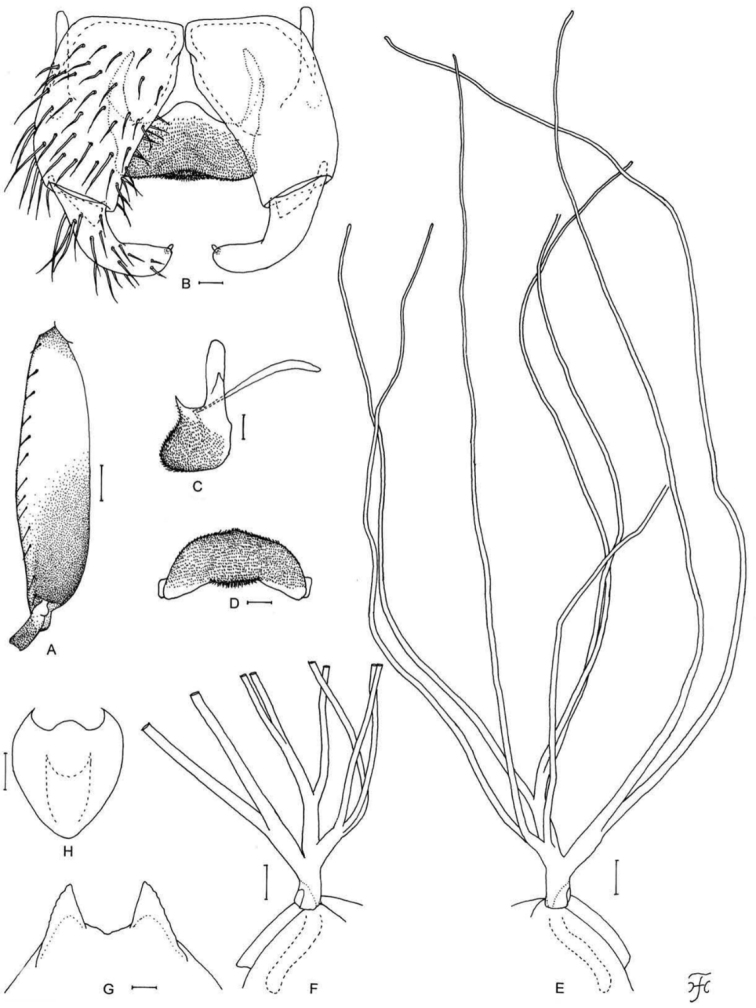
Male and pupa of *S. banluangense* sp. nov. **A–D** male **E–H** pupa. **A** hind basitarsus and second tarsomere (left side; lateral view) **B** coxites, styles and ventral plate (ventral view) **C** ventral plate and median sclerite (lateral view) **D** ventral plate (caudal view) **E, F** gill filaments (lateral view **E** right side **F** left side) **G** terminal hooks (caudal view) **H** cocoon (dorsal view). Scale bars: 1.0 mm (**H**); 0.1 mm (**A, E, F**); 0.02 mm (**B–D**); 0.01 mm (**G**).

Male: upper-eye (large) facets in 14 vertical columns and 14 or 15 horizontal rows on each side, and hind basitarsus (Fig. 17A) spindle-shaped. Pupa: gill with a short common basal stalk and a short stalk of the ventral pair of filaments, which is thicker than the interspiracular trunk (Fig. 17E, F), and terminal hooks triangular (Fig. 17G).

##### Description.

**Male** (*N* = 1). Body length 2.4 mm.

***Head.*** Slightly wider than thorax. Upper eye medium brown, consisting of 14 vertical columns and 14 or 15 horizontal rows of large facets on each side. Antenna: first flagellomere elongate, 1.9 times length of second. Maxillary palpus: proportional lengths of third, fourth, and fifth palpal segments 1.0:1.2:3.0; sensory vesicle 0.21–0.24 times length of third palpal segment.

***Legs.*** Foreleg: tibia light brown except median large area of outer surface of basal three-fourths whitish and apical one-fourth dark brown; tarsus brownish black; basitarsus moderately dilated, 6.4 times as long as its greatest width. Midleg: tarsus dark brown except basal one-fourth to two-fifths of basitarsus dark yellow to light brown (border not well defined). Hind leg: tibia dark brown to brownish black except little less than basal half whitish yellow; tarsus (Fig. 17A) brownish black except basal half of basitarsus and basal one-third of second tarsomere whitish yellow; basitarsus (Fig. 17A) 3.8 times as long as wide, and 0.9 and 0.9 times as wide as greatest width of tibia and femur, respectively; calcipala (Fig. 17A) slightly shorter than basal width, and 0.34 times as wide as greatest width of basitarsus.

***Wing.*** Length 2.1 mm. Subcosta with seven hairs.

***Genitalia.*** Coxite in ventral view (Fig. 17B) nearly rectangular, 1.6 times as long as its greatest width. Style in ventrolateral view slightly tapered toward apex, with truncated apex, and 0.8 times as long as coxite. Ventral plate in ventral view (Fig. 17B) with body transverse, 0.5 times as long as wide; ventral plate in caudal view (Fig. 17D) rounded ventrally, with ventral margin slightly raised medially. Cercus small, rounded, with 15–17 hairs.

**Pupa** (*N* = 1). Body length 3.0 mm.

***Thorax.*** Gill (Fig. 17E, F) composed of eight slender thread-like filaments, arranged as [(2+1)+(1+2)]+2 or [3+(1+2)]+2 from dorsal to ventral; common basal stalk short, 0.5–0.6 times length of interspiracular trunk; dorsal and middle triplets sharing short stalk, stalk of ventral pair of filaments short, 0.8 times length of common basal stalk, and 0.4–0.5 times length of interspiracular trunk, and thicker than interspiracular trunk; primary stalk of dorsal triplet lying against that of lower pair at angle of 90° when viewed laterally; stalk of middle triplet directed inward; filaments of dorsal and middle triplets subequal in length (2.5–2.9 mm) and thickness to one another; two filaments of ventral pair subequal in length (3.2–3.5 mm) and thickness to each other and 1.6 times as thick as six other filaments of dorsal and middle triplets when compared basally.

***Abdomen.*** Dorsally, segments 1 and 2 without minute tubercles; terminal hooks (Fig. 17G) triangular, with outer margin slightly undulate, and little less than twice length of inner margin.

***Cocoon*** (Fig. 17H). Slipper-shaped, roughly to moderately woven, widely extended ventrolaterally; anterior margin thickly woven medially, with bulge; individual threads visible; 3.5 mm long by 3.0 mm wide.

**Female** and **Larva.** Unknown.

##### Etymology.

The species name, *banluangense*, refers to the name of the locality, Ban Luang, where this species was collected.

##### Distribution.

Thailand (Chiang Mai).

##### Discussion.

*Simulium doisaketense* described based on pupal and larval specimens from Thailand (Jitklang et al. 2008), has the pupal gill with a short common basal stalk, like this new species, but the stalk of the ventral pair of filaments is medium-long, 1.3 times as long as the common basal stalk, and thinner than the interspiracular trunk, based on observations of the type specimen.

#### Simulium (Gomphostilbia) junkumae

Taxon classificationAnimaliaDipteraSimuliidae

Takaoka, Srisuka & Saeung sp. nov.

EC6F56BA-4FF3-5DCD-AB24-F5ADE0555977

http://zoobank.org/327F3EB9-C555-4B08-A691-789310BB612F

Figs 18 , 25K

##### Material examined.

***Holotype***: Male (with its associated pupal exuviae and cocoon) (in 80% ethanol) labeled as “Holotype: *Simulium junkumae* male, QSBG col. no. 92, Thailand, 7-IV-2017, by W. Srisuka”, collected from a small stream (width 40 cm, depth 3 cm, bed sandy, moderate flow, pH 6.2, 19 °C, partially shaded, elevation 1,395 m, 18°49'09.5"N, 98°53'14.3"E), at Doi Pui Temple, Doi Suthep Pui, Muang District, Chiang Mai Province, Thailand, 7-IV-2017, by W. Srisuka (Coll. No. 92).

***Paratypes***: Five females, five males (thorax of one male for DNA analysis) (with their associated pupal exuviae and cocoons), and six mature larvae (two mature larvae for DNA analysis) (in 80% ethanol), same data as for holotype.

##### Diagnosis.

**Figure 18.**
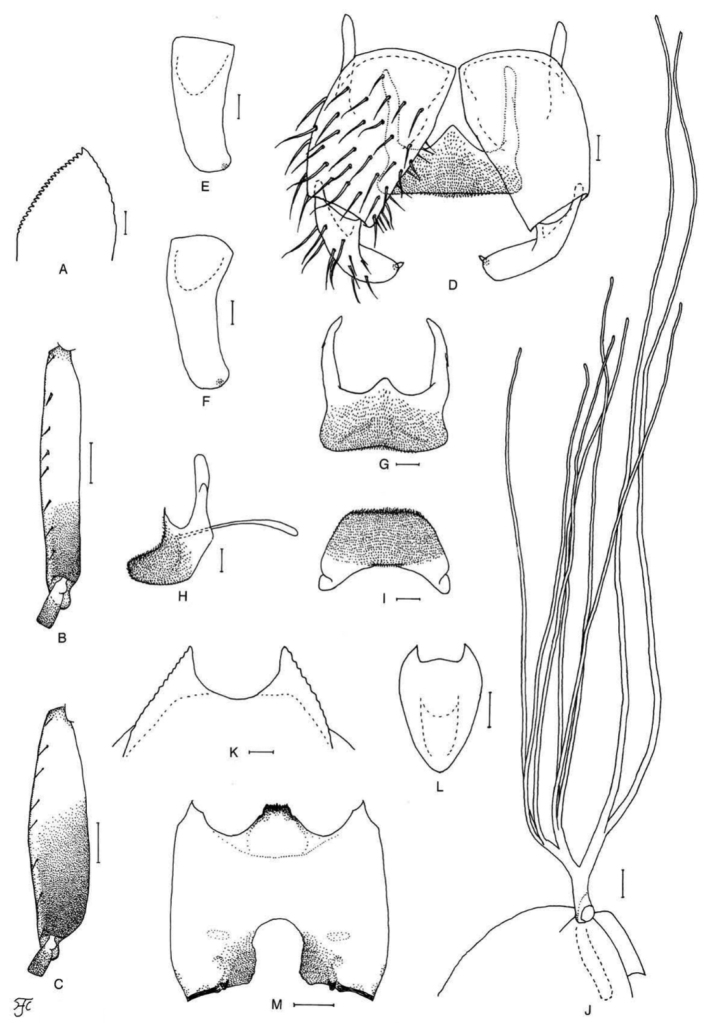
Female, male, pupa and larva of *S. junkumae* sp. nov. **A, B** female **C–I** male **J–L** pupa **M** larva. **A** mandible (right side) **B, C** hind basitarsus and second tarsomere (left side; lateral view **B** female **C** male) **D** coxites, styles and ventral plate (ventral view) **E, F** styles (right side; ventrolateral view) **G** ventral plate (ventral view) **H** ventral plate and median sclerite (lateral view) **I** ventral plate (caudal view) **J** gill filaments (right side; lateral view) **K** terminal hooks (caudal view) **L** cocoon (dorsal view) **M** head capsule (ventral view). Scale bars: 1.0 mm (**L**); 0.1 mm (**B, C, J, M**); 0.02 mm (**D–I**); 0.01 mm (**A, K**).

Female: relatively narrower frons against the head width (1.0:4.7–5.5), and mandible without teeth on the outer margin (Fig. 18A). Male: greater number of upper-eye facets in 15–17 vertical columns and 15–17 horizontal rows on each side. Larva: small number of the primary rays of the labral fan (24 or 25), postgenal cleft as long as or little shorter than the postgenal bridge (Fig. 18M) and abdominal segments 1–3 entirely grey (Fig. 25K).

##### Description.

**Female** (*N* = 5). Body length 2.0–2.3 mm.

***Head.*** Frontal ratio 1.8–1.9:1.0:2.5–3.2; frons:head ratio 1.0:4.7–5.5. Labrum 0.64–0.67 times length of clypeus. Maxillary palpus: proportional lengths of third, fourth, and fifth palpal segments 1.0:1.0–1.1:2.0–2.1; sensory vesicle medium sized, ellipsoidal (0.27–0.32 times length of third palpal segment), with medium opening. Lacinia with ten or eleven inner and 14 or 15 outer teeth. Mandible (Fig. 18A) with 22–26 inner teeth and lacking distinct outer teeth (though outer margin undulated from tip for some short distance appearing to have ca. ten vestigial teeth).

***Legs.*** Foreleg: trochanter dark yellow to light brown; femur light brown with apical cap medium brown (though extreme tip yellowish); basitarsus moderately dilated, 6.7–7.0 times as long as its greatest width. Midleg: trochanter dark yellow except basal half yellow; tarsus dark brown to brownish black except basal one-fourth of basitarsus dark yellow (its border not well defined). Hind leg: tibia yellowish white on little more than basal half and light brown to brownish black on rest; basitarsus (Fig. 18B) 5.9 times as long as wide, and 0.7 and 0.6 times as wide as greatest widths of tibia and femur, respectively; calcipala (Fig. 18B) nearly as long as width at base, and 0.56 times as wide as greatest width of basitarsus.

***Wing.*** Length 2.1–2.3 mm.

***Abdomen.*** Dorsal surface of abdomen medium to dark brown except anterior four-fifths of segment 2 whitish. Ventral surface of segment 2 whitish and those of other segments light to dark brown; sternal plate on segment 7 undeveloped.

***Terminalia.*** Sternite 8 bare medially, with 25–28 medium-long to long hairs together with two or six slender short hairs on each side. Ovipositor valve triangular, thin, membranous, moderately covered with microsetae interspersed with two to four short hairs; inner margins nearly straight or slightly sinuous, somewhat sclerotized, and moderately separated from each other. Paraproct in ventral view with five or six sensilla on anteromedial surface; paraproct in lateral view somewhat produced ventrally beyond ventral tip of cercus, 0.6 times as long as wide, with 25–28 medium-long to long hairs on ventral and lateral surfaces. Cercus in lateral view 0.4–0.5 times as long as wide. Spermatheca ellipsoidal, 1.3 times as long as its greatest width.

**Male** (*N* = 6). Body length 2.2–2.3 mm.

***Head.*** Slightly wider than thorax. Upper eye dark brown, consisting of large facets in 15–17 vertical columns and 15–17 horizontal rows on each side. Antenna: first flagellomere elongate, 1.6–1.8 times length of second. Maxillary palpus: proportional lengths of third, fourth, and fifth palpal segments 1.0:1.0:2.4–2.6; sensory vesicle ellipsoidal, 0.17–0.23 times length of third palpal segment.

***Legs.*** Foreleg: tibia light grey to light brown except basal tip and outer surface of basal two-thirds whitish, and apical one-third dark brown; basitarsus moderately dilated, 7.8–8.1 times as long as its greatest width. Midleg: basitarsus dark brown except basal tip yellow. Hind leg: coxa medium brown; tibia dark brown to brownish black except little more than basal one-third whitish yellow; tarsus (Fig. 18C) brownish black except little less than basal half of basitarsus and basal one-third of second tarsomere yellow; basitarsus (Fig. 18C) 3.7–4.0 times as long as wide, and 0.9 and 1.0 times as wide as greatest width of tibia and femur, respectively; calcipala (Fig. 18C) slightly shorter than basal width, and 0.33 times as wide as greatest width of basitarsus.

***Wing.*** Length 2.2–2.4 mm. Subcosta with 7–12 hairs.

***Genitalia.*** Coxite in ventral view (Fig. 18D) nearly rectangular, 1.7 times as long as its greatest width. Style in ventral view (Fig. 18D) with triangular apex; style in ventrolateral view (Fig. 18E, F) slightly tapered toward apex or nearly parallel-sided from basal one-third to apex, and 0.8 times length of coxite. Ventral plate in ventral view (Fig. 18D) with body transverse, 0.46 times as long as wide, with anterior margin produced anteromedially, posterior margin nearly straight or somewhat concave medially, and lateral margin emarginated medially; basal arms of moderate length, nearly parallel-sided, then convergent apically; ventral plate in caudal view (Fig. 18I) trapezoidal, with ventral margin nearly straight or slightly concave ventrally. Cercus with 14 or 15 hairs.

**Pupa** (*N* = 11). Body length 2.4–2.8. mm.

***Thorax.*** Integument moderately covered with round tubercles except dorsal and dorsolateral surfaces of posterior half sparsely covered with tubercles. Gill (Fig. 18J) composed of eight slender thread-like filaments, arranged as [3+(1+2)]+2 or [3+(2+1)]+2 from dorsal to ventral; common basal stalk 0.7–0.8 times length of interspiracular trunk; stalk of ventral pair of filaments 0.7–1.2 times length of common basal stalk, and 0.5–0.9 times length of interspiracular trunk; primary stalk of dorsal triplet lying against that of lower pair at angle of 60° when viewed laterally; filaments of dorsal and middle triplets subequal in length (2.0–2.6 mm) and thickness to one another; two filaments of ventral pair subequal in length (3.0–3.9 mm) and thickness to each other and 1.6–1.8 times as thick as six other filaments of dorsal and middle triplets when compared basally.

***Abdomen.*** Dorsally, all segments light yellow; segments 1 and 2 without tubercles; segment 9 with pair of wide flat terminal hooks (Fig. 18K), of which outer margin is 2.2–2.6 times length of inner margin and crenulated when viewed caudally.

***Cocoon*** (Fig. 18L). Wall-pocket-shaped, moderately woven, moderately extended ventrolaterally; anterior margin moderately woven medially, rarely with bulge; individual threads visible; 3.3–3.9 mm long by 2.0–2.5 mm wide.

**Mature larva** (*N* = 4). Body length 4.9–5.4 mm. Body creamy white with following color markings: thoracic segment 1 encircled with distinct reddish brown band (though disconnected ventromedially), thoracic segments 2 and 3 ochreous on ventral surface; abdominal segments 1–3 entirely grey, abdominal segment 4 with reddish brown transverse band dorsally (though often partially to completely faded, leaving narrow band or small spot(s) dorsally), abdominal segments 5 and 6 each with distinct reddish brown, W-shaped, transverse band (of which central and dorsolateral parts marked) along posterior margin on dorsal and dorsolateral surfaces (though band on abdominal segment 6 often partially faded, leaving one round dorsomedial spot and two larger dorsolateral lateral spots); abdominal segments 7 and 8 covered with reddish brown pigments on dorsal and dorsolateral surfaces (though central portion often faded to varying extent) (Fig. 25K).

***Head.*** Head capsule yellow except eye-spot region whitish, sparsely covered with minute setae (though moderately on dorsal surface); head spots faintly to moderately positive or indistinct. Antenna: proportional lengths of first, second, and third articles 1.0:0.7–0.8:0.7–0.8. Labral fan with 24 or 25 primary rays. Postgenal cleft (Fig. 18M) rounded or quadrate, 0.9–1.0 times length of postgenal bridge.

***Abdomen.*** Rectal organ compound, each of three lobes with 8–11 finger-like secondary lobules. Anal sclerite: anterior arms 1.0–1.2 times as long as posterior ones. Posterior circlet with 81–84 rows of hooklets with up to 14 hooklets per row.

##### Etymology.

The species name *junkumae* is in honor of Assistant Prof. Dr. Anuluck Junkum, Department of Parasitology, Faculty of Medicine, Chiang Mai University, Thailand, for her kind help in the field and laboratory works of black flies.

##### Distribution.

Thailand (Chiang Mai).

##### Discussion.

This new species is similar to *S. hongthaii* Takaoka, Sofian-Azirun & Ya’cob described from Vietnam (Takaoka et al. 2014a) in having a greater number of male upper-eye facets. However, it is distinguished from the latter species in the female by the narrower frons (frons:head ratio 1:4.7–5.5 in the new species versus 1:4.1–4.2 in *S. hongthaii*), in the male by the subcosta with hairs (subcosta bare in *S. hongthaii*) and in the larva by abdominal segments 1–3 grey (Fig. 25K) (abdominal segments 1–4 greyish in *S. hongthaii*).

The larva of S. sp. nr. asakoae-2 reported from Thailand by Jitklang et al. (2008) has a medium-sized postgenal cleft and abdominal segments 1–3 each with a greenish transverse band, both characters resembling those of this new species, but it differs by lacking distinct color markings on abdominal segments 5–8.

#### Simulium (Gomphostilbia) kiewfinense

Taxon classificationAnimaliaDipteraSimuliidae

Takaoka, Srisuka & Fukuda sp. nov.

B78934D5-1657-51C9-A93B-74F1F41A438A

http://zoobank.org/79DF8917-1F47-47C3-AC8A-6B26E78EAA83

Fig. 19

##### Material examined.

***Holotype***: Male (with its associated pupal exuviae and cocoon) (in 80% ethanol) labeled as “Holotype: *Simulium kiewfinense* male, QSBG col. no. 36, Thailand, 6-IV-2018, by W. Srisuka”, collected from a stream (width 1.1 m, depth 12 cm, bed sandy, moderate flow, pH 6.9, 18.3 °C, partially shaded, elevation 1,446 m, 18°51'38.8"N, 99°22'15.2"E), at Kiew Fin, Muang Pan District, Lampang Province, Thailand, 6-IV-2018, by W. Srisuka (Coll. No. 36).

***Paratypes***: Two males (thorax of one male for DNA analysis) (with their associated pupal exuviae and cocoons) (in 80% ethanol), same data as for holotype.

##### Diagnosis.

Male: greater number of upper-eye facets in 16 or 17 vertical columns and 16 or 17 horizontal rows one each side.

##### Description.

**Male** (*N* = 3). Body length 2.0–2.1 mm.

***Head.*** Somewhat wider than thorax. Upper eye medium brown, consisting of large facets in 16 or 17 vertical columns and 16 or 17 horizontal rows. Antenna composed of scape, pedicel and nine flagellomeres, medium to dark brown except scape, pedicel, and base of first flagellomere yellow; first flagellomere elongate, 1.8 times length of second. Maxillary palpus light brown, with five palpal segments, proportional lengths of third, fourth, and fifth palpomeres 1.0:1.0:2.6; sensory vesicle 0.19 times length of third palpal segment.

**Figure 19.**
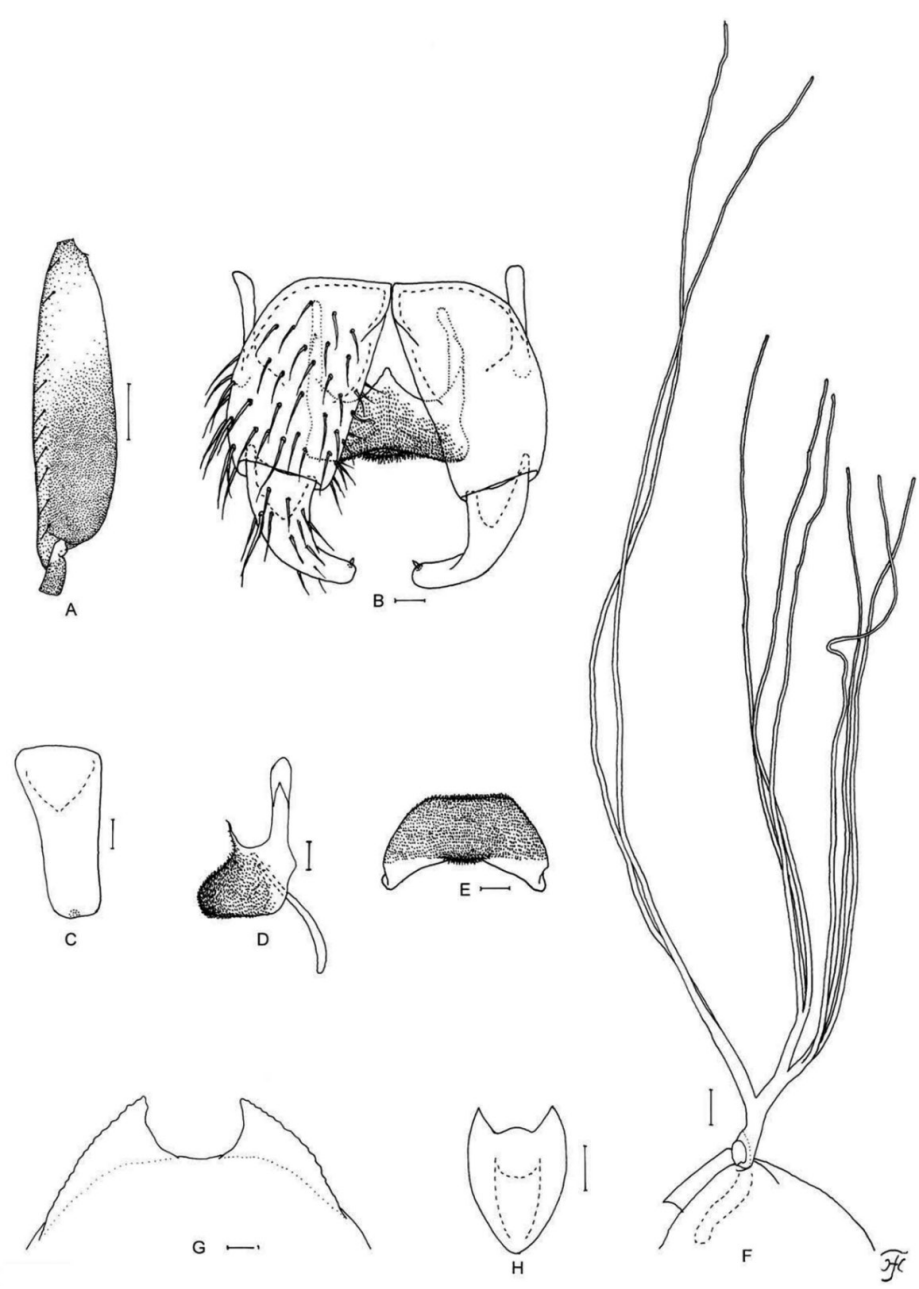
Male and pupa of *S. kiewfinense* sp. nov. **A–E** male **F–H** pupa. **A** hind basitarsus and second tarsomere (left side; lateral view) **B** coxites, styles and ventral plate (ventral view) **C** style (right side; ventrolateral view) **D** ventral plate and median sclerite (lateral view) **E** ventral plate (caudal view) **F** gill filaments (left side; lateral view) **G** terminal hooks (caudal view) **H** cocoon (dorsal view). Scale bars: 1.0 mm (**H**); 0.1 mm (**A, F**); 0.02 mm (**B–E**); 0.01 mm (**G**).

***Legs.*** Foreleg: tibia whitish except apical three-tenths dark brown and subbasal portion light brown; basitarsus moderately dilated, 6.6–7.1 times as long as its greatest width. Midleg: tarsus dark brown except basal one-third of basitarsus dark yellow to light brown (border not well defined). Hind leg: coxa light brown; tarsus (Fig. 19A) brownish black except basal two-fifths to little less than basal half of basitarsus and basal one-third of second tarsomere yellow; basitarsus (Fig. 19A) 3.7–3.8 times as long as wide, and 0.9 and 1.0 times as wide as greatest width of tibia and femur, respectively; calcipala (Fig. 19A) slightly shorter than basal width, and 0.33 times as wide as greatest width of basitarsus.

***Wing.*** Length 1.9–2.0 mm. Subcosta bare.

***Genitalia.*** Coxite in ventral view (Fig. 19B) nearly rectangular, 1.8 times as long as its greatest width. Style in ventral view (Fig. 19B) with round apex. Ventral plate in ventral view (Fig. 19B) with basal arms nearly parallel-sided, then convergent apically; ventral plate in caudal view (Fig. 19E) with ventral margin nearly straight. Cercus with 14 or 15 hairs.

**Pupa** (*N* = 3). Body length 2.5–2.7 mm.

***Head.*** Integument yellow.

***Thorax.*** Integument yellow, moderately covered with round tubercles except dorsolateral surface of posterior half almost bare. Gill (Fig. 19F) composed of eight slender thread-like filaments, arranged as [3+(1+2)]+2 from dorsal to ventral; common basal stalk 0.7–0.8 times length of interspiracular trunk; stalk of ventral pair of filaments 0.9–1.2 times length of common basal stalk, and 0.7–0.9 times length of interspiracular trunk; primary stalk of dorsal triplet lying against that of lower pair at angle of 70–80° when viewed laterally; filaments of dorsal triplet subequal in length (1.5–2.1 mm) and thickness to one another; filaments of middle triplet subequal in length (2.0–2.5 mm) and thickness to one another; two filaments of ventral pair subequal in length (3.0–3.5 mm) and thickness to each other and 1.3–1.5 times as thick as six other filaments of dorsal and middle triplets when compared basally.

***Abdomen.*** Dorsally, all segments light yellowish; segments 1 and 2 without minute tubercles; segment 9 with pair of wide flat terminal hooks (Fig. 19G), of which outer margin 2.3–2.7 times length of inner margin and crenulated when viewed caudally.

***Cocoon*** (Fig. 19H). Pale yellow, slipper-shaped, moderately woven, moderately extended ventrolaterally; anterior margin thickly woven medially, often with bulge; 3.0–3.3 mm long by 2.2 mm wide.

**Female** and **mature larva**. Unknown.

##### Etymology.

The species name, *kiewfinense*, refers to the local name, Kiew Fin, where this species was collected.

##### Distribution.

Thailand (Lampang).

##### Discussion.

This new species is similar to *S. junkumae* sp. nov. described above and *S. hongthaii* from Vietnam (Takaoka et al. 2014a) in having the greater number of male upper-eye facets. However, it is distinguished from both species by the relative length of the fore basitarsus against its greatest width (6.6–7.1 in this new species versus 7.8–8.1 in *S. junkumae* sp. nov. and 7.5–8.4 in *S. hongthaii*), also from *S. junkumae* sp. nov. by the male subcosta bare (subcosta with hairs in *S. junkumae* sp. nov.) and from S. (G.) hongthaii by the relative length of the coxite against its greatest width (1.8 in this new species and 2.2 in *S. hongthaii*).

#### Simulium (Gomphostilbia) huaimorense

Taxon classificationAnimaliaDipteraSimuliidae

Takaoka, Srisuka & Saeung sp. nov.

CF5CA013-D850-5B9D-93B4-F2DBA0939663

http://zoobank.org/2B1BBBD1-655B-4567-B20E-F96954413D2D

Figs 20 , 25I

##### Material examined.

***Holotype*.** Male (with its associated pupal exuviae and cocoon) (in 80% ethanol) labeled as “Holotype: *Simulium huaimorense* male, QSBG col. no. 49, Thailand, 2-II-2019, by W. Srisuka”, collected from a stream (width 1.2 m, depth 10 cm, bed sandy, moderate flow, pH 6.6, 17.1 °C, exposed to the sun, elevation 1,154 m, 19°03'36.8"N, 99°19'15.7"E), at a coffee plantation, Huai Mor, Doi Saket, Chiang Mai Province, Thailand, 2-II-2019, by W. Srisuka (Coll. No. 49).

***Paratypes*.** One female, two males (thorax of one male for DNA analysis) (with its associated pupal exuviae and cocoon) (in 80% ethanol), and three mature larvae (in 80% ethanol), collected from a small stream (width 30 cm, depth 2 cm, bed sandy, moderate flow, pH 7.3, 19.8 °C, partially shaded, elevation 1,440 m, 19°54'04.1"N, 99°34'26.6"E), at coffee plantation, Pha Lung Village, Muang District, Chiang Rai Province, northern Thailand, 30-VIII-2018, by W. Srisuka (Coll. No. 122).

##### Diagnosis.

**Figure 20.**
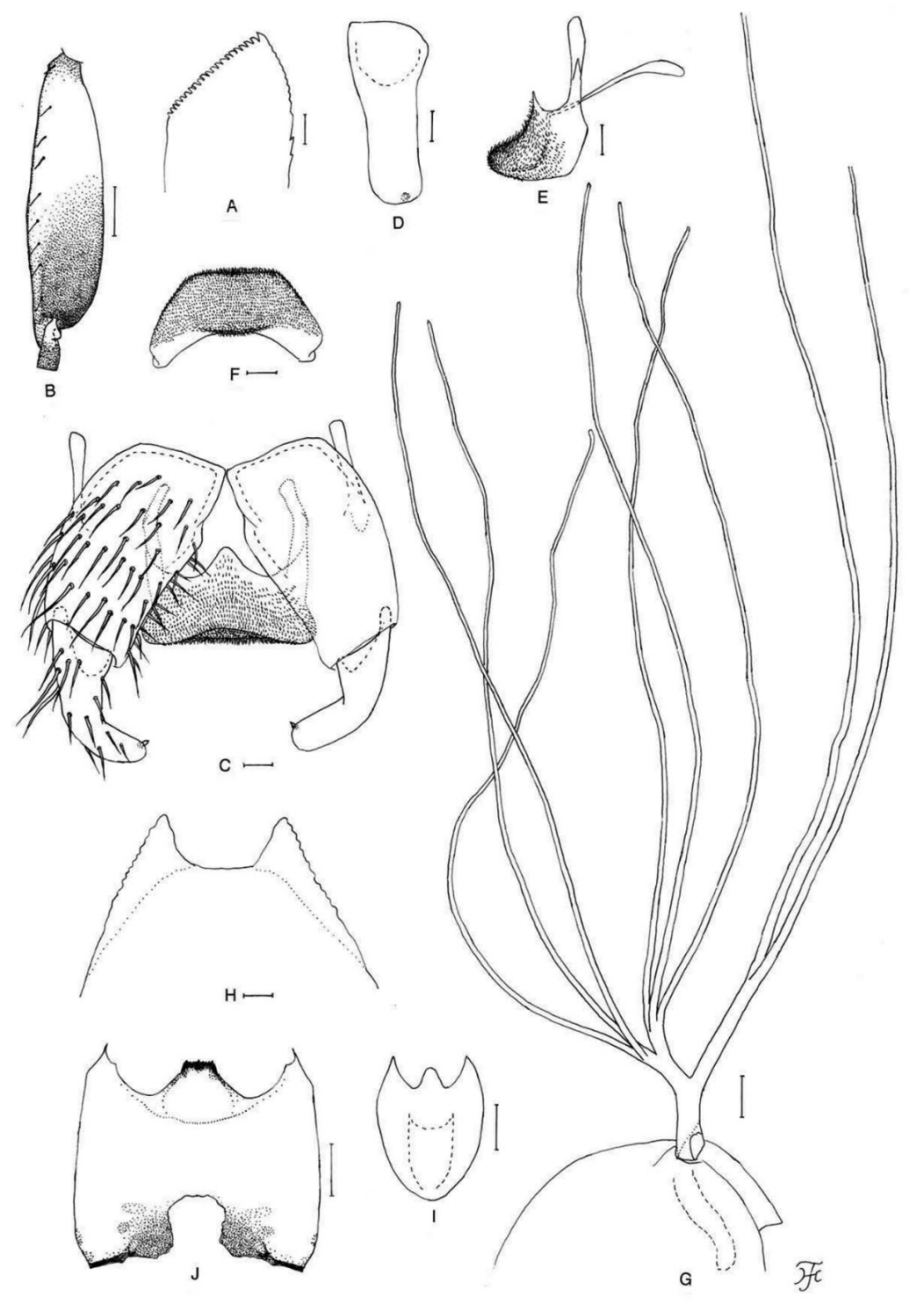
Female, male, pupa and larva of *S. huaimorense* sp. nov. **A** female **B–F** male **G–I** pupa **J** larva. **A** mandible (right side) **B** hind basitarsus and second tarsomere (left side; lateral view) **C** coxites, styles and ventral plate (ventral view) **D** style (right side; ventrolateral view) **E** ventral plate and median sclerite (lateral view) **F** ventral plate (caudal view) **G** gill filaments (right side; lateral view) **H** terminal hooks (caudal view) **I** cocoon (dorsal view) **J** head capsule (ventral view). Scale bars: 1.0 mm (**I**); 0.1 mm (**B, G, J**); 0.02 mm (**C–F**); 0.01 mm (**A, H**).

Male: upper-eye large facets in 16 vertical columns and 17 horizontal rows. Pupa: extremely short primary stalks of the dorsal and middle triplets of filaments (Fig. 20G) and cocoon with a short anterodorsal projection. Larva: abdominal segments 1–4 grey.

##### Description.

**Female** (*N* = 1). Body length 2.0 mm.

***Head.*** Frontal ratio 1.9:1.0:2.6; frons:head ratio 1.0:4.8. Labrum 0.67 times length of clypeus. Maxillary palpus: proportional lengths of third, fourth, and fifth palpal segments 1.0:1.2:2.3; sensory vesicle ellipsoidal, medium long (0.27–0.28 times length of third palpal segment). Lacinia with nine or ten inner and 14 or 15 outer teeth. Mandible (Fig. 20A) with 20 inner teeth and one or two outer teeth at some distance from tip.

***Legs.*** Foreleg: Basitarsus moderately dilated, 6.5 times as long as its greatest width. Hind leg: coxa light brown; tibia yellowish white on basal two-thirds and light brown to brownish black on rest; basitarsus 6.2 times as long as wide, and 0.7 and 0.6 times as wide as greatest widths of tibia and femur, respectively; calcipala nearly as long as width at base, and 0.6 times as wide as greatest width of basitarsus; claw with large basal tooth 0.46 times length of claw.

***Wing.*** Length 2.0 mm.

***Abdomen.*** Dorsal surface of abdomen medium to dark brown except anterior five-sixths of segment 2 ochreous

***Terminalia.*** Sternite 8 bare medially, with 23 or 24 medium-long to long hairs together with three or four slender short hairs on each side. Ovipositor valve moderately covered with microsetae interspersed with two or three short hairs; inner margins nearly straight or slightly sinuous, Paraproct with four sensilla on anteromedial surface; paraproct in lateral view 0.6 times as long as wide, with 24 or 25 medium-long to long hairs on ventral and lateral surfaces. Cercus in lateral view 0.5 times as long as wide. Spermatheca ellipsoidal, 1.3 times as long as its greatest width.

**Male** (*N* = 3). Body length 2.0 mm.

***Head.*** Slightly wider than thorax. Upper eye dark brown, consisting of large facets in 16 vertical columns and 17 horizontal rows on each side. Antenna: first flagellomere elongate, 1.9 times length of second. Maxillary palpus: proportional lengths of third, fourth, and fifth palpal segments 1.0:1.2:2.2; sensory vesicle small, ellipsoidal, 0.22 times length of third palpal segment.

***Legs.*** Foreleg: coxa whitish yellow; trochanter light brown; femur light brown except apical tip yellowish; tibia whitish yellow except inner and lateral surface of basal one-fifth light brown and little more than apical one-third dark brown; basitarsus 7.0–8.0 times as long as its greatest width. Midleg: trochanter light brown except base yellow; femur light to medium brown with base yellowish and apical cap dark brown (though apical tip yellow). Hind leg: coxa light brown except apical one-third whitish yellow; femur medium to dark brown with basal tip yellow and apical cap brownish black (though apical tip yellow); tibia dark brown to brownish black except little less than basal half whitish yellow and subbasal small portion somewhat dark yellow; tarsus (Fig. 20B) brownish black except little less than basal half of basitarsus (though its border not well defined) and basal one-third of second tarsomere whitish yellow; basitarsus (Fig. 20B) 3.6–4.0 times as long as wide, and 0.9–1.1 and 1.0–1.1 times as wide as greatest width of tibia and femur, respectively; calcipala (Fig. 20B) 0.31 times as wide as greatest width of basitarsus.

***Wing.*** Length 2.0 mm. Subcosta bare or with two hairs.

***Abdomen.*** Ventral surface of segment 2 white, those of segments 3 and 4 light brown although sternal plates medial brown, and those of other segments medium to dark brown.

***Genitalia.*** Coxite in ventral view (Fig. 20C) nearly rectangular, 1.8 times as long as its greatest width. Style in ventral view (Fig. 20C) with round apex; and in ventrolateral view (Fig. 20D) tapered from base to basal two-fifths, then nearly parallel-sided, with round apex. Ventral plate in ventral view (Fig. 20C) with posterior margin somewhat concave medially, and lateral margin emarginated medially; basal arms of moderate length, slightly divergent, then convergent apically; ventral plate in caudal view (Fig. 20F) trapezoidal, with ventral margin nearly straight medially. Cercus with 12–16 hairs.

**Pupa** (*N* = 4). Body length 2.5 mm.

***Thorax.*** Integument yellow, moderately covered with round tubercles except dorsal and dorsolateral surface of posterior half sparsely covered with tubercles. Gill (Fig. 20G) composed of eight slender thread-like filaments, arranged as [3+(1+2)]+2from dorsal to ventral; common basal stalk 0.7 times length of interspiracular trunk; dorsal and middle triplets sharing short stalk, and dorsal triplet composed of three individual filaments arising at same level, middle triplet composed of one individual and two paired filaments with extremely short secondary stalk; stalk of ventral pair of filaments medium-long, 1.4 times length of common basal stalk, and nearly as long as interspiracular trunk; primary stalks of dorsal and middle triplets extremely short, 0.3 and 0.6 times length of their common stalk, respectively; primary stalk of dorsal triplet lying against that of lower pair at angle of 90° when viewed laterally; filaments of dorsal and middle triplets subequal in length (2.1–2.3 mm) and thickness to one another; two filaments of ventral pair subequal in length (3.0–3.5 mm) and thickness to each other, and 1.3–1.4 times as thick as six other filaments of dorsal and middle triplets when compared basally; all filaments light brown.

***Abdomen.*** Dorsally, all segments light yellowish; segments 1 and 2 without minute tubercles; segment 9 with pair of wide flat terminal hooks (Fig. 20H), of which outer margin 2.9 times length of inner margin and crenulated when viewed caudally.

***Cocoon*** (Fig. 20I). Light yellow, slipper-shaped, moderately woven, widely extended ventrolaterally; anterior margin thickly woven medially, with short projection; individual threads not visible; 3.5 mm long by 2.5 mm wide.

**Mature larva** (*N* = 3). Body length 4.5–5.3 mm. Body creamy white with following color markings: thoracic segment 1 encircled with distinct reddish brown band (though disconnected ventromedially), thoracic segments 2 and 3 ochreous on ventral surface; abdominal segments 1–4 entirely grey, abdominal segments 7 and 8 light grey on dorsal and dorsolateral surfaces; abdominal segments 5 and 6 each with distinct reddish brown, W-shaped, transverse band (of which central and dorsolateral parts marked) along posterior margin of dorsal and dorsolateral surfaces (though band on abdominal segment 6 often partially faded, leaving one round dorsomedial spot and two larger dorsolateral lateral spots); abdominal segments 7 and 8 covered with reddish brown pigments on dorsal and dorsolateral surfaces (though central portion often faded out to varying extent) (Fig. 25I); abdominal segments 5–7 each with pair of reddish brown spots ventrally (though often faded).

***Head.*** Head capsule yellow except eye-spot region whitish, sparsely covered with minute setae (though moderately on dorsal surface); head spots faintly to moderately positive. Antenna: proportional lengths of first, second, and third articles 1.00:0.72–0.75:0.75–0.82. Labral fan with 24–26 primary rays. Postgenal cleft (Fig. 20J) rounded or slightly triangular anteriorly, 0.8–1.0 times length of postgenal bridge.

***Abdomen.*** Rectal organ compound, each of three lobes with 11–13 finger-like secondary lobules. Anal sclerite: anterior arms nearly as long as or slightly longer than posterior ones. Posterior circlet with 86–92 rows of hooklets with up to 14 hooklets per row.

##### Etymology.

The species name, *huaimorense*, refers to the locality name, Huai Mor, where this species was collected.

##### Distribution.

Thailand (Chiang Mai and Chiang Rai).

##### Discussion.

*Simulium huaimorense* sp. nov. is similar to *S. myanmarense* described from Myanmar (Takaoka et al. 2017b) in that both species share the high number of male upper-eye large facets and cocoon with a short anterodorsal projection (Fig. 20I). However, this new species is distinguished in the male by the upper-eye large facets in 16 vertical columns and 17 horizontal rows (14 or 15 vertical columns and 15 or 16 horizontal rows in *S. myanmarense*), relative length of the hind basitarsus against its greatest width, which is 3.6–4.0 in this new species versus 4.3–4.4 in *S. myanmarense*, in the pupa by the extremely short primary stalks of the dorsal and middle triplets of filaments (Fig. 20G), which are 0.1–0.3 and 0.6–0.7 times as long as their common stalk (the dorsal and middle primary stalks are nearly as long as their common stalk in *S. myanmarense*), and in the larva by abdominal segments 1–4 grey (abdominal segments 1 and 2 grey in *S. myanmarense*).

This new species is distinguished from *S. maewongense* sp. nov. and four other related species (noted under *S. maewongense* sp. nov.), which have a cocoon with a short anterodorsal projection (Fig. 20I), by the higher number of the male upper-eye large facets.

This new species is similar in the higher number of male upper-eye facets and the arrangement of the pupal gill filaments to *S. kiewfinense* sp. nov., and *S. junkumae* sp. nov. from Thailand and *S. hongthaii* from Vietnam (Takaoka et al. 2014a) but is distinguished from the latter three species by the cocoon with a short anterodorsal projection, and from *S. junkumae* sp. nov. by larval abdominal segments 1–4 being grey (Fig. 25I) (larval abdominal segments 1–3 grey in *S. junkumae* sp. nov.).

This new species is distinguished from *S. doisaketense* from Doi Saket, Chiang Mai Province (Jitklang et al. 2008), by the primary stalks of the dorsal and middle triplets of gill filaments much shorter than their common stalk (much longer in *S. doisaketense*).

#### Simulium (Gomphostilbia) songense

Taxon classificationAnimaliaDipteraSimuliidae

Takaoka, Srisuka & Fukuda sp. nov.

C7E6ED9A-1891-5FEF-9C57-4AA9F27BD8C2

http://zoobank.org/C45AE92B-5925-45D9-B64F-E3DC2BF7D170

Fig. 21

##### Material examined.

***Holotype***: Female (thorax for DNA analysis) (with its associated pupal exuviae and cocoon) (in 80% ethanol) labeled as “Holotype: *Simulium songense* male, QSBG col. no. 60, Thailand, 25-VII-2017, by W. Srisuka”, collected from a small stream (width 80 cm, depth 2.5, fast flow, pH 7.2, 20.1 °C, partially shaded, elevation 1,157 m, 19°11'10.3"N, 101°04'41.7"E), at Nam Dan Village, Pua District, Nan Province, northern Thailand, 25-VII-2017, by W. Srisuka (Coll. No. 60).

***Paratype***: One female (thorax for DNA analysis) (with its associated pupal exuviae and cocoons) (in 80% ethanol), collected from a small stream (width 40 cm, depth 3.5 cm, bed sandy, moderate flow, pH 7.36, 21.9 °C, partially shaded, elevation 582 m, 18°45'30.2"N, 100°20'11.4"E), at Chao Wa Waterfall, Song District, Phrae Province, Thailand, 16-III-2017, by W. Srisuka (Coll. No.164).

##### Diagnosis.

**Figure 21.**
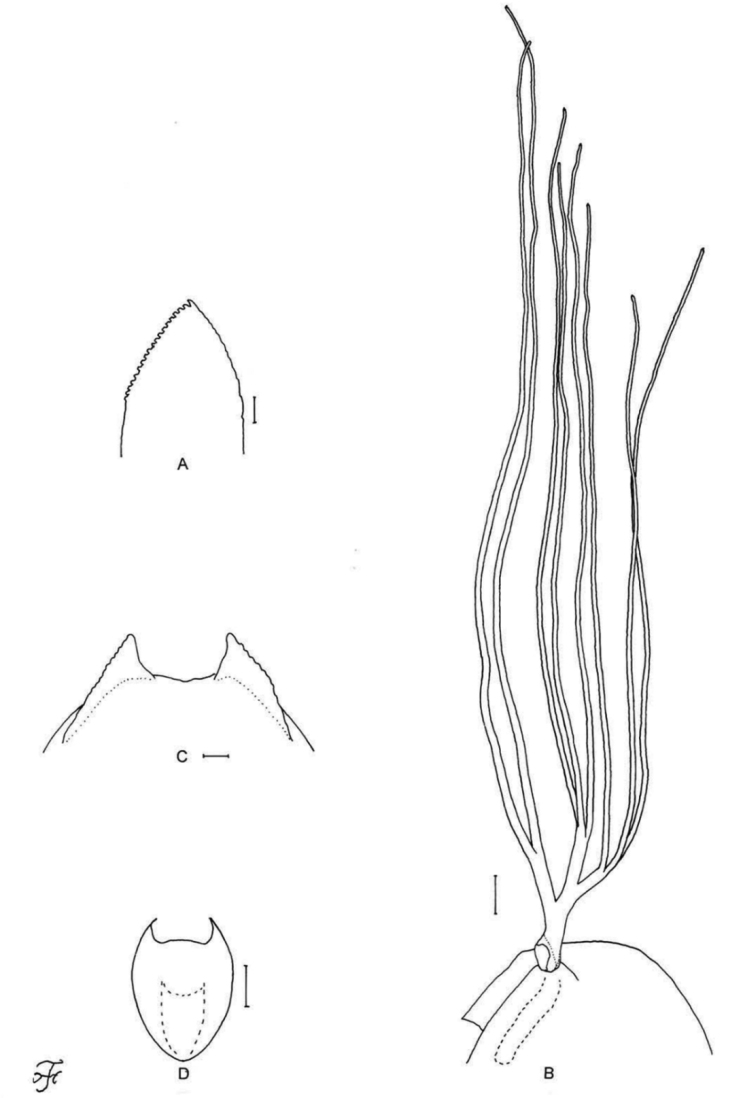
Female and pupa of *S. songense* sp. nov. **A** female **B–D** pupa. **A** mandible (right side) **B** gill filaments (left side; lateral view) **C** terminal hooks (caudal view) **D** cocoon (dorsal view). Scale bars: 1.0 mm (**D**); 0.1 mm (**B**); 0.01 mm (**A, C**).

Female: mandible without distinct teeth on the outer margin (Fig. 21A). Pupa: short stalk of the ventral pair of gill filaments being half the length of the interspiracular trunk (Fig. 21B).

##### Description.

**Female** (*N* = 2). Body length 1.9 mm.

***Head.*** Frontal ratio 1.9:1.0:2.6; frons:head ratio 1.0:4.9. Labrum 0.65 times length of clypeus. Maxillary palpus: proportional lengths of third, fourth, and fifth palpal segments 1.0:1.1:2.6; sensory vesicle 0.26–0.29 times length of third palpal segment. Lacinia with nine or ten inner and 13 outer teeth. Mandible (Fig. 21A) with 19 or 20 inner teeth and lacking distinct teeth, though outer margin undulated, appearing to have three weakly developed teeth at some distance from tip.

***Legs.*** Foreleg: basitarsus moderately dilated, 5.5 times as long as its greatest width. Midleg: tarsus dark brown to brownish black though basal half of basitarsus dark yellow or light brown (its border not well defined). Hind leg: coxa light brown); tibia yellowish white on basal two-thirds and light brown to brownish black on rest; tarsus brownish black except basal seven-tenths (though base light brown) and basal half of second tarsomere yellowish white; basitarsus 6.2 times as long as wide, and 0.7 and 0.5 times as wide as greatest widths of tibia and femur, respectively; calcipala nearly as long as width at base, and 0.6 times as wide as greatest width of basitarsus; claw with large basal tooth 0.46 times length of claw.

***Wing.*** Length 2.0 mm.

***Abdomen.*** Dorsal surface of abdomen medium to dark brown except most of segment 2 ochreous (though narrowly darkened along posterior margin).

***Terminalia.*** Sternite 8 bare medially, with 28– 31 medium-long to long hairs together with three or four slender short hairs on each side. Ovipositor valves each moderately covered with microsetae interspersed with three to four short hairs. Paraproct in ventral view with four or five sensilla on anteromedial surface; paraproct in lateral view 0.5 times as long as wide, with 29–32 medium-long to long hairs on ventral and lateral surfaces. Cercus in lateral view 0.5 times as long as wide.

**Pupa** (*N* = 2). Body length 3.0 mm.

***Head.*** Integument yellow.

***Thorax.*** Integument yellow, moderately covered with round tubercles except dorsolateral surface of posterior half sparsely covered with tubercles. Gill (Fig. 21B) composed of eight slender thread-like filaments, arranged as [3+(1+2)]+2 from dorsal to ventral; common basal stalk 0.7–0.8 times length of interspiracular trunk; stalk of ventral pair of filaments 0.6–0.8 times length of common basal stalk, and 0.5 times length of interspiracular trunk; primary stalk of dorsal triplet lying against that of lower pair at angle of 70° when viewed laterally; filaments of dorsal triplet subequal in length (1.8–2.0 mm) and thickness to one another; filaments of middle triplet subequal in length (2.1–2.2 mm) and thickness to one another; two filaments of ventral pair subequal in length (2.4–2.5 mm) and thickness to each other and 1.4–1.7 times as thick as six other filaments of dorsal and middle triplets when compared basally; all filaments yellow to light brown, gradually tapered toward apex; cuticle of all filaments with well-defined annular ridges and furrows though becoming less marked apically, densely covered with minute tubercles.

***Abdomen.*** Dorsally, all segments light yellowish; segments 1 and 2 without tubercles; segment 9 with pair of wide flat terminal hooks (Fig. 21C), of which outer margin 2.5–2.6 times length of inner margin and crenulated when viewed caudally.

***Cocoon*** (Fig. 21D). Pale whitish yellow, slipper-shaped, moderately woven, widely extended ventrolaterally; anterior margin thickly woven medially, without bulge or short projection; individual threads visible only on peripheral portions; 3.4 mm long by 2.4 mm wide.

**Male** and **mature larva.** Unknown.

##### Etymology.

The species name, *songense*, refers to the district, Song, one of the two localities where this species was collected.

##### Distribution.

Thailand (Nan and Phrae).

##### Discussion.

Lacking teeth on the outer margin of the mandible, the female of this new species seems to be most similar to *S. myanmarense* and *S. monglaense* from Myanmar (Takaoka et al. 2017b), but is distinguished from the latter two species by the relative length of the fore basitarsus against its greatest width (5.5 in this new species versus 6.0 in *S. myanmarense* and 6.7 in *S. monglaense*).

#### Simulium (Gomphostilbia) klonglanense

Taxon classificationAnimaliaDipteraSimuliidae

Takaoka, Srisuka & Saeung sp. nov.

A6BD5EE4-5AD9-5D4D-9D1B-5BA8DA4D8682

http://zoobank.org/1D6E82E6-C730-4C82-9809-6F526A9851FB

Figs 22 , 25E

##### Material examined.

***Holotype***: Male (with its associated pupal exuviae and cocoon) (in 80% ethanol) labeled as “Holotype: *Simulium klonglanense* male, QSBG col. no. 144, Thailand, 27-VI-2013, by W. Srisuka”, collected from a stream of Klong Nam Lai (width 1.4 m, depth 13 cm, bed sandy, moderate flow, pH 6.2, 25.8 °C, exposed to the sun, elevation 196 m, 16°12'28.3"N, 99°15'47.8"E), at Klong Lan District, Kham Phaeng Phet Province, Thailand, 27-VI-2013, by W. Srisuka (Coll. No. 144).

***Paratypes***: Three females, one male (thorax for DNA analysis) (with their associated pupal exuviae and cocoons), and eight mature larvae (in 80% ethanol), same data as for holotype.

##### Diagnosis.

**Figure 22.**
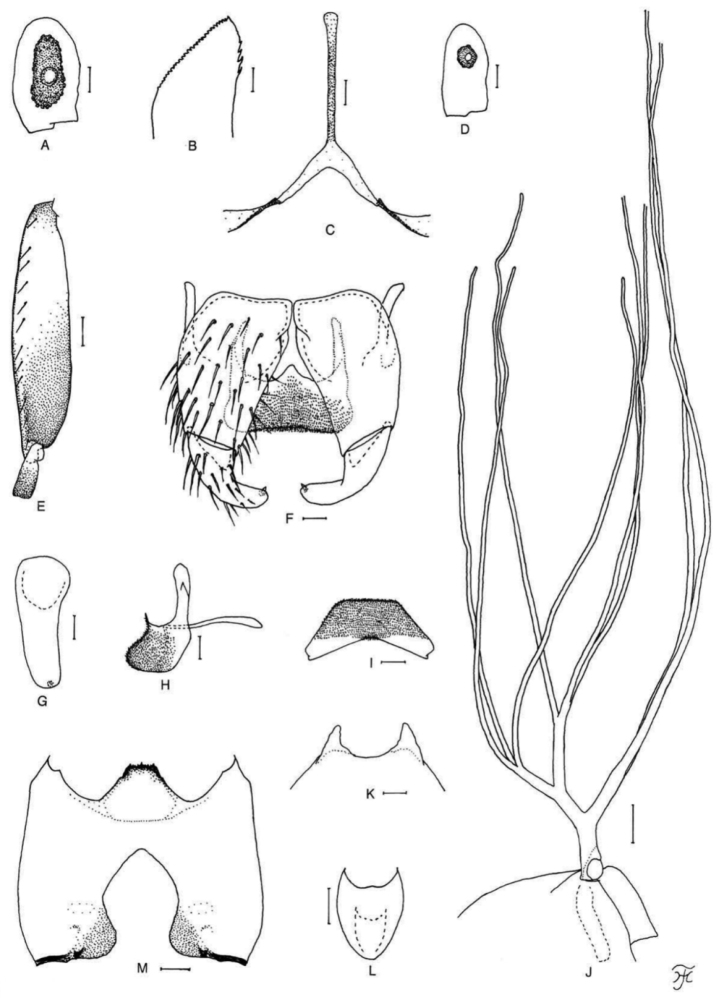
Female, male, pupa and larva of *S. klonglanense* sp. nov. **A–C** female **D–I** male **J–L** pupa **M** larva. **A, D** sensory vesicles (right side; anterior view **A** female **D** male) **B** mandible (right side) **C** genital fork **E** hind basitarsus and second tarsomere (left side; lateral view) **F** coxites, styles and ventral plate (ventral view) **G** style (right side; ventrolateral view) **H** ventral plate and median sclerite (lateral view) **I** ventral plate (caudal view) **J** gill filaments (right side; lateral view) **K** terminal hooks (caudal view) **L** cocoon (dorsal view) **M** head capsule (ventral view). Scale bars: 1.0 mm (**L**); 0.1 mm (**E, J, M**); 0.02 mm (**A, C, D, F–I**); 0.01 mm (**B, K**).

Female: sensory vesicle elongated (Fig. 22A) and mandible with four distinct teeth on the outer margin (Fig. 22B). Male: upper-eye (large) facets in 13 or 14 vertical columns and 14 or 15 horizontal rows on each side, and moderately widened hind basitarsus 0.9 times as wide as the hind tibia and femur. Pupa: small terminal hooks (Fig. 22K). Larva: postgenal cleft 3.7–4.0 times as long as the postgenal bridge (Fig. 22M) and abdominal segments 1–4 grey (Fig. 25E).

##### Description.

**Female** (*N* = 3). Body length 1.8 mm.

***Head.*** Frontal ratio 1.8:1.0:2.2; frons:head ratio 1.0:4.2. Labrum 0.59 times length of clypeus. Maxillary palpus: proportional lengths of third, fourth, and fifth palpal segments 1.0:1.0:2.1; sensory vesicle (Fig. 22A) elongated (0.65–0.68 times length of third palpal segment). Lacinia with 10 inner and 14–16 outer teeth. Mandible (Fig. 22B) with 27 inner teeth and four outer teeth at some distance from tip.

***Legs.*** Foreleg: basitarsus moderately dilated, 5.2–5.4 times as long as its greatest width. Midleg: tarsus light to medium brown though little less than basal half of basitarsus yellow (its border not well defined). Hind leg: coxa light brown; tibia yellowish white on basal two-thirds and light to dark brown on rest; tarsus medium brown except basal two-thirds (though base light brown) and basal half of second tarsomere yellowish white; basitarsus 5.8–6.6 times as long as wide, and 0.7 and 0.6 times as wide as greatest widths of tibia and femur, respectively.

***Wing.*** Length 1.6 mm.

***Abdomen.*** Dorsal surface of abdomen medium to dark brown except most of segment 2 ochreous (though narrow portion along posterior margin somewhat darkened).

***Terminalia.*** Sternite 8 with 17 medium-long to long hairs together with four slender short hairs on each side. Genital fork (Fig. 22C) of usual inverted-Y form, with slender stem; inner margins of arms divergent from each other. Paraproct in ventral view with 3–5 sensilla on anteromedial surface; paraproct in lateral view 0.6 times as long as wide, with 16 medium-long to long hairs on ventral and lateral surfaces. Cercus in lateral view short, rounded posteriorly, 0.5 times as long as wide. Spermatheca 1.44 times as long as its greatest width; both accessory ducts slender, subequal in diameter to each other and slightly wider than major one.

**Male** (*N* = 2). Body length 2.0 mm.

***Head.*** Somewhat wider than thorax. Upper eye medium brown, consisting of large facets in 13 or 14 vertical columns and 14 or 15 horizontal rows on each side. Antenna: first flagellomere elongate, 1.7 times length of second. Maxillary palpus: proportional lengths of third, fourth, and fifth palpal segments 1.0:1.2:2.7; sensory vesicle (Fig. 22D) small, ellipsoidal (0.25–0.27 times length of third palpal segment).

***Thorax.*** Scutum medium to dark brown except anterolateral calli ochreous, with three dark-brown longitudinal vittae (one medial and two submedial), white pruinose except three longitudinal vittae non-pruinose when illuminated anterodorsally and viewed dorsally. Scutellum medium brown. Postnotum medium to dark brown.

***Legs.*** Foreleg: basitarsus slightly dilated, 6.8 times as long as its greatest width. Hind leg: coxa light brown; tibia dark brown except little less than basal half whitish yellow; tarsus (Fig. 22E) medium brown except little less than basal half of basitarsus and little less than basal half of second tarsomere whitish yellow; basitarsus (Fig. 22E) enlarged, 4.2 times as long as wide, and 0.9 and 0.9 times as wide as greatest width of tibia and femur, respectively; calcipala (Fig. 22E) slightly shorter than basal width, and 0.21 times as wide as greatest width of basitarsus.

***Wing.*** Length 1.5 mm. Subcosta with 0–2 hairs.

***Genitalia.*** Style in ventral view (Fig. 22F) bent inward, with round apex having single spine; style in ventrolateral view (Fig. 22G) slightly tapered toward apex, with round apex. Ventral plate in ventral view (Fig. 22F): basal arms of moderate length, nearly parallel-sided, then slightly convergent apically; ventral plate in caudal view (Fig. 22I) trapezoidal, with ventral margin nearly straight. Cercus with 12 or 13 hairs.

**Pupa** (*N* = 5). Body length 2.0–2.4 mm.

***Head.*** Integument light yellow.

***Thorax.*** Integument light yellow, moderately covered with round tubercles except dorsolateral surface of posterior one-third nearly bare. Gill (Fig. 22J) composed of eight slender thread-like filaments, arranged as [3+(1+2)]+2 or [(2+1)+(1+2)]+2 or [(2+1)+3]+2) from dorsal to ventral; common basal stalk 0.7–0.8 times length of interspiracular trunk; stalk of ventral pair of filaments variable in length, 0.6–1.2 times length of common basal stalk, and 0.5–0.9 times length of interspiracular trunk; primary stalk of dorsal triplet lying against that of lower pair at angle of 80–90° when viewed laterally; filaments of dorsal triplet subequal in length (1.8 mm) and thickness to one another; filaments of dorsal triplet subequal in length (1.9–2.1 mm) and thickness to one another; two filaments of ventral pair subequal in length (tips of filaments of ventral pair lost, thus not possible to measure their lengths, which are probably little more than 2.4 mm) and thickness to each other and 1.6–1.8 times as thick as six other filaments of dorsal and middle triplets when compared basally; all filaments pale yellow.

***Abdomen.*** Dorsally, all segments unpigmented except segment 9 light yellow; segments 1 and 2 without minute tubercles; segment 9 with pair of small flat terminal hooks (Fig. 22K) when viewed caudally.

***Cocoon*** (Fig. 22L). Whitish yellow to medium brown, slipper-shaped, moderately woven, moderately extended ventrolaterally; anterior margin moderately woven medially, with or without bulge; individual threads visible or not visible; 2.4–3.0 mm long by 1.7–2.1 mm wide.

**Mature larva** (*N* = 8). Body length 3.3–4.0 mm. Body with following color markings: thoracic segment 1 encircled with grey (or greyish ochreous) band (though disconnected ventromedially), thoracic segments 2 and 3 grey or ochreous on ventral surface; abdominal segments 1–4 each encircled with greyish band, abdominal segments 5–8 greyish on dorsal and dorsolateral surfaces; abdominal segment 4 with or without reddish purplish transverse band (though often partially faded medially and ventrally), abdominal segments 5–8 each faintly to moderately overlaid with reddish purplish pigments dorsally and dorsolaterally (though faded medially to various extent), abdominal segments 5 and 6 each with pair of small grey or reddish purplish spots ventrally, and abdominal segment 7 with grey transverse band (overlaid with reddish purplish pigment) ventrally (Fig. 25E).

***Head.*** Head capsule yellow except eye-spot region whitish; head spots indistinct. Antenna: proportional lengths of first, second, and third articles 1.0:0.8:0.8–1.0. Labral fan with 30–34 primary rays. Postgenal cleft (Fig. 22M) long, arrowhead shaped, 3.7–4.0 times length of postgenal bridge.

***Abdomen.*** Rectal organ compound, each of three lobes with six to eight finger-like secondary lobules. Anal sclerite of usual X-form, with anterior arms nearly as long as posterior ones. Posterior circlet with 71–74 rows of hooklets with up to 14 hooklets per row.

##### Etymology.

The species name, *klonglanense*, refers to the district, Klong Lan, where this species was collected.

##### Distribution.

Thailand (Kham Phaeng Phet).

##### Discussion.

This new species is similar to *S. lurauense* described from Peninsular Malaysia (Takaoka et al. 2011b) in many characters including the elongate female sensory vesicle, presence of teeth on the outer margin of the female mandible, and similar number of male upper-eye facets. However, it is distinguished from the latter species by the relative length of the female sensory vesicle against the third palpal segment (0.65–0.68 in this new species versus 0.50–0.54 in *S. lurauense*), and male hind basitarsus (0.9 times as wide as the hind tibia and femur in this new species versus 0.8 times as wide as the hind tibia and 0.7–0.8 times as wide as the hind femur in *S. lurauense*).

This new species is similar to *S. quychauense* Takaoka & Chen from Vietnam, which is known only for the male and pupa (Takaoka et al. 2017a) in having a similar number of male upper-eye (large) facets and small pupal terminal hooks. However, it is barely distinguished from the latter species by the ventral margin of the ventral plate nearly straight when viewed posteriorly (somewhat convex ventrally in *S. quychauense*) and pupal abdominal segment 9 with spine-combs (without spine-combs in *S. quychauense*).

This new species is similar to *S. thituyenae* Takaoka & Pham from Vietnam, which is known only for the female and pupa (Takaoka et al. 2015), but is barely distinguished from the latter species by the wing length (1.6 mm in this new species versus 2.0 mm in *S. thituyenae*), number of the outer teeth of the female mandible (four in this new species versus eight in *S. thituyenae*), and the angle of the stalk of the dorsal triplet of the pupal gill against that of the ventral pair of filaments (80–90° in this new species versus 60° in *S. thituyenae*).

#### Simulium (Gomphostilbia) namdanense

Taxon classificationAnimaliaDipteraSimuliidae

Takaoka, Srisuka & Saeung sp. nov.

C147009A-4492-5B23-860A-AAFAC34C7FFA

http://zoobank.org/DA8262B8-729B-4579-B4FC-218F4A476899

Figs 23 , 25F

##### Material examined.

***Holotype***: Male (with its associated pupal exuviae and cocoon) (in 80% ethanol) labeled as “Holotype: *Simulium namdanense* male, QSBG col. no. 59, Thailand, 25-VII-2017, by W. Srisuka”, collected from a medium-sized stream of Nam Khwang (width 4 m, depth 30 cm, bed sandy, fast flow, pH 7.2, 19.5 °C, partially shaded, elevation 1,192 m, 19°11'18.3"N, 101°04'43.7"E), at Nam Dan Village, Pua District, Nan Province, Thailand, 25-VII-2017, by W. Srisuka (Coll. No. 59).

***Paratypes***: Three females, two males (with their associated pupal exuviae and cocoons), and five mature larvae (in 80% ethanol), same data as for holotype; two females, four males (thorax of one male for DNA analysis) (with their associated pupal exuviae and cocoons), and two mature larvae (one mature larva for DNA analysis) (in 80% ethanol), collected from a medium-sized stream (width 3 m, depth 32 cm, bed sandy and rocky, moderate flow, pH 6.5, 23.2 °C, partially shaded, elevation 503 m, 18°48'44.2"N, 98°56'21.3"E), at Huai Kaew Waterfall, Doi Suthep, Muang District, Chiang Mai Province, Thailand, 30-VII-2017, by W. Srisuka (Coll. No. 94).

##### Diagnosis.

**Figure 23.**
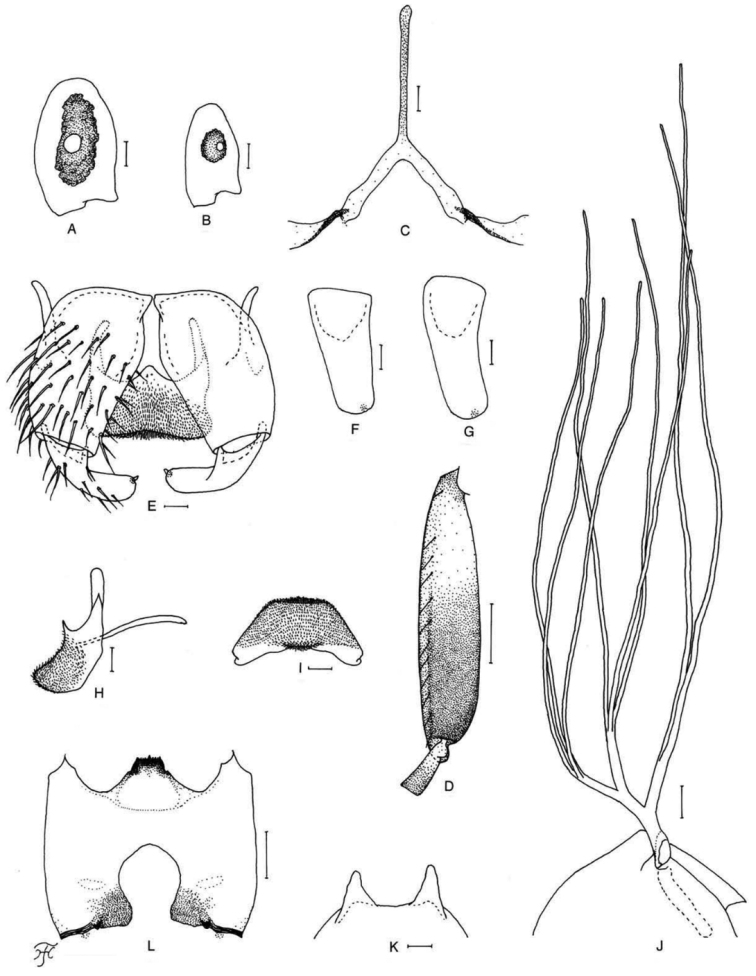
Female, male, pupa and larva of *S. namdanense* sp. nov. **A, C** female **B, D–I** male **J, K** pupa **L** larva. **A, B** sensory vesicles (right side; anterior view **A** female **B** male) **C** genital fork **D** hind basitarsus and second tarsomere (left side; lateral view) **E** coxites, styles and ventral plate (ventral view) **F, G** styles (right side; ventrolateral view) **H** ventral plate and median sclerite (lateral view) **I** ventral plate (caudal view) **J** gill filaments (right side; lateral view) **K** terminal hooks (caudal view) **L** head capsule (ventral view). Scale bars: 0.1 mm (**D, J, L**); 0.02 mm (**A–C, E–I**); 0.01 mm (**K**).

Female: elongate sensory vesicle (Fig. 23A). Male: higher number of upper-eye facets in 15 or 16 vertical columns and 16 or 17 horizontal rows, and hind basitarsus (Fig. 23D) spindle-shaped, narrower than the hind tibia and femur. Pupa: small terminal hooks (Fig. 23K). Larva: postgenal cleft 2.5 times as long as the postgenal bridge (Fig. 23L) and abdominal segments 1–4 each encircled with a light greyish band (Fig. 25F).

##### Description.

**Female** (*N* = 5). Body length 1.8–2.0 mm.

***Head.*** Frons dark brown, densely covered with yellowish white scale-like recumbent short hairs (no dark longer hairs); frontal ratio 1.8–1.9:1.0:2.2–2.4; frons:head ratio 1.0:4.0–4.1. Labrum 0.59–0.63 times length of clypeus. Maxillary palpus: proportional lengths of third, fourth, and fifth palpal segments 1.0:0.9:1.9–2.0; sensory vesicle (Fig. 23A) elongate, (0.64–0.68 times length of third palpal segment), with medium-sized opening. Lacinia with 13 or 14 inner and 16–21 outer teeth. Mandible with 30–32 inner teeth and three to five outer teeth at some distance from tip.

***Legs.*** Foreleg: trochanter light brown; basitarsus moderately dilated, 5.7–5.8 times as long as its greatest width. Midleg: trochanter dark yellow to light brown. Hind leg: coxa light brown; tibia yellowish white on basal half or little more and light brown to brownish black on rest; basitarsus yellowish white except base and little less than apical one-third dark brown, 6.1–6.4 times as long as wide, and 0.7 and 0.5–0.6 times as wide as greatest widths of tibia and femur, respectively; calcipala nearly as long as width at base, and 0.45–0.56 times as wide as greatest width of basitarsus; claw with large basal tooth 0.47–0.52 times length of claw.

***Wing.*** Length 2.0 mm.

***Abdomen.*** Dorsal surface of abdomen medium to dark brown except most of segment 2 (except narrow portion along posterior margin darkened) ochreous.

***Terminalia.*** Sternite 8 with 18–26 medium-long to long hairs together with two or three slender short hairs on each side. Ovipositor valve moderately covered with microsetae interspersed with two to four short hairs. Genital fork (Fig. 23C) with inner margins of arms divergent from each other (not convergent posteriorly). Paraproct with four or five sensilla on anteromedial surface, 0.5 times as long as wide when viewed laterally, and with 20–23 short long hairs on ventral and lateral surfaces. Cercus in lateral view 0.44 times as long as wide. Spermatheca 1.55 times as long as its greatest width; both accessory ducts slender, subequal in diameter to each other and slightly thicker than major one.

**Male** (*N* = 7). Body length 2.0 mm.

***Head.*** Slightly wider than thorax. Upper eye medium brown, consisting of large facets in 15 or 16 vertical columns and 16 or 17 horizontal rows on each side. Antenna: first flagellomere elongate, 1.6–1.8 times length of second. Maxillary palpus light brown, with five palpal segments, proportional lengths of third, fourth, and fifth palpal segments 1.0:1.1–1.3:2.7; third palpomere (Fig. 23B) somewhat enlarged; sensory vesicle (Fig. 23B) medium sized, ellipsoidal (0.33 times length of third palpal segment).

***Legs.*** Foreleg: femur light brown except apical cap medium brown (though apical tip yellowish); tibia light brown except median large portion of outer surface whitish and little less than apical one-third dark brown; basitarsus moderately dilated, 6.5–6.9 times as long as its greatest width. Hind leg: coxa light to medium brown; trochanter whitish yellow; femur light to medium brown with base whitish yellow and apical cap dark brown (though apical tip yellow); tibia dark brown to brownish black except little less than basal half yellow; tarsus (Fig. 23D) dark brown except little less than basal half of basitarsus (though its border not well defined due to covering of dark hairs throughout its length) greyish white and basal half of second tarsomere yellow; basitarsus (Fig. 23D) 4.2–4.4 times as long as wide, and 0.8 and 0.8–0.9 times as wide as greatest width of tibia and femur, respectively; calcipala (Fig. 23D) as long as basal width, and 0.32 times as wide as greatest width of basitarsus.

***Wing.*** Length 1.8–1.9 mm. Subcosta bare or with one to three hairs.

***Genitalia.*** Coxite in ventral view (Fig. 23E) 1.6 times as long as its greatest width. Style in ventral view (Fig. 23E) with round apex; style in ventrolateral view (Fig. 23F, G) slightly tapered or parallel-sided from middle toward apex, with round apex. Ventral plate in ventral view (Fig. 23E) 0.54 times as long as wide; basal arms nearly parallel-sided, then convergent apically; ventral plate in caudal view (Fig. 23I) trapezoidal, though ventral margin slightly concave medially. Parameres each with three distinct long stout hooks and one short hook. Cercus with 13–16 hairs.

**Pupa** (*N* = 12). Body length 2.2–2.5 mm.

***Head.*** Integument yellow. Thorax. Integument yellow, moderately covered with round tubercles except posterior half sparsely covered with minute tubercles on each dorsolateral and lateral surface.

***Thorax.*** Gill (Fig. 23J) composed of eight slender thread-like filaments, arranged as [3+(1+2)]+2 (or rarely [(2+1)+(1+2)]+2 or [(2+1)+3]+2 or (3+3)+2) from dorsal to ventral, with medium-long common basal stalk; common basal stalk 0.8 times length of interspiracular trunk; stalk of ventral pair of filaments 0.7–1.1 times length of common basal stalk, and 0.5–0.8 times length of interspiracular trunk; primary stalk of dorsal triplet lying against that of lower pair at angle of 70–90° when viewed laterally; in one pupal exuviae with intact gill filaments measured, filaments of dorsal triplets subequal in length (1.8 mm) and thickness, those of middle triplet subequal in length (2.0 mm) and thickness, and two filaments of ventral pair subequal in length (2.3–2.5 mm) and thickness to each other; two filaments of ventral pair 1.6–2.0 times as thick as six other filaments of dorsal and middle triplets when compared basally; all filaments light brown.

***Abdomen.*** Dorsally, all segments light yellow, or unpigmented except segments 1, 2 and 9 light yellow; segments 1 and 2 bare; segment 9 with pair of conical terminal hooks (Fig. 23K).

***Cocoon.*** Whitish yellow, slipper-shaped, thinly woven, moderately extended ventrolaterally; anterior margin not thickly woven medially, with no bulge; 2.5–3.0 mm long by 1.5–2.0 mm wide.

**Mature larva** (*N* = 6). Body length 3.9–4.2 mm. Body with following color markings: thoracic segments 1–3 ochreous; abdominal segments 1–4 each encircled with light grey band, abdominal segments 5–8 light grey, overlaid with reddish brown pigment on dorsal and dorsolateral surfaces (though often irregularly faded to varying extent dorsomedially) (Fig. 25F); abdominal segments 5 and 6 each with pair of small round light grey or reddish brown spots ventrally, and abdominal segment 7 with faint light grey or reddish brown transverse band ventrally.

***Head.*** Head capsule yellow, moderately covered with unpigmented minute setae (though sparsely on lateral and ventral surfaces); head spots faintly positive (rarely negative or indistinct). Antenna: proportional lengths of first, second, and third articles 1.0:0.7–0.8:0.8–0.9. Labral fan with 32–36 primary rays. Hypostoma: anterior row of nine teeth, of which median tooth somewhat longer than each lateral tooth, with four or five hypostomal bristles per side lying nearly parallel to lateral margin. Postgenal cleft (Fig. 23L) arrow-head-shaped, medium-long, 2.5 times length of postgenal bridge, usually with apical margin round.

***Thorax*** and ***Abdomen.*** Thoracic and abdominal cuticle almost bare except abdominal segments 5–8 moderately covered with unpigmented and slightly-darkened unbranched minute setae on dorsal and dorsolateral surface; last abdominal segment moderately covered with unpigmented minute setae on each side of anal sclerite to base of ventral papillae. Rectal organ compound, each of three lobes with 8–10 finger-like secondary lobules. Anal sclerite with anterior arms 1.1 times as long as posterior ones. Posterior circlet with 72–84 rows of hooklets with up to 13 or 14 hooklets per row.

##### Etymology.

The species name, *namdanense*, refers to the name of the locality, Nam Dan, where this species was collected.

##### Distribution.

Thailand (Chiang Mai and Nan).

##### Discussion.

This new species is similar to *S. lurauense* described from Peninsular Malaysia (Takaoka et al., 2011b) in many characters including the elongate female sensory vesicle and presence of teeth on the outer margin of the female mandible, male hind basitarsus spindle-shaped, and pupal abdominal segment 9 with a pair of small terminal hooks. However, it is barely distinguished from the latter species in the female by the sensory vesicle 0.64–0.68 times the length of the third maxillary palpal segment (0.50–0.54 times in *S. lurauense*) and in the male by the upper-eye facets in 15 or 16 vertical columns and 16 or 17 horizontal rows (14 or 15 vertical columns and 14 or 15 horizontal rows in *S. lurauense*) and the sensory vesicle 0.33 times the length of the third maxillary palpomere (0.25–0.29 times in *S. lurauense*), and in the pupa by the stalk of the ventral pair of filaments 0.5–0.8 times as long as the interspiracular trunk (0.9–1.0 times in. *S. lurauense*).

This new species is almost indistinguishable from *S. thituyenae* from Vietnam (Takaoka et al. 2015), though there appears to be a slight difference in the number of the outer teeth of the female mandible, which is three to five in this new species and eight in *S. thituyenae*. Further comparison is needed when the male and mature larva of *S. thituyenae* become available.

This new species is distinguished from *S. klonglanense* sp. nov. by the female wing length (2.0 mm in this new species versus 1.6 mm in *S. klonglanense* sp. nov.), and number of male upper-eye facets in 15 or 16 vertical columns and 16 or 17 horizontal rows (13 or 14 vertical columns and 14 or 15 horizontal rows in *S. klonglanense* sp. nov.).

#### Simulium (Gomphostilbia) myanmarense

Taxon classificationAnimaliaDipteraSimuliidae

Takaoka, Srisuka & Saeung, 2017

B5B55319-FED5-550D-8BA3-C75C0B156788

Simulium (Gomphostilbia) myanmarense Takaoka, Srisuka & Saeung, in Takaoka et al. 2017b: 40–45 (female, male, pupa, and larva).

##### Specimens examined.

8 females, 6 males (with their associated pupal exuviae and cocoons), and 20 mature larvae (one mature larva for DNA analysis) (in 80% ethanol), collected from a small stream (width 55 cm, depth 3 cm, bed sandy, moderate flow, pH 7.2, 18.4 °C, partially shaded, elevation 901 m, 19°28'18.3"N, 100°28'00.8"E), at Pha Dang Village, Chiang Kham District, Pha Yao Province, Thailand, 20-III-2018, by W. Srisuka (Coll. No. 75); one female, one male (with their associated pupal exuviae and cocoons), and two mature larvae (in 80% ethanol), collected from a small stream (width 30 cm, depth 5 cm, bed sandy, moderate flow, pH 6.3, 23.6 °C, exposed to the sun, elevation 1,097 m, 18°50'03.7"N, 99°22'32.2"E), at Pa Miang Village, Muang Pan District, Lampang Province, Thailand, 9-VIII-2016, by W. Srisuka (Coll. no. 86).

##### Diagnosis.

Female: mandible lacking outer teeth. Male: upper-eye facets in 15 (rarely 14) vertical columns and 15 (rarely 16) horizontal rows on each side. Pupa: cocoon with a short anterodorsal projection. Larva: medium-sized postgenal cleft and abdominal segments 1 and 2 each encircled with a grey transverse band (Fig. 25L).

##### Distribution.

Myanmar and Thailand (Phayao and Lampang).

##### Discussion.

Jomkumsing et al. (2019) suggested the presence in Thailand of *S. myanmarense* and *S. monglaense*, both originally described from Myanmar (Takaoka et al. 2017b), based on the close similarity of the COI gene sequences among females of ‘*S. asakoae* complex’ caught using a human attractant. Our study confirmed the distribution of *S. myanmarense* in Thailand, based on morphological and molecular evidence.

#### Simulium (Gomphostilbia) inthanonense

Taxon classificationAnimaliaDipteraSimuliidae

Takaoka & Suzuki, 1984

913BE54B-F978-58DE-A7F4-F7640C87B7A7

Figs 24 , 25S, T

Simulium (Gomphostilbia) inthanonense Takaoka & Suzuki, 1984: 18–21 (female, pharate male, pupa, and larva).

##### Remarks.

This species was described from females, pharate males, pupae and larvae collected from Doi Inthanon National park, Chiang Mai Province (Takaoka and Suzuki 1984), and was placed in the *S. ceylonicum* species group (Takaoka 2012).

**Figure 24.**
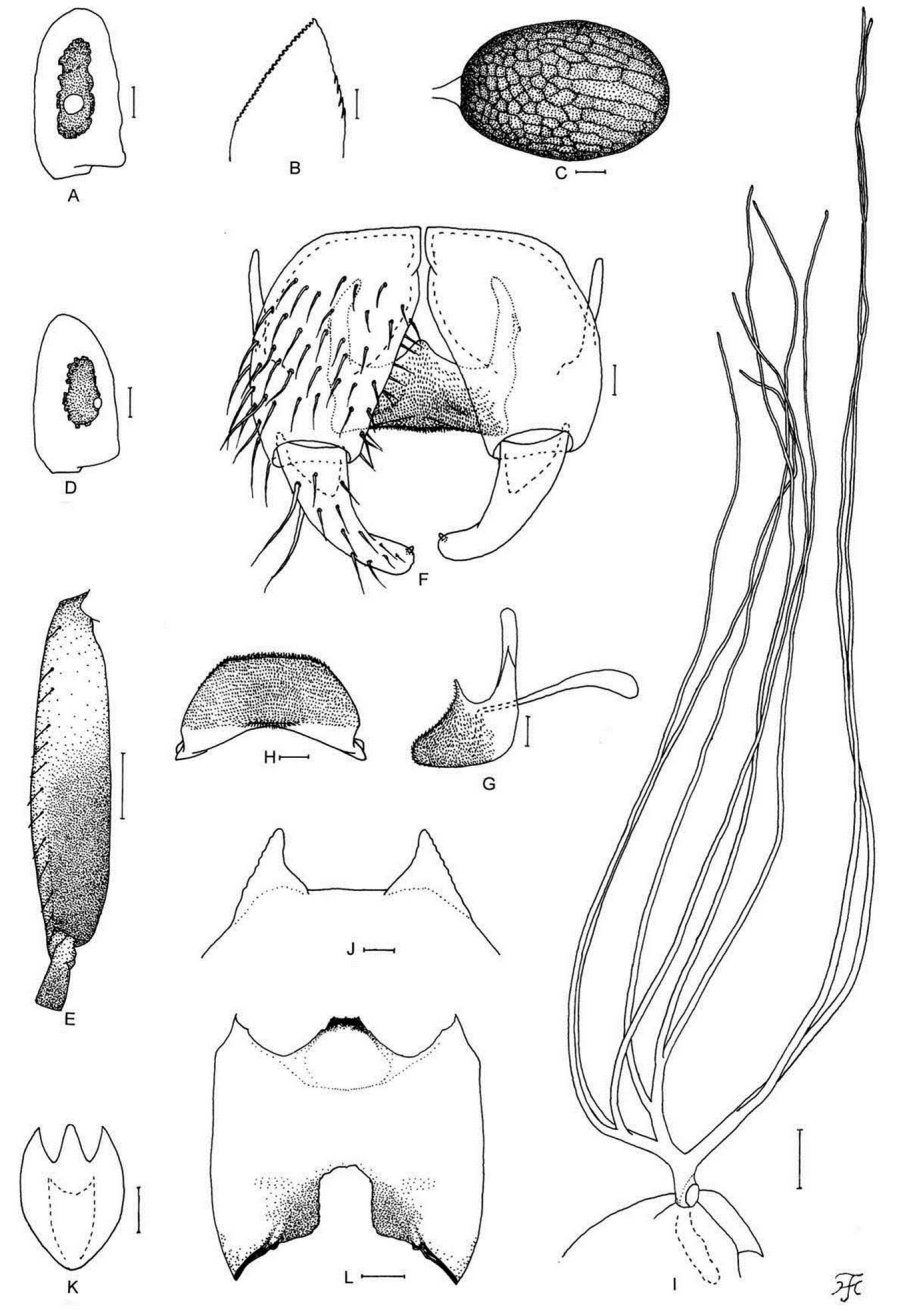
Female, male, pupa and larva of *S. inthanonense*. **A–C** female **D–H** male **I–K** pupa **L** larva. **A, D** sensory vesicles (right side; anterior view **A** female **D** male) **B** mandible (right side) **C** spermatheca **E** hind basitarsus and second tarsomere (left side; lateral view) **F** coxites, styles and ventral plate (ventral view) **G** ventral plate and median sclerite (lateral view) **H** ventral plate (caudal view) **I** gill filaments (right side; lateral view) **J** terminal hooks (caudal view) **K** cocoon (dorsal view) **L** head capsule (ventral view). Scale bars: 1.0 mm (**K**); 0.1 mm (**E, I, L**); 0.02 mm (**A, C, D, F–H**); 0.01 mm (**B, J**).

This species is here transferred from the *S. ceylonicum* species group to the *S. asakoae* species group, based on the male ventral plate emarginated on both sides when viewed ventrally (Fig. 24F), though the dark tuft hairs on the base of the radial vein of the female and the male depart from the definition of the species group (Takaoka 2012). An analysis of the COI gene sequences supports this transfer (Fig. 26). The descriptions of the female, pupa and larva are revised, and the male is fully described for the first time.

**Figure 25.**
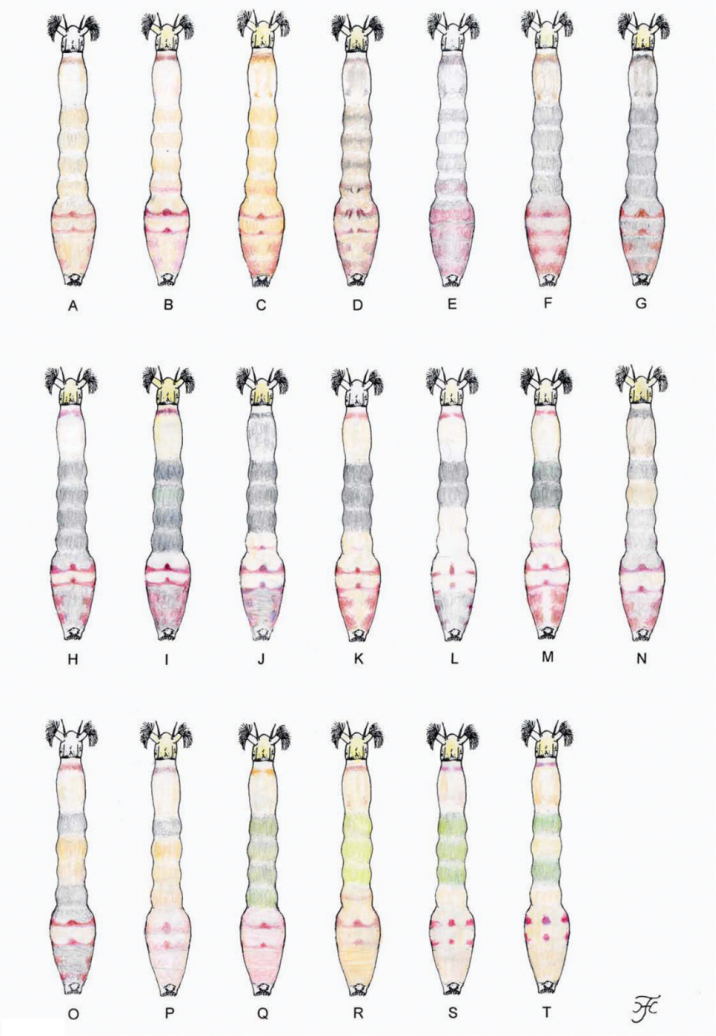
Schematic illustrations of larval body color patterns (dorsal view). **A***S. teerachanense* sp. nov. **B***S. pitasawatae* sp. nov. **C***S. sutheppuiense* sp. nov. **D***S. asakoae***E***S. klonglanense* sp. nov. **F***S. namdanense* sp. nov. **G***S. chaowaense* sp. nov. **H***S. loeiense* sp. nov. **I***S. huaimorense* sp. nov. **J***S. nanoiense* sp. nov. **K***S. junkumae* sp. nov. **L***S. myanmarense***M***S. maewongense* sp. nov. **N***S. phapeungense* sp. nov. **O***S. muangpanense* sp. nov. **P***S. maehongsonense* sp. nov. **Q***S. thungchangense* sp. nov. **R***S. chiangdaoense***S***S. inthanonense* (morph 1) **T***S. inthanonense* (morph 2).

**Figure 26.**
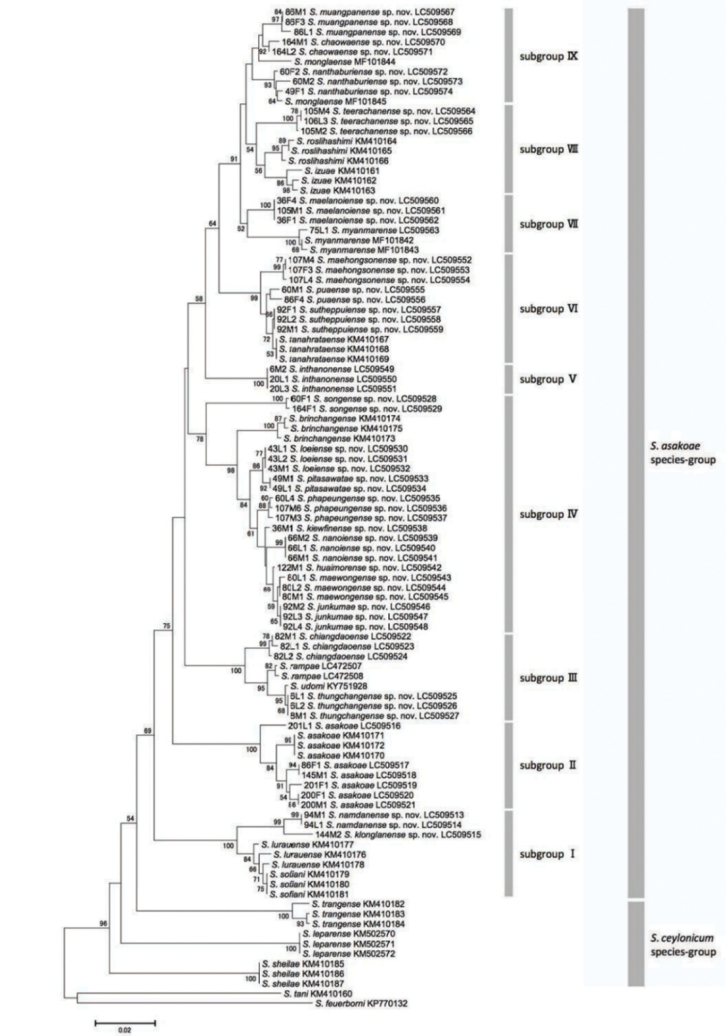
Neighbor-joining tree of the 26 Thai species of the *Simulium asakoae* species group based on COI gene sequences (586 positions). The numbers at the nodes mean the bootstrap confidence values after 500 replicates. The bootstrap values above 50% are shown. The scale bar indicates the distance in substitutions per nucleotide. The symbols in front of the species names are each the collection number (corresponding to those in the text), developmental stages (F, female; M, male; L, larva), and sample number (if two or more samples were examined) in this order.

##### Specimens examined.

Ten females, 10 males (with their associated pupal exuviae and cocoons) (in 80% ethanol), collected from a small stream (width 40 cm, depth 10 cm, bed sandy, moderate flow, pH 7.2, 15.3 °C, partially shaded, elevation 1,314 m, 18°31'01.9"N, 98°28'17.3"E), at Mae Klang Watershed, Doi Inthanon National Park, Mae Cham District, Chiang Mai Province, Thailand, 21-XII-2018, by W. Srisuka (Coll. No. 25); five females and five males and five mature larvae (two mature larvae for DNA analysis) (in 80% ethanol) collected in a stream (width 1.0 m, depth 20 cm, bed sandy, flow moderate, partially shaded, pH 6.8, 14.0 °C, elevation 1,589 m, 18°30'29.8"N, 98°30'37.4"E), at Mae Aum, Doi Inthanon National Park, Chom Thong District, Chiang Mai Province, Thailand, 25-I-2019, by W. Srisuka (Coll. No. 20). One male (thorax for DNA analysis) (with its associated pupal exuviae and cocoon) (in 80% ethanol), collected from a stream (width 70 cm, depth 10 cm, pH 6.5, 13.8 °C, moderate flow, bed sandy, partially shaded, elevation 1,685 m, 18°31'15.4"N, 98°29'59.4"E), before check point 2, Doi Inthanon National parks, Chiang Mai Province, northern Thailand, 20-III-2018, by W. Srisuka (Coll. No. 6).

##### Diagnosis.

Female and male: darkened hair tuft at the base of the radial vein and relatively long sensory vesicle (Fig. 24A, D). Female: mandible with several distinct teeth on the outer margin (Fig. 24B), and hind tibia yellowish on the basal half. Male: greater number of large upper-eye facets in 17 vertical columns and 17 (rarely 18) horizontal rows on each side, subcosta without hairs, and hind basitarsus (Fig. 24E) narrower than the hind tibia and femur. Pupa: dorsal surface of abdominal segments 1–5 light greyish brown. Larva: postgenal cleft (Fig. 24L) as long as or little shorter than the postgenal bridge and abdominal segments 1 and 3 (or 1–3) greenish grey (Fig. 25S, T). Two forms of this species are designated, based on larvae: morph 1 has abdominal segments 1–3 greenish grey (Fig. 25S) and morph 2 has abdominal segments 1 and 3 greenish grey (Fig. 25T).

##### Description.

**Female** (*N* = 15). Body length 2.0–2.1 mm.

***Head.*** Frontal ratio 1.6–1.8:1.0:2.3–2.8; frons:head ratio 1.0:4.7–5.1. Labrum 0.55–0.61 times length of clypeus. Maxillary palpus: proportional lengths of third, fourth, and fifth palpal segments 1.0:0.9–1.0:2.1–2.2; sensory vesicle (Fig. 24A) elongate, 0.58–0.61 times length of third palpal segment. Lacinia with 10–13 inner and 15–17 outer teeth. Mandible (Fig. 24B) with 28–30 inner teeth and four to six outer teeth at some distance from tip.

***Legs.*** Foreleg: basitarsus moderately dilated, 6.2–6.4 times as long as its greatest width. Hind leg: coxa medium brown; basitarsus 7.1–7.2 times as long as wide, and 0.6–0.7 and 0.5–0.6 times as wide as greatest widths of tibia and femur, respectively; calcipala nearly as long as width at base, and 0.6 times as wide as greatest width of basitarsus; claw with large basal tooth 0.43 times length of claw.

***Wing.*** Length 2.1–2.4 mm. Costa with dark spinules and hairs. Hair tuft on base of radius dark brown.

***Abdomen.*** Dorsal surface of abdomen medium to dark brown except most of segment 2 whitish yellow.

***Terminalia.*** Sternite 8 with 16–25 medium-long to long hairs together with three or four slender short hairs on each side. Ovipositor valves each covered with microsetae interspersed with three or four short hairs. Paraproct with five to eight sensilla on anteromedial surface; paraproct in lateral view with 26–32 medium-long to long hairs on ventral and lateral surfaces. Cercus in lateral view short, slightly rounded posteriorly, 0.5 times as long as wide. Spermatheca (Fig. 24C) ellipsoidal, 1.4–1.5 times as long as its greatest width, and with many fissures nearly entirely on outer surface in some females.

**Male** (*N* = 16). Body length 2.3–2.6 mm.

***Head.*** Slightly wider than thorax. Upper eye medium brown, consisting of large facets in 17 vertical columns and 17 (rarely 18) horizontal rows on each side. Antenna medium to dark brown except scape, pedicel and base of first flagellomere yellow, though apical half or two-thirds of pedicel light brown in some males; first flagellomere 1.7 times length of second. Maxillary palpus: proportional lengths of third, fourth, and fifth palpal segments 1.0:1.2:2.6; third palpomere (Fig. 24D) somewhat enlarged; sensory vesicle (Fig. 24D) medium-long, 0.40–0.47 times length of third palpal segment.

***Legs.*** Foreleg: coxa yellow; trochanter light brown; femur medium brown; light brown except apical one-third dark brown and median large portion on outer surface white and shiny; basitarsus moderately dilated, 6.9–7.7 times as long as its greatest width. Midleg: trochanter light brown except base yellow; femur medium brown with base yellowish and apical cap dark brown (though apical tip yellow); tarsus light to dark brown. Hind leg: coxa medium brown; trochanter yellow to dark yellow; tarsus (Fig. 24E) brownish black except basal two-fifths of basitarsus and basal one-third of second tarsomere dark yellow to light brown; basitarsus (Fig. 24E) 4.2–4.4 times as long as wide, and 0.8–0.9 and 0.8–0.9 times as wide as greatest width of tibia and femur, respectively; calcipala (Fig. 24E) slightly shorter than basal width, and 0.35 times as wide as greatest width of basitarsus.

***Wing.*** Length 2.1–2.3 mm. Other characters as in female except subcosta bare. *Halter.* Light to medium brown except lower portion whitish.

***Genitalia.*** Coxite in ventral view (Fig. 24F) nearly rectangular, 1.7–1.8 times as long as its greatest width. Style in ventrolateral view slightly tapered toward apex, with round apex. Ventral plate in ventral view (Fig. 24F) with basal arms nearly parallel-sided, then convergent apically; ventral plate in caudal view (Fig. 24H) with ventral margin nearly straight. Cercus with 12–14 hairs.

**Pupa** (*N* = 31). Body length 2.5–3.0 mm.

***Head.*** Integument yellow.

***Thorax.*** Integument yellow, with two somewhat darkened areas in tandem on dorsal surface of posterior half. Gill (Fig. 24I) composed of eight slender thread-like filaments, arranged as [(2+1)+(1+2)]+2 from dorsal to ventral; common basal stalk 0.6–0.7 times length of interspiracular trunk; dorsal and middle triplets sharing short stalk, and composed of one individual and two paired filaments; stalk of ventral pair of filaments 1.3–1.7 times length of common basal stalk, and 0.9–1.1 times length of interspiracular trunk; primary stalk of dorsal triplet lying against that of ventral pair at angle of 80–120° when viewed laterally; filaments of dorsal triplet subequal in length (2.2–3.2 mm) and thickness to one another; those of middle triplets subequal in length (2.8–3.8) and thickness to one another, two filaments of ventral pair subequal in length (3.5–4.2 mm) and thickness to each other and 1.1–1.5 times as thick as six other filaments of dorsal and middle triplets when compared basally.

***Abdomen.*** Dorsally, segments 1–5 light greyish brown, segment 9 and bases of spine-combs of segments 6–8 light yellow; segments 1 and 2 without minute tubercles; segment 9 with pair of triangular terminal hooks (Fig. 24J), of which outer margin 1.3–1.7 times length of the inner margin and weakly crenulated when viewed caudally.

***Cocoon*** (Fig. 24K). Yellow to dark brown, slipper-shaped, moderately woven, widely extended ventrolaterally; anterior margin thickly woven medially and with short projection (its length usually 1.0 mm or slightly more); 3.2–3.9 mm long by 1.6–3.0 mm wide.

**Mature larva** (*N* = 3). Body length 5.6–6.0 mm. Body light ochreous with following color markings: thoracic segment 1 encircled with distinct reddish brown band (though disconnected ventromedially), thoracic segments 2 and 3 dark ochreous on ventral surface; abdominal segments 1 and 3 (or abdominal segments 1–3) greenish grey; abdominal segments 5 and 6 each with three distinct, reddish brown spots (one round dorsomedial spot and two dorsolateral spots) (Fig. 25S, T).

***Head.*** Head spots faintly to moderately positive. Antenna: proportional lengths of first, second, and third articles 1.0:1.0–1.1:0.8–0.9. Labral fan with 40 primary rays. Hypostoma with four to six hypostomal bristles per side lying nearly parallel to or slightly diverginf from lateral margin. Postgenal cleft (Fig. 24L) short, nearly quadrate, as long as or little shorter than postgenal bridge.

***Thorax*** and ***Abdomen.*** Thoracic and abdominal cuticle sparsely covered with unpigmented minute setae. Rectal organ compound, each of three lobes with 9–13 finger-like secondary lobules. Anal sclerite with anterior arms nearly as long as posterior ones. Posterior circlet with 82–87 rows of hooklets with up to 15 hooklets per row.

##### Distribution.

Thailand (Chiang Mai), China and Vietnam.

#### Simulium (Gomphostilbia) chiangdaoense

Taxon classificationAnimaliaDipteraSimuliidae

Takaoka & Srisuka, 2009

E3EE2109-6101-5EC8-B5EF-51F04E639EEF

Simulium (Gomphostilbia) chiangdaoense Takaoka and Srisuka 2009: 269–276 (female, male, and pupa) (Takaoka and Srisuka 2009).

##### Specimens used for DNA analysis.

One male and two mature larvae, collected from a small stream (width 55 cm, depth 3 cm, bed sandy, moderate flow, pH 7.2, 18.4 °C, partially shaded, elevation 901 m, 19°28'18.3"N, 100°28'00.8"E), at Pha Tea Do mountain, Mae Cham, Chiang Mai Province, northern Thailand, 27-VI-2018, by W. Srisuka (Coll. No. 82).

#### Simulium (Gomphostilbia) asakoae

Taxon classificationAnimaliaDipteraSimuliidae

Takaoka & Davies, 1995

FB275C26-8282-5CF7-8D81-79BE020D87D1

Simulium (Gomphostilbia) asakoae Takaoka and Davies 1995: 55–60 (female, male pupa, and larva).

##### Specimens used for DNA analysis.

One female, reared from a pupa collected from a stream (width 30 cm, depth 5 cm, bed sandy, moderate flow, pH 6.3, 23.6 °C, exposed to the sun, elevation 1,097 m, 18°50'03.7"N, 99°22'32.2"E), at Pa Miang Village, Chae Hom, Lampang Province, Thailand, 9-VIII-2016, by W. Srisuka (Coll. No. 86); one male, reared from a pupa collected from a stream (width 5 m, depth 25 cm, pH 6.9, 22.1 °C, moderate flow, bed sandy, partially shade, elevation 759 m, 16°19'01.2"N, 99°06'19.9"E) near Tao Dam Waterfall, Klong Lan, Kam Phaeng Phet Province, Thailand, 20-XII-2016, by W. Srisuka (Coll. No. 145); one female and one male, reared from pupae collected from a stream (width 2.5 m, depth 30 cm, moderate flow, bed sandy, partially shaded, pH 5.9, 19 °C, elevation 1,293 m, 16°58'49.5"N, 101°03'29.3"E) at Rangkla Village, Nakhon Thai District, Phitsanulok Province, Thailand, 18-VI-2019, by W. Srisuka (Coll. No. 200); one female reared from a pupa and one mature larva collected from a stream (width 50 cm, depth 16 cm, moderate flow, bed sandy, pH 7.3, 21 °C, elevation 1,231 m, 18°32'16.3"N, 98°31'30.5"E) at Khun Klang Village, Doi Inthanon National Park, Chiang Mai Province, northern Thailand, 24-VII-2019, by W. Srisuka (Coll. No. 201).

### Keys to identify the 27 Thai species of the *S. asakoae* species group

**Female** ^*^

**Table:** 

1	Hair tuft of base of radial vein brownish	***S. inthanonense***
–	Hair tuft of base of radial vein yellow	**2**
2	Sensory vesicle elongate, more than 0.5 times length of third palpal segment (Fig. 22A)	**3**
–	Sensory vesicle short to medium-long, less than 0.4 times length of third palpal segment (Fig. 1A)	**4**
3	Wing length 1.6 mm	***S. klonglanense* sp. nov.**
–	Wing length 2.0 mm	***S. namdanense* sp. nov.**
4	Mandible with distinct teeth on outer margin (Fig. 22B)	**5**
–	Mandible without distinct teeth on outer margin (Fig. 14A)	**16**
5	Body length 1.9–2.2 mm	**6**
–	Body length 2.4–2.6 mm	**14**
6	Claw tooth 0.50–0.53 times length of claw (Fig. 1E)	**7**
–	Claw tooth 0.43–0.47 times length of claw	**11**
7	Claw tooth 0.53 times length of claw	***S. udomi***
–	Claw tooth 0.50 times length of claw	**8**
8	Sensory vesicle 0.22–0.24 times length of third palpal segment (Fig. 12A)	***S. nanoiense* sp. nov.**
–	Sensory vesicle 0.26–0.32 times length of third palpal segment	**9**
9	Labrum 0.6 times as long as clypeus	***S. sutheppuiense* sp. nov.**
–	Labrum 0.7 times as long as clypeus **10**
10	Fifth palpal segment 2.6 times as long as third	***S. loeiense* sp. nov.**
–	Fifth palpal segment 2.1 times as long as third	***S. maelanoiense* sp. nov.**
11	Sensory vesicle 0.24 times length of third palpal segment	***S. puaense* sp. nov.**
–	Sensory vesicle 0.26–0.35 times as long as third palpal segment	**12**
12	Sensory vesicle 0.32–0.35 times as long as third palpal segment	***S. pitasawatae* sp. nov.**
–	Sensory vesicle 0.26–0.29 times length of third palpal segment	**13**
13	Spermatheca 1.3 times as long as wide	***S. huaimorense* sp. nov.**
–	Spermatheca 1.6 times as long as wide	***S. nanthaburiense* sp. nov.**
14	Labrum 0.54–0.56 times length of clypeus	***S. asakoae***
–	Labrum 0.61–0.68 times length of clypeus	**15**
15	Claw tooth 0.50 times length of claw (Fig. 1E)	***S. thungchangense* sp. nov.**
–	Claw tooth 0.47 times length of claw	***S. chiangdaoense***
16	Sensory vesicle 0.36 times as long as third palpal segment; claw tooth 0.56 times as long as claw	***S. maewongense* sp. nov.**
–	Sensory vesicle 0.25–0.32 times as long as third palpal segment; claw tooth 0.46–0.50 times as long as claw	**17**
17	Fore basitarsus 5.5 times as long as its greatest width	**18**
–	Fore basitarsus 5.9–6.5 times as long as its greatest width	**19**
18	Labrum 0.52 times as long as clypeus	***S. maehongsonense* sp. nov.**
–	Labrum 0.65 times as long as clypeus	***S. songense* sp. nov.**
19	Hind basitarsus 6.5 times as long as its greatest width	***S. myanmarense***
–	Hind basitarsus 5.9–6.2 times as long as its greatest width	**20**
20	Frons: head ratio 1.0:4.7–5.5	***S. junkumae* sp. nov.**
–	Frons: head ratio 1.0:4.0	***S. muangpanense* sp. nov.**

**Males** ^*^

**Table:** 

1	Hair tuft of base of radial vein brownish	***S. inthanonense***
–	Hair tuft of base of radial vein yellow	**2**
2	Upper-eye (large) facets vermilion	**3**
–	Upper-eye (large) facets medium to dark brown	**4**
3	Upper-eye (large) facets in 9 or 10 vertical columns and 12 horizontal rows; fore basitarsus 8.4–8.7 times as long as its greatest width	***S. thungchangense* sp. nov.**
–	Upper-eye (large) facets in 11 or 12 vertical columns and 13 or 14 horizontal rows; fore basitarsus 7.1–7.4 times as long as its greatest width	***S. asakoae***
4	Antenna yellowish except few apical flagellomeres slightly to somewhat greyish	***S. teerachanense* sp. nov.**
–	Antenna brownish except scape, pedicel and base of first flagellomere yellowish	**5**
5	Hind basitarsus 0.9 time as wide as hind femur (Fig. 7A)	**6**
–	Hind basitarsus 1.0–1.3 times as wide as hind femur (Fig. 4C)	**11**
6	Ventral plate with ventral margin rounded when viewed caudally (Fig. 17D)	**7**
–	Ventral plate with ventral margin nearly straight medially when viewed caudally (Fig. 20F)	**9**
7	Fore basitarsus 6.4 times as long as its greatest width	***S. banluangense* sp. nov.**
–	Fore basitarsus 7.4–8.8 times as long as its greatest width	**8**
8	Wing length 1.6–1.7 mm	***S. chaowaense* sp. nov.**
–	Wing length 2.0 mm	***S. myanmarense***
9	Upper-eye (large) facets in 13 vertical columns	***S. muangpanense* sp. nov.**
–	Upper-eye(large) facets in 14–16 vertical columns	**10**
10	Upper-eye (large) facets in 14 vertical columns	***S. klonglanense* sp. nov.**
–	Upper-eye (large) facets in 15 or 16 vertical columns	***S. namdangense* sp. nov.**
11	Upper-eye (large) facets in 9 or 10 vertical columns	***S. puaense* sp. nov.**
–	Upper-eye (large) facets in 11 or more vertical columns	**12**
12.	Upper-eye (large) facets in 11 vertical columns	**13**
–	Upper-eye (large) facets in 12–17 vertical columns	**16**
13	Head nearly as wide as thorax	**14**
–	Head slightly wider than thorax	**15**
14	Ventral plate rounded ventrally when viewed caudally (Fig. 7D)	***S. maewongense* sp. nov.**
–	Ventral plate with ventral margin nearly straight when viewed caudally (Fig. 8E)	***S. loeiense* sp. nov.**
15	First antennal flagellomere 1.8–1.9 times as long as second	***S. sutheppuiense* sp. nov. and *S. maelanoiense* sp. nov.**
–	First antennal flagellomere 1.5–1.6 times as long as second	***S. phapeungense* sp. nov.**
16	Upper-eye (large) facets in 16 or 17 vertical columns	**17**
–	Upper-eye (large) facets in 12–14 vertical columns	**19**
17	Subcosta bare or with two hairs; wing length 1.9–2.0 mm	***S. huaimorense* sp. nov. and *S. kiewfinense* sp. nov.**
–	Subcosta with 7–12 hairs; wing length 2.2–2.5 mm	**18**
18	Ventral plate with ventral margin nearly straight when viewed caudally (Fig. 18I)	***S. junkumae* sp. nov.**
–	Ventral plate with ventral margin rounded when viewed caudally	***S. rampae***
19	Upper-eye (large) facets in 13 or 14 horizontal rows	**20**
–	Upper-eye (large) facets in 15 or 16 horizontal rows	**22**
20	Ventral plate with ventral margin rounded when viewed caudally (Fig. 12G)	***S. nanoiense* sp. nov.**
–	Ventral plate with ventral margin nearly straight when viewed caudally (Fig. 11F)	**21**
21	Upper-eye (large) facets in 12 vertical columns	***S. nanthaburiense* sp. nov.**
–	Upper-eye (large) facets in 13 vertical columns	***S. maehongsonense* sp. nov.**
22	Ventral plate with ventral margin nearly straight when viewed caudally (Fig. 16F)	***S. pitasawatae* sp. nov.**
–	Ventral plate with ventral margin rounded when viewed caudally	***S. chiangdaoense* and *S. udomi***

**Pupae**

**Table:** 

1	Cocoon with elongate anterodorsal projection (2.0 mm or little longer) extended far beyond anteroventral tips of cocoon	**2**
–	Cocoon with or without anterodorsal bulge or short anterodorsal projection (up to ca. 1.0 mm long) extended at most up to anteroventral tips of cocoon	**3**
2	Gill with six filaments	***S. udomi***
–	Gill with eight filaments	***S. chiangdaoense***
3	Dorsum of abdominal segments 1 and 2 or 1–3 or 1–5 light to medium brown or light greyish brown	**4**
–	Dorsum of abdominal segments 1 and 2 unpigmented or light yellowish	**5**
4	Dorsum of abdominal segments 1 and 2 or 1–3 light to medium brown; cocoon with short bulge	***S. asakoae***
–	Dorsum of abdominal segments 1–5 light greyish brown; cocoon with short projection (Fig. 24K)	***S. inthanonense***
5	Dorsum of abdominal segment 1 sparsely covered with minute tubercles	**6**
–	Dorsum of abdominal segment 1 without minute tubercles	**7**
6	Outer margin of terminal hook 2.2 times length of inner margin (Fig. 8G)	***S. loeiense* sp. nov.**
–	Outer margin of terminal hook 2.7 times length of inner margin (Fig. 3B)	***S. thungchangense* sp. nov.**
7	Cocoon with short anterodorsal projection (Figs 7G, 20I)	**8**
–	Cocoon without anterodorsal projection	**9**
8	Primary stalks of dorsal and middle triplets of gill filaments much shorter than their common stalk (Fig. 20G)	***S. huaimorense* sp. nov.**
–	Primary stalks of dorsal and middle triplets of gill filaments nearly as long as their common stalk (Fig. 7E)	***S. maewongense* sp. nov. and *S. myanmarense***
9	Terminal hooks conical, with outer margin as long as or slightly longer than inner margin and not crenulated (Figs 22K, 23K)	***S. klonglanense* sp. nov. and *S. namdanense* sp. nov.**
–	Terminal hooks widened, plate-like, with outer margin over 1.5 times length of inner margin, and crenulated	**10**
10	Stalk of ventral pair thicker than interspiracular trunk (Fig. 17E, F)	***S. banluangense* sp. nov.**
–	Stalk of ventral pair thinner than interspiracular trunk	**11**
11	Gill arranged as 3+2+(2+1) or 2+2+(2+1) from dorsal to ventral, with long common basal stalk 1.3–1.5 times length of interspiracular trunk	***S. rampae***
–	Gill arranged as (3+3)+2 from dorsal to ventral, with medium-long common basal stalk shorter than interspiracular rrunk	**12**
12	Outer margin of terminal hook 2.0–2.9 times as long as inner margin	***S. chaowaense* , sp. nov., *S. junkumae* sp. nov., *S. maelanoiense* sp. nov., *S. muangpanense* sp. nov., *S. nanoiesne* sp. nov., *S. phapeungense* sp. nov., *S. puaense* sp. nov., *S. songense* sp. nov., *S. sutheppuiense* sp. nov. and *S. teerachanense* sp. nov.**
–	Outer margin of terminal hook 3.3–4.0 times as long as inner margin	**13**
13	Filaments of ventral pair 2.1–2.3 mm long (Fig. 14G)	***S. maehongsonense* sp. nov.**
–	Filaments of ventral pair 2.5–3.0 mm long (Fig. 16G)	***S. pitasawatae* sp. nov.**

**Larvae** ^*^

**Table:** 

1	Abdominal segments 1–4 unicolorous	**2**
–	Abdominal segments 1–4 not unicolorous	**12**
2	Abdominal segments 1–4 light ochreous (Fig. 25A, B, C)	**3**
–	Abdominal segments 1–4 greyish or greenish grey or greenish	**5**
3	Postgenal cleft 2.8–3.3 times as long as postgenal bridge (Fig. 6I)	***S. teerachanense* sp. nov.**
–	Postgenal cleft 1.2–2.4 times as long as postgenal bridge (Fig. 16K)	**4**
4	Labral fan with 23 or 24 primary rays	***S. pitasawatae* sp. nov.**
–	Labral fan with 29 or 30 primary rays	***S. sutheppuiense* sp. nov.**
5	Pharate pupal gill with elongate common basal stalk	***S. rampae***
–	Pharate pupal gill with medium-long common basal stalk	**6**
6	Postgenal cleft 1.5–4.0 times length of postgenal bridge	**7**
–	Postgenal cleft 0.8–1.4 times length of postgenal bridge	**9**
7	Head spots moderately positive	***S. asakoae***
–	Head spots indistinct or faintly positive	**8**
8	Postgenal cleft 2.5 times as long as postgenal bridge (Fig. 23L)	***S. namdanense* sp. nov.**
–	Postgenal cleft 3.7–4.0 times as long as postgenal bridge (Fig. 22M)	***S. klonglanense* sp. nov.**
9	Abdominal segments 5–8 pinkish (Fig. 25Q)	***S. thungchangense* sp. nov.**
–	Abdominal color pattern otherwise	**10**
10	Abdominal segments 7 and 8 light grey but abdominal segments 5 and 6 unpigmented (Fig. 25I)	***S. huaimorense* sp. nov.**
–	Abdominal color pattern otherwise	**11**
11	Abdominal segments 5, 7 and 8 grey but abdominal segment 6 unpigmented (Fig. 25H)	***S. loeiense* sp. nov.**
–	Abdominal segments 5, 6, 7 and 8 greyish (Fig. 25G)	***S. chaowaense* sp. nov.**
12	Abdominal segment 1 grey but abdominal segments 2–4 ochreous (Fig. 25P)	***S. maehongsonense* sp. nov.**
–	Abdominal color pattern otherwise	**13**
13	Abdominal segments 1 and 2 grey or green but abdominal segments 3 and 4 unpigmented	**14**
–	Abdominal color pattern otherwise	**15**
14	Abdominal segments 7 and 8 grey with faint reddish brown pigment (Fig. 25L)	***S. myanmarense***
–	Abdominal segments 7 and 8 not grey with distinct reddish brown pigment (Fig. 25M)	***S. maewongense* sp. nov.**
15	Abdominal segments 1, 2 and 3 grey or greenish grey but abdominal segment 4 unpigmented or light ochreous (Fig. 25J, K, S)	**16**
–	Abdominal color pattern otherwise	**18**
16	Abdominal segments 7 and 8 grey with reddish brown pigment (Fig. 25J)	***S. nanoiense* sp. nov.**
–	Abdominal segments 7 and 8 light ochreous (Fig. 25K, S)	**17**
17	Abdominal segments 1, 2 and 3 greyish, and segments 7 and 8 with reddish brown pigment (Fig. 25K)	***S. junkumae* sp. nov.**
–	Abdominal segments 1, 2 and 3 greenish grey but segments 7 and 8 lacking reddish brown pigment (Fig. 25S)	***S. inthanonense* (morph 1)**
18	Abdominal segments 1 and 3 greenish grey (Fig. 25T)	***S. inthanonense* (morph 2)**
–	Abdominal color pattern otherwise	**19**
19	Abdominal segments 1, 3 and 4 grey but abdominal segment 2 light ochreous (Fig. 25N)	***S. phapeungense* sp. nov.**
–	Abdominal color pattern otherwise	**20**
20	Abdominal segments 1 and 4 greyish but abdominal segments 2 and 3 light ochreous (Fig. 25O)	***S. muangpanense* sp. nov.**
–	Abdominal segments 1–3 light green but abdominal segment 4 light ochreous (Fig. 25R)	**21**
21	Pharate pupal gill with six filament	***S. udomi***
–	Pharate pupal gill with eight filaments	***S. chiangdaoense***

## Genetic analysis

The genetic relationships of all 26 species (exclusive of *S. banluangense* sp. nov.) of the *S. asakoae* species group in Thailand are shown in Fig. 26. All 26 species are divided into nine subgroups, I–IX, each consisting of two, one, four, nine, one, three, two, one, and three species. Genetic distances between the nine subgroups are shown in Table 1.

**Table 1.** Nucleotide differences (%) among subgroups of the *Simulium asakoae* species group based on COI sequences.

	I	II	III	IV	V	VI	VII	VIII	IX
I	0–3.41								
II	7.68–9.04	0–2.22							
III	7.34–9.39	5.97–7.68	0–2.39						
IV	6.66–9.22	6.31–7.85	5.46–7.17	0–5.29					
V	6.66–7.85	5.63–6.31	5.12–5.97	5.12–6.14	0				
VI	6.31–8.87	6.48–7.34	5.80–7.85	4.95–6.66	3.92–4.78	0–4.61			
VII	7.51–9.73	7.34–8.36	5.46–7.00	5.46–7.51	4.27–4.78	3.41–5.12	0–2.90		
VIII	7.51–9.73	6.48–8.87	5.80–6.83	5.29–6.83	4.27–5.12	4.10–5.12	2.90–4.27	0–3.41	
IX	7.17–9.22	6.83–8.02	5.46–7.00	5.12–6.66	4.27–5.12	3.24–4.61	2.05–3.92	1.88–3.58	0–2.56

In subgroup I, two new species, *S. klonglanense* and *S. namdanense*, and two known species, *S. lurauense* and *S. sofiani* (Takaoka et al. 2011a, b), from Peninsular Malaysia are included. *Simulium klonglanense* sp. nov. and *S. namdanense* sp. nov. are close in their positions to each other but are distinguished by a nucleotide difference of 1.54 %. The nucleotide differences between these new species and *S. lurauense* were 2.90–3.41 % and 2.73–3.24 %, respectively.

Subgroup I is an assemblage of species that is morphologically characterized by the combination of an elongate female sensory vesicle and small pupal terminal hooks.

Subgroup II is represented only by *S. asakoae*. A similar distinct genetic status of this species within the species group was also shown by Jomkumsing et al. (2019).

None of six samples from four localities of Thailand was identical to those of *S. asakoae* from Peninsular Malaysia. The differences between six samples from Thailand and *S. asakoae* from Peninsular Malaysia were 1.22–1.98 % and 1.37–2.22 %, based on the sequences of 685 bp and 586 bp, respectively. These differences are regarded as intraspecific variation because four Thai populations examined in this study are morphologically indistinguishable from *S. asakoae* from Peninsular Malaysia. Relatively high intraspecific variation is likely to reflect a wide range of its aquatic habitats from low to high elevations (196 m to 1,610 m) in Thailand.

In subgroup III, one new species, *S. thungchangense*, and three known species, *S. chiangdaoense* (of which the COI gene sequences were newly added in this study), *S. rampae*, and *S. udomi* (Srisuka et al. 2019, Takaoka et al. 2009, Takaoka et al. 2006), are included. *Simulium thungchangense* sp. nov. and *S. udomi* have nearly identical COI sequences, although both species are morphologically different from each other by the number of pupal gill filaments (eight versus six).

In subgroup IV, nine new species and one known species, *S. brinchangense* from Peninsular Malaysia, are included. A high similarity of COI gene sequences of four new species, *S. huaimorense*, *S. junkumae*, *S. kiewfinense*, and *S. maewongense*, is observed. Notably, the first three of these new species are morphologically similar in sharing a higher number of male upper-eye large facets, suggesting that they have recently been derived from a common ancestor.

Subgroup V is represented only by *S. inthanonense*, which was formerly treated as a member of the *S. ceylonicum* species group (Takaoka 2012). The result that its genetic relationship is closer to members of the *S. asakoae* species group than to those of the *S. ceylonicum* species group supports the transfer of this species to the *S. asakoae* species group based on morphological characters.

In subgroup VI, *S. sutheppuiense* sp. nov. forms a close relationship with *S. tanahrataense* from Peninsular Malaysia, although both species differ morphologically from each other.

Subgroup VII is formed by two taxa, one being *S. maelanoiense* sp. nov. and the other being identified morphologically and genetically as *S. myanmarense*, originally described from Myanmar. *Simulium myanmarense* was suggested to exist in Thailand based on COI gene sequences of adult females (Jomkumsing et al. 2019). Here we confirmed the distribution of this species in Thailand by morphological and genetic evidence.

In subgroup VIII, one new species, *S. teerachanense*, is included together with two known species from Peninsular Malaysia, *S. roslihashimi* and *S. izuae*. S*imulium teerachanense* sp. nov. is morphologically similar to *S. roslihashimi* in sharing the yellowish male antennae. It is demonstrated that both species are genetically close to each other, with a difference of 2.05–2.56%.

In subgroup IX, three new species, *S. nanthaburiense*, *S. chaowaense*, and *S. muangpanense*, and one known species, *S. monglaense* from Myanmar, are included. *Simulium nanthaburiense* sp. nov. is almost identical to one of two haplotypes (MF101845) of *S. monglaense*, although both species are morphologically distinguished from each other by the number of male upper-eye (large) facets that are in 12 vertical columns and 13 or 14 horizontal rows in *S. nanthaburiense* sp. nov. but in 15 vertical columns and 15 horizontal rows in *S. monglaense* (Takaoka et al. 2017b). *Simulium monglaense* was suggested to exist in Thailand based on COI gene sequences of adult females (Jomkumsing et al. 2019). However, its distribution in Thailand has not been confirmed by the morphological evidence in our study. The possibility cannot be excluded that the COI gene sequences suspected as being those of *S. monglaense* (Jomkumsing et al. 2019) were actually those of *S. nanthaburiense* sp. nov.

## Conclusions

Our morphological study reveals a high rate of species radiation of the *S. asakoae* species group in Thailand, as evidenced by the increased species number from four to 27. Most of these species are morphologically distinguished from one another as females, males, and larvae but not pupae, as shown in the keys. Ten of 27 species were indistinguishable in the pupal stage due to similarities of key characters such as the gill, terminal hooks and cocoon, the phenomenon being contrary to other species groups of the subgenus Gomphostilbia, in which species are usually identified in the pupal stage by their distinct expression of the number and arrangement of gill filaments and/or shape of the inflated structure of the gill (Takaoka 2015).

A COI gene sequence-based analysis shows that all (but one species) in the *S. asakoae* species group in Thailand are divided into nine subgroups, of which subgroups II, V and VIII are represented by *S. asakoae*, *S. inthanonense*, and *S. teerachanense* sp. nov., respectively, and the other six subgroups are represented by two to nine species, suggesting a relatively recent radiation, accompanied by some morphological differentiation but few COI gene changes.

In the latter six subgroups, genetic differences between certain new species and known species are too small or even unrecognized, as in the cases of *S. thungchangense* sp. nov. versus *S. udomi*, *S. nanthaburiense* sp. nov. versus *S. monglaense*, and *S. sutheppuiense* sp. nov. versus *S. tanahrataense*. Certain members of the *S. asakoae* species group were previously reported to share the same or closely similar COI gene sequences, e.g., *S. udomi* versus *S. rampae* (Saeung et al. 2017, Srisuka et al. 2019), and *S. lurauense* versus *S. sofiani* (Low et al. 2015). These data indicate that certain morphospecies of the *S. asakoae* species group, such as species within subgroups I, III, IV, VI, VII, and IX, are not separable genetically from one another, at least by a COI gene sequence-based analysis.

DNA barcoding is generally useful for resolving the phylogenetic relationships among species of most species groups of the subgenus Gomphostilbia and for uncovering cryptic species. However, caution is required when members of the *S. asakoae* species group are identified based only on similarities of the COI gene sequences.

## Supplementary Material

XML Treatment for Simulium (Gomphostilbia) thungchangense

XML Treatment for Simulium (Gomphostilbia) puaense

XML Treatment for Simulium (Gomphostilbia) sutheppuiense

XML Treatment for Simulium (Gomphostilbia) teerachanense

XML Treatment for Simulium (Gomphostilbia) maewongense

XML Treatment for Simulium (Gomphostilbia) loeiense

XML Treatment for Simulium (Gomphostilbia) maelanoiense

XML Treatment for Simulium (Gomphostilbia) phapeungense

XML Treatment for Simulium (Gomphostilbia) nanthaburiense

XML Treatment for Simulium (Gomphostilbia) nanoiense

XML Treatment for Simulium (Gomphostilbia) muangpanense

XML Treatment for Simulium (Gomphostilbia) maehongsonense

XML Treatment for Simulium (Gomphostilbia) chaowaense

XML Treatment for Simulium (Gomphostilbia) pitasawatae

XML Treatment for Simulium (Gomphostilbia) banluangense

XML Treatment for Simulium (Gomphostilbia) junkumae

XML Treatment for Simulium (Gomphostilbia) kiewfinense

XML Treatment for Simulium (Gomphostilbia) huaimorense

XML Treatment for Simulium (Gomphostilbia) songense

XML Treatment for Simulium (Gomphostilbia) klonglanense

XML Treatment for Simulium (Gomphostilbia) namdanense

XML Treatment for Simulium (Gomphostilbia) myanmarense

XML Treatment for Simulium (Gomphostilbia) inthanonense

XML Treatment for Simulium (Gomphostilbia) chiangdaoense

XML Treatment for Simulium (Gomphostilbia) asakoae

## Footnotes

The females of S. banluangense sp. nov., S. kiewfinense sp. nov., S. phapeungense sp. nov., S. rampae and S. teerachanense sp. nov. are unknown.

The male of S. songense sp. nov. is unknown.

The larvae of S. banluangense sp. nov. S. nanthaburiense sp. nov., S. kiewfinense sp. nov., S. maelanoiense sp. nov., S. puaense sp. nov. and S. songense sp. nov. are unknown.
